# Proceedings 34th Symposium ESVN‐ECVN 23rd‐24th September 2022

**DOI:** 10.1111/jvim.17218

**Published:** 2024-10-29

**Selected research communications of the**

**34th Symposium of the ESVN‐ECVN**

**23rd‐24th September 2022**

***The European College of Veterinary Neurology (ECVN) Symposium and the Journal of Veterinary Internal Medicine (JVIM) are not responsible for the content or dosage recommendations in the abstracts. The abstracts are not peer reviewed before publication. The opinions expressed in the abstracts are those of the author(s) and may not represent the views or position of the ECVN. The authors are solely responsible for the content of the abstracts*.**

**TIMETABLE OF THE SYMPOSIUM**

**Table:** 

**Symposium Day 1—FRIDAY, 23rd September 2022**	
**07:15**	**Registration**
**08:15**	**Opening Ceremony and Welcome**
**08:30**	**Keynote—An update on disc‐associated spinal cord injury**
	***Dr.Natasha Olby***
**09:15**	**Keynote—Minimally Invasive surgery for thoracolumbar spinal trauma**
	***Dr. José Fernández Alen***
**10:00**	**Coffee Break & Exhibition**
**10:30**	**Oral Presentations**
	**O1 MRI and clinical findings in 133 dogs with post‐surgical recurrent neurological deficits E. Suiter**
	**O2 Hansen type I thoracolumbar intervertebral disc disease: comparison between Dachshunds and French Bulldogs T. Flegel**
	**O3 Recovery of ambulation and reduction in spinal cord compression in medically managed thoracolumbar disc extrusions in non‐ambulatory small dogs S. Khan**
	**O4 Clinical signs, causes, and outcome of central cord syndrome in 22 cats C. Ros**
	**O5 Biomechanics of the canine lumbar spine after performing multiple partial lateral corpectomies L.F. Becker**
	**O6 Comparison of standard T2‐W TSE versus vibe MRI sequences in the assessment of caudal articular process dysplasia in pug dogs with thoracolumbar myelopathy E. Gilbert**
	**O7 Experimental assessment of imaging measurements to diagnose atlantoaxial instability on computed tomography images C. Precht**
**11:45**	**Keynote—Chronic spinal cord injury: bypassing the lesion for artificial bladder control**
	***Dr. Nicolas Granger***
**12:30**	**LUNCH BREAK—EXHIBITION & POSTER DISCUSSION**
**13:30**	**Keynote—Traumatic spinal cord injury repair and regeneration**
	***Dr. José Fernández Alen***
**14:15**	**Flash Presentations**
	**FP1** Cranial thoracic myelopathies (T1–T6 vertebrae): evaluation of the signalment, clinical presentation and diagnoses in 84 dogs B. Lopes
	**FP2** Non‐traumatic haemorrhagic myelopathy in dogs A. Fernandez
	**FP3** Clinical characterization of thoracolumbar intervertebral disc extrusions in Basset Hounds
	H. Thatcher
	**FP4** Instrumented cervical fusion using patient‐specific custom designed interbody distraction‐fusion devices in seven dogs with disc‐associated cervical spondylomyelopathy C. Driver
	**FP5** Clinical presentation and longterm outcome following urgical treatment of cervical spinal arachnoid diverticula in dogs D. Ryan
	**FP6** Cervical intervertebral disc disease in 60 Yorkshire Terriers
	V. Palus
	**FP7** Discospondylitis in dogs: inflammatory biomarkers in relapse nd prognosis, a retrospective cohort study of 149 dogs G. Galli
	**FP8** Association between gait speed and cognition in aging dogs A. Mondino Vero
	**FP9** Diagnostic accuracy of cerebrospinal fluid lactate levels in cattle with neurological disorders
	S. Ferrini
	**FP10** Establishment of a companion animal brain bank in the United Kingdom T. Cardy
	**FP11** A prospective study on clinical signs, management, outcomes, and delayed neurologic sequelae due to metaldehyde poisoning in 26 dogs G. Dutil
	**FP12** Decompression of extensive bilateral subarachnoid hematomas in dogs and cats with hemispheric collapse after ventriculoperitoneal shunting and overdrainage A. Czerwik
	**FP13** Clinical characterization of paroxysmal dyskinesia in Dachshunds N. Alza Salvatierra
	**FP14** Non‐invasive prediction of presumed intracranial hypertension: a prospective study in 60 dogs V. Gonzalo‐Nadal
	**FP15** Clinical findings, magnetic resonance imaging, features and clinical outcome in a population of dogs and cats with porencephaly and hydranencephaly C. Maeso
**15:30**	**Coffee Break & Exhibition**
**16:00**	**Annual General Meeting**
**17:30**	**Adjourn**
**Symposium Day 2—SATURDAY, 24th September 2022**	
**08:30**	**Oral Presentations**
	**O8** Dynamics of changes in dti values on the course of the spinal cord on the porcine model K. Owsinska‐Schmidt
	**O9** Using AI to map mid‐sagittal neuromorphological changes in Chiari‐like malformation associated pain and syringomyelia in the Cavalier King Charles Spaniel J. Cumber
	**O10** Spontaneous vocalization in dogs presenting for neurological evaluation between 2016 and 2022: a descriptive study A. Spencer‐Taylor
	**O11** Plasma mitochondrial and nuclear DNA as a potential early biomarker of progressive myelomalacia in dogs: preliminary study J.M. De Frias
**09:10**	**Short flash break**
**09:15**	**Keynote—Spinal cord injury and neuroimaging. What is new in 2022 Francisco Llabrés Díaz**
**10:00**	**Coffee Break & Exhibition**
**10:30**	**Keynote—Treatment of paralysis in dogs Natasha Olby**
**11:15**	**Oral Presentations**
	**O12** Spinocerebellar ataxia in the bouvier des ardennes breed is caused by the KCNJ10 C.986 T>C variant, previously reported in the Belgian Malinois Breed K. Stee
	**O13** Phenobarbital treatment in canine idiopathic epilepsy: effects on microbiota, its functions and behavioral comorbidities A. Watanangura
	**O14** Diagnosis of electrographic seizures and nonconvulsive status epilepticus in dogs and cats that underwent EEG after ceasing of clinical seizure activity: 26 cases C. Taestensen
	**O15** CSF‐specific oligoclonal bands in dogs with idiopathic epilepsy J. Föhr
	**O16** Tumor‐Associated Antigens (TAA) in canine brain tumors for use in adjunctive immunotherapy after surgery W. Pownall
**12:05**	**Short flash break**
**12:10**	**Flash Presentations**
	**FP16** The effect of oral zonisamide treatment on serum phenobarbital concentrations in epileptic dogs E. Mahon
	**FP17** Myoclonus associated with cerebellar signs in steroid‐responsive tremor syndrome in two dogs**—**Electroencephalographic findings L. Brewińska
	**FP18** Antibody binding pattern in dogs with steroid‐responsive meningitis‐arteritis
	Proof of principle J. Nessler
	**FP19** Fido's brain on fire: anti‐gabaa receptor encephalitis in a dog E. Hunerfauth
	**FP20** Rating neurological impairment in canine meningoencephalitis: the neurodisability scale
	R. Goncalves

## FLASH PRESENTATIONS

## (FP 01)

### Cranial thoracic myelopathies (T1‐T6 vertebrae): Retrospective evaluationof the signalment, clinical presentation and diagnoses in 84 dogs

#### B. A. Lopes, DVM^1^ , E. Ives, MA, VetMB^1^ , R. José‐López, DVM, PhD^2^ ,R. Gutierrez‐Quintana, MVZ, MVM^2^ , J. Abouzeid, DVM^3^ , P. Freeman, MA, VetMB^3^ , J. I. Redondo, Ldo, MVM, PhD^4^ , D. Sánchez‐Masián, DVM^1^

##### ^1^Anderson Moores Veterinary Specialists, Hursley, Winchester, United Kingdom; ^2^School of Veterinary Medicine, College of Medical, Veterinary and Life Sciences, University of Glasgow, Glasgow, United Kingdom; ^3^Department of Veterinary Medicine, University of Cambridge, Cambridge, United Kingdom; ^4^Universidad CEU Cardenal Herrera, Valencia, Spain

The aim of the study was to characterize the signalment, clinical presentation and diagnoses referable to clinically significant lesions affecting the cranial thoracic (T1–T6) vertebral column identified on advanced imaging. Retrospective evaluation of the databases of three veterinary referral centres, between 2009 and 2021, was performed to identify dogs with a lesion affecting the cranial thoracic vertebral column (T1–T6 vertebrae) that was the primary cause for presenting signs of myelopathy and/or spinal pain. Eighty‐four dogs were included in the study, with the majority presenting with a progressive history of over four‐weeks duration. On neurologic examination, most dogs were ambulatory (*n* = 64) and the most common neuroanatomic localisation was the T3–L3 spinal cord segments (*n* = 63). Twelve dogs (14.3%) showed a short‐ strided thoracic limb gait on clinical examination. The most common diagnosis was neoplasia (*n* = 33, with a mesenchymal tumor diagnosed or suspected in 23 dogs), followed by anomalies (*n* = 22, including vertebral malformations in 14 dogs) and degenerative disorders (*n* = 16, with intervertebral disc protrusion diagnosed in 9 dogs). The most common vertebrae affected were T3 and T5. Dogs with degenerative conditions were more likely to show asymmetric clinical signs, and the majority of dogs with neoplasia showed signs of spinal hyperaesthesia on examination. The findings of this study describe the clinical signs and final diagnoses associated with lesions affecting the cranial thoracic vertebral column. When combined with the signalment and clinical history, this information can assist in both the recognition of and clinical reasoning in such cases.

## (FP 02)

### Non‐traumatic haemorrhagic myelopathy in dogs

#### N. West, BVMS MRCVS ^1^, S. Butterfield, BSc(Hons), BVSc, PGDip(VCP), MRCVS ^2^, S. Archer, BVMS, CertCHP, MSc, PhD, DipECVPH, MRCVS, EBVS, C. Rusbridge, BVMS (Hons), PhD, DECVN, FRCVS^3^ ^,4^, D. Whittaker, BSc(Hons), BVetMed(Hons), MVetMed, PhD, DipECVN, MRCVS^1^ , A. Fernandez Cid MRCVS^1^

##### ^1^Fitzpatrick Referrals Orthopedics and Neurology, Halfway Lane, Eashing, Godalming, Surrey, GU7 2QQ, UK; ^2^Queen Mother Hospital for Animals, Clinical Science and Services, Hawkshead Lane, North Mymms, Hatfield, AL9 7TA, UK; ^3^The University of Surrey, 388 Stag Hill, Guildford GU2 7XH, UK; ^4^Wear Referrals Veterinary Hospital, Stockton‐On‐Tees TS2^1^2ES, UK

Introduction—Non‐traumatic spinal cord hemorrhage is an uncommon cause of myelopathy in dogs. The study purpose was to describe the clinical characteristics, MRI findings, concurrent medical conditions and outcome in dogs diagnosed with non‐traumatic haemorrhagic myelopathy. Methods‐ Inclusion criteria was a diagnosis of non‐traumatic haemorrhagic myelopathy based on characteristic MRI features performed within 24 h of presentation, and/or histopathological confirmation of hemorrhage. Dogs were excluded if they had a history of trauma, a diagnosis of an intervertebral disc extrusion, incomplete medical records or insufficient follow‐up data. Study Design—This is a retrospective descriptive study between 2013 and 2021 from two referral hospitals. Results—Twenty‐ two dogs were included. The most common etiology was idiopathic (*n* = 9; 41%). An underlying medical condition was identified in the remaining 59% of cases, with Angiostrongylus vasorum representing 30.7% of this group. 61% of dogs had a good/excellent outcome, irrespective of etiology and severity of neurological signs. Cases with a poor outcome included a cavernous sinus malformation, hemophilia A, radiation induced hemorrhage and acute lymphoid leukemia. The mean length of spinal cord oedema was four times higher in dogs with a poor outcome in comparison to dogs with an excellent outcome, however this was not statistically significant. Owning to the low power of the study (<20%), and it remains possible observed associations are due to random noise. Conclusions: This study prompts the necessity to investigate for underlying medical conditions for cases of non‐ traumatic haemorrhagic myelopathy. Further studies are warranted to conclude prognostic indicators.

## (FP 03)

### Clinical characterization of thoracolumbar intervertebral disc extrusions in basset hounds

#### H. Thatcher, BVM, BVS, MRCVS^1^ , E. Alcoverro, DVM, DipECVN, MRCVS^2^ ,K. Stee, DVM, Dip ECVN, MRCVS^3^ , I. Schofield, BVSc, MSc(VetEpi), MRCVS^4^ ,M. Target, MA, VetMB, PhD, DipECVN, SFHEA, MRCVS^5^ ,M. Lowrie, MA, VetMB, MVM, DipECVN, MRCVS^1^ , S. A Gomes, DVM, DipECVN, MRCVS^1^

##### ^1^Dovecote Veterinary Hospital, Derby, UK; ^2^Chestergates Veterinary Specialists, Cheshire, UK; ^3^Department of Medicine and Clinical Biology of Small Animals, Faculty of Veterinary Medicine, University of Ghent, Ghent, Belgium; ^4^CVS Referrals, Diss, UK; ^5^School of Veterinary Medicine and Science, University of Nottingham, Loughborough, UK

Intervertebral disc extrusion (IVDE) is a frequent condition in dogs, with chondrodystrophic breeds being overrepresented. Breed‐specific studies describing IVDE are lacking and there is currently no published data specifically assessing Basset Hounds (BH). BH is the largest among the chondrodystrophic breeds, one of the breeds with an important penetration of the FGF4 retrogene on chromosome 12. We aim to describe and compare the signalment, anatomical location and clinical severity of thoracolumbar IVDE between BH and Dachshunds. Retrospectively, medical histories of IVDE‐affected Dachshunds and BH was performed. Information collected included signalment, duration of clinical signs, affected discs and anatomical location, neurological grading at presentation and discharge. A total of 154 Dachshunds and 68 BH were available for analysis and comparison. The majority of BH presented with IVDE affecting discs between T9‐L2 (66.2%), with a mean age of 87.5 months, mostly presenting with non‐ ambulatory paraparesis and a male predilection. Statistical significance analysis (p < 0.05) indicated that BH presented with an older age (mean 87.5 vs. 66.5 months), had a greater proportion of L2‐ S1 lesions, and were more frequently ambulatory at discharge than Dachshunds. Interestingly, only one BH was paraplegic without sensation (1.5%), compared with 16 Dachshunds (10.4%). Basset hounds seem to present IVDE at an older age, greater proportion of L2‐ S1 lesions and were more frequently ambulatory at discharge than Dachshunds. Understanding the characteristics of IVDE in distinct breeds could help recognize links between environmental and genetic factors, as well as define preventive strategies (eg, screening at specific time points).

## (FP 04)

### Instrumented cervical fusion using patient‐specific custom designed interbody distraction‐fusion devices in seven dogs with discassociated cervical spondylomyelopathy

#### C. Driver, BSc BVetMed MVetMed PhD DipECVN MRCVS^1^ , V. Lopez, BSc^2^ ,^3^, ^3^,^4 3 5^ B. Walton, BVSc DSAS(Orth) MRCVS, D. Jones, BSc PhD, R. Fentem, BVSc AFHEA MRCVS A. Tomlinson, BVSc CertAVP(GSAS) DipECVS MRCVS^5^ , J. Rose, MA VetMB DipECVN AFHEA MRCVS^1^

##### ^1^Lumbry Park Veterinary Specialists, Alton, UK; ^2^School of Engineering, University of Liverpool, Liverpool, UK; ^3^Fusion Implants, Liverpool, UK; ^4^Movement Referrals, Chester, UK; ^5^Small Animal Teaching Hospital, University of Liverpool, Liverpool, UK

Our aim was to report the medium‐term outcome of instrumented vertebral fusion in dogs with disc‐associated cervical spondylomyelopathy (DA‐CSM), using patient‐specific custom‐designed titanium interbody devices. Medical records of dogs with DA‐CSM at two institutions between 2019 and 2022 were reviewed. Follow‐up included repeat examination, neurologic grading, and CT. Seven adult (80 months, 62–123) large breed (41.6 kg, 28.4–56.9) dogs with 11 affected disc‐spaces were included. These occurred at C5‐6 (one dog), C6‐7 (two dogs) or both levels (four dogs). The commonest presenting complaints were ambulatory generalized ataxia, paresis, and neurogenic lameness. In‐silico manipulation of pre‐ operative CT scans facilitated the additive manufacture of woven titanium interbody devices that conformed to patient vertebral end‐plate morphology. Interbody devices were implanted alongside bone graft and screws and cement (3 dogs), paired locking plates (3 dogs) and a polyaxial screw‐rod system (1 dog). Follow‐up CT was performed in six dogs at median 3 months (range 2–11) and examination at 10.8 months (±3.13 months). Vertebral fusion was evident at 8/10 sites. There were no major complications, and no implants were removed. Subsidence was observed at 4/10 sites without clinical relevance. 5/6 dogs improved. One dog deteriorated but was subsequently diagnosed with immune‐mediated polyarthropathy. One dog died 24 months post‐operative due to an unrelated disease. Instrumented distraction‐fusion using a custom interbody device in dogs with DA‐CSM was achieved with satisfactory medium‐term outcomes with no major complications. The device design facilitated CT‐determined fusion, with no or mild vertebral subsidence.

## (FP 05)

### Clinical presentation and longterm outcome following surgical treatment of cervical spinal arachnoid diverticula in dogs

#### D. Ryan, BVM BVS BVMedSci AFHEA MRCVS^1^ ^,2^, G. Bruto Cherubini, DVM dipECVN FRCVS^1^

##### ^1^Neurology and Neurosurgery Department, Dick White Referrals, Station Farm, Six Mile Bottom, Cambridgeshire, United Kingdom; ^2^Queen Mother Hospital for Animals, Royal Veterinary College, University of London, Hertfordshire, United Kingdom

There are a number of publications documenting surgical treatment, with short‐ and long‐term outcome, of thoracolumbar spinal arachnoid diverticula (SAD). However, the literature on cervical SAD, especially long‐term outcome, is limited with only a few cases. The aim of this study was to investigate the clinical presentation and short‐ and long‐term outcome in animals undergoing surgical treatment for cervical SAD. The clinical records of 32 dogs following surgical treatment of cervical SAD were retrospectively evaluated. Dogs were included if they had complete clinical records and included in long term outcome if there was a follow‐up of at least 6 months. Outcome was considered as improved, if the neurological signs were improved compared to pre‐op, static or deteriorated. Males (68.8%) were more commonly affected than females, and the Pug (46.9%) was the most common breed. Diverticula were located dorsally at C2–C3 in 65.6% of cases. Long term follow up was available in 27 dogs (median follow up time 33 months, range 6–86). Overall, long term outcome was graded as improved in 55.6% of patients, static in 33.3% and deteriorated in 11.1%. Recurrence of clinical signs was common, occurring in 63% (17/27) of cases, however 29.4% (5/17) remained improved compared to pre‐surgery. Deterioration occurred a median of 5 months post‐surgery (range 1–26 months). Overall this study suggests dogs can have a fair to good long‐term outcome, but recurrence of clinical signs is common.

## (FP 06)

### Cervical intervertebral disc disease in 60 yorkshire terriers

#### V. Palus, Dipl.ECVN^1^ ^,2^, L. Stehlik, PhD^2^ , A. Necas, PhD, MBA^2^ , D. Lu, BVetMed, MVM, Dipl.ECVN, CVA^3^

##### ^1^Neurovet, Trencin, Slovakia; ^2^University of Veterinary Sciences, Brno, Czech Republic; ^3^CityU Veterinary Medical Centre, Kowloon, Hong Kong

Intervertebral disc disease (IVDD) is a common neurological disorder in dogs. A precise description of features of IVDD was described in different breeds. Our hypothesis was that Yorkshire terriers (YTs) were commonly presented with cervical IVDD. Our aim was to present the findings in YTs with cervical IVDD. A retrospective study was performed on YTs presented with cervical IVDD confirmed with surgery from two referral practices between 2005 and 2021. Medical reports were reviewed, with analysis of signalment, neurological examination, lesion localisation, surgical site correction and recovery after surgery. Other YTs referred for neurological investigation to one referral center between 2016 and 2021 were also collected for comparison. The cervical IVDD group consisted of 60 YTs (4 (6.67%) females, 9 (15.0%) neutered females, 33 (55.0%) males, and 14 (23.33%) neutered males). The mean (±SD) weight was 5.04 (±1.765) kg. The mean (±SD) age was 9.35 (±2.945) years. The most affected and surgically treated disc space was C4‐C5 (29/60, 39.19%). Fourteen (23.33%) dogs had more than one treated space. Forty‐six (76.67%) cases had complete and 14 (23.33%) incomplete recovery. The mean time (±SD) to discharge and ambulation was 2.93 (±2.331) and 3.32 (±7.373), respectively. Seven (11.67%) dogs had relapses. The group of YTs with other neurological diagnoses consisted of 277 animals. Statistical analysis revealed that younger dogs had a better outcome. The prevalence of YTs with cervical IVDD was 10.06% among all the YTs with neurological disorders. Cervical IVDD is a common neurological condition in the population of YTs.

## (FP 07)

### Discospondylitis in dogs: Inflammatory biomarkers in relapse and prognosis, a retrospective cohort study of 149 dogs

#### G. Galli, DVM^1^ , A. Zoia, DVM, PhD, Dipl ECVIM‐CA^2^ , L. Ventura, Full Professor^3^, M. Menchetti, DVM, PhD, Dipl ECVN^1^

##### ^1^Division of Neurology and Neurosurgery, San Marco Veterinary Clinic, Veggiano (PD), Italy; ^2^Division of Internal Medicine, San Marco Veterinary Clinic, Veggiano (PD), Italy; ^3^Department of Statistical Sciences, University of Padova, Padova (PD), Italy

Discospondylitis is a common disease in dogs caused infectious agents. The prognosis is good in particular for bacterial infections both in human and veterinary medicine. The purpose of this study is to identify biomarkers of relapse and prognostic indicators in a population of dogs with discospondylitis.One‐hundred and forty‐nine dogs presented between 2005 and 2019 with a confirmed diagnosis of discospondylitis, complete medical records comprehensive of bloodwork and urine culture when available, were retrospectively included. Bloodwork performed at the time of diagnosis were compared between dogs that experienced a relapse and dogs that did not experience a relapse and between dogs that died because of discospondylitis and dogs that survived or died for unrelated causes. The 12.7% of dogs that relapsed had total leukocyte count, C‐reactive protein and fibrinogen concentrations were significantly increased (*P* = 0.028, *P* = 0.005, and *p* = 0.03 respectively), while antithrombin concentration was significantly decreased (*P* = 0.014) compared with dogs that did not relapse. Urine culture was significantly more negative in dogs that did not relapse compared to dogs that experienced a relapse (93% and 73% respectively, *P* = 0.023). The 14.8% of dogs that died for causes related to discospondylitis had significantly higher total leukocyte, neutrophils and monocyte counts, increased ferritin and IgA concentrations and activated prothrombin time (*P* = 0.007, *P* = 0.004, *p* = 0.02, *P* = 0.036, *P* = 0.04, and *p* = 0.04 respectively), while basophil count, albumin, antithrombin and TIBC concentrations were significantly lower (*P* = 0.0004, *P* = 0.04, *p* = 0.01, and *p* = 0.0002, respectively). Leukocyte count and specific APPs may represent useful biomarkers in predicting the relapse and prognosis of discospondylitis.

## (FP 08)

### Association between gait speed and cognition in aging dogs

#### A. Mondino Vero, DVM, MSc, PhD^1^ , M. Khan, DVM^1^ , G. Fefer, DVM^1^ , W. Panek, DVM^1^ , B. Case, MSc^1^ , B. D. X Lascelles, BSc, BVSc, PhD, FRCVS, CertVA, DSAS(ST), DECVS, DACVS^1^ , M. Gruen, DVM, MVPH, PhD, DACVB^1^ , N. Olby, Vet MB, PhD, MRCVS, DACVIM (Neurology)^1^

##### ^1^Department of Clinical Sciences, North Carolina State University, Raleigh, United States

Introduction: Physical and cognitive performance decline with age. In humans, slow gait speed is significantly associated with higher risk of cognitive decline. Dogs share many similar features of age‐related cognitive decline with their human counterparts. We hypothesized that there would be an association between gait speed and cognitive function in aging dogs. Methods: 49 senior dogs (13.09 ± 1.73 years old) participating in the Neuroaging Program were included. On leash and off leash (moving toward a treat) gait speed were measured. Cognitive function was assessed with the CAnine DEmentia Scale (CADES) and cognitive tests that evaluate attention (sustained gaze test), working memory, inhibitory control, and problem‐solving (detour test). Results: Mean gait speed on and off leash were 0.78 ± 0.20 and 1.38 ± 0.64 m/s, respectively. Gait speed off leash was significantly lower in dogs with moderate or severe cognitive dysfunction (0.97 ± 0.45 m/s) in comparison with normal or mildly impaired dogs (1.59 ± 0.62 m/s) as classified by CADES (*P* = 0.002). While gait speed on leash was only correlated with performance on inhibitory control (*r* = 0.30), gait speed off leash was significantly (*p* = 0.05) positively correlated with greater cognitive function (r = 0.48, *r* = 0.37, *r* = 0.50, and *r* = 0.38, for sustained gaze, memory, inhibitory control, and detour tests, respectively). Conclusion—Gait speed off leash is positively associated with better cognitive performance in older dogs, further studies should evaluate if its decline could have a prognostic value in assessing risk of cognitive deterioration.

## (FP 09)

### Diagnostic accuracy of cerebrospinal fluid lactate levels in cattle with neurological disorders

#### S. Ferrini, DVM, MRCVS, PhD Student, ECVN Resident^1^,G. Cagnotti, DVM, MRCVS, PhD Student, Dip. ECVN^1^ , U. Ala, MSc in physics, PhD^1^ ,C. Bellino, DVM, PhD^1^ , E. Biasibetti, DVM, PhD^2^ , G. Borriello, DVM, PhD^1^ , C. Corona, DVM^2^ , G. Di Muro, DVM, PhD Student^1^, G. Iamone, DVM, PhD Student^1^, B. Iulini, DVM^2^ ,M. Pezzolato, DVM^2^ , A. D'Angelo, DVM, PhD, Dip. ECVN, Dip. ECBHM^1^

##### ^1^Department of Veterinary Science, University of Turin, Grugliasco, Italy; ^2^Istituto Zooprofilattico del Piemonte Liguria e Valle d'Aosta, Turin, Italy

Introduction: Cerebrospinal fluid (CSF) analysis can be useful for the diagnosis of some neurological diseases. Determination of CSF lactate has proven helpful in the diagnosis of bacterial central nervous system (CNS) infections in human medicine. The aims of this study were to define a range of normality of L‐Lactate in bovine CSF and to evaluate if its concentration could help in differentiating bacterial infections from other neurological disorders. Methods: CSF L‐lactate was evaluated in a group of healthy cattle (group A, *n* = 34) and in a group of cattle with CNS disorders (group B, *n* = 57). All underwent physical examination, neurologic examination, CSF and blood analysis. Reference Interval (RI) for CSF L‐Lactate concentration in group A was calculated with bootstrap method. Concerning group B, subgroups were formed based on the interpretation of CSF analysis and analyzed with Kruskal‐Wallis test and subsequent pairwise comparisons. Receiving operator characteristic (ROC) analysis was performed considering CSF L‐lactate of each subgroup versus the others. Results: The RI for L‐lactate in the CSF of healthy cattle was 0.9–2.5 mmol/L. The CSF L‐lactate concentration was significantly higher in cattle with neutrophilic pleocytosis. The best ROC curve obtained was the one that considered neutrophilic pleocytosis versus the others: at the cut off 3.15 mmol/L, L‐lactate showed diagnostic sensitivity and specificity of 91% and 80% respectively, with area under the curve of 0.89. Discussion: Based on the results of the present study, measurement of CSF L‐lactate could provide a rapid diagnosis for most CNS bacterial infections.

## (FP 10)

### Establishment of a companion animal brain bank in the United Kingdom

#### T. Cardy, BSc BVetMed(Hons) MVetMed PhD DipECVN MRCVS^2^ ^,1^, A. Crawford, BVM &S BSc PhD MVetMed DipECVN MRCVS^1^

##### ^1^Royal Veterinary College, North Mymms, United Kingdom; ^2^Cave Veterinary Specialists, Wellington, United Kingdom

Introduction: A Companion Animal Brain Bank (CABB) was established at the Royal Veterinary College in 2018 to provide free access to high quality tissue samples for neuroscience research. Methods: A standardized methodology adapted from the Medical Research Council's (MRC) Brain Bank Network is used to ensure systematic sample collection, processing, storage and cataloguing of fresh frozen and formalin‐fixed paraffin embedded brain tissue. RESULTS: Forty‐three brains had been archived at the time of writing (33 dogs and 10 cats) alongside residual samples of blood, cerebrospinal fluid and urine (where available). Eight brains from normal (control) animals of a range of ages (2 cats: 1 and 10 yo, and 6 dogs: 1, 2, 5, 7, 9 and 14 yo) have been archived. The remaining samples represent a range of neurological diseases including inflammatory (necrotising leukoencephalitis, necrotizing meningoencephalitis, granulomatous meningoencephalitis, LGI1‐autoantibody mediated limbic encephalitis), infectious (feline infectious peritonitis, *Ehrlichia encephalitis* and vasculitis, and *Cryptococcus meningoencephalitis*), vascular (cerebral hemorrhage and infarction), neoplastic (lymphoma, metastatic pulmonary adenocarcinoma, astrocytoma, meningioma, histiocytic sarcoma, multilobular tumor of bone, and pulmonary adenoma), idiopathic epilepsy with status epilepticus and global brain hypoxic‐ischaemic injury. DISCUSSION:IN a short space of time the CABB has archived samples from multiple species with a diverse range of neurological conditions. These samples are available for use by neuroscience researchers nationally and internationally to further research in this area.

## (FP 11)

### A prospective study on clinical signs, management, outcomes, and delayed neurologic sequelae due to metaldehyde poisoning in 26 dogs

#### G. Dutil, Dr.^1^,^2^, P. Berny, Dr., Pr., PhD^3^

##### ^1^Division of Neurology & Neurosurgery, CHV Atlantia, Nantes, France; ^2^Division of Clinical Neurology, Department of Clinical Veterinary Medicine, Vetsuisse Faculty, University of Bern, Bern, Switzerland; ^3^Division of Pharmacology & Toxicology, VetAgro Sup, Marcy l'Etoile, France

To prospectively describe clinical signs, therapeutic management, outcomes, and delayed‐onset seizures due to metaldehyde poisoning in dogs. A 15‐month prospective study on dogs with confirm or witness diagnosis of metaldehyde poisoning, either via phone call to the animal poison control centre or analysis at the toxicology laboratory in Lyon, France. Clinical signs, therapeutic management and outcomes and the late onset of seizures were assessed for at least 3 years. Twenty‐six dogs were enrolled in the study. The most prevalent clinical signs were ataxia (18 dogs), convulsions (17), hypersalivation (15), and tremors (15). Treatment was symptomatic (eg, activated charcoal, emetic therapy, and IV fluids) with anticonvulsant therapy (mainly diazepam). The overall survival rate was 81% (21/26 dogs). All dogs that received active charcoal (11/11) or emetic therapy (4/4) survived (higher survival with activated charcoal, *P* < 0.05). Twelve of 17 dogs had convulsions and survived; 9 were followed up for at least 3 years after poisoning, and none had any other seizure episode or neurological sequelae. This prospective study describes clinical signs, therapeutic management and outcome of metaldehyde poisoning in dogs and late onset neurologic sequelae. None of the 9 cases that were followed for 3 years developed any neurological signs after metaldehyde poisoning. Therefore, long‐term antiepileptic therapy is not indicated.

## (FP 12)

### Decompression of extensive bilateral subarachnoid hematomas in dogs and cats with hemispheric collapse after ventriculoperitoneal shunting and overdrainage—Preliminary report

#### ^1^ A. Czerwik, Phd, A. Olszewska, Dr, D. Farke, Dr, Dipl ECVN, M. J. Schmidt, Prof, Dr, Dipl ECVN

##### ^1^Department of Veterinary Clinical Sciences, Small Animal Clinic, Justus‐Liebig‐University, Giessen, Germany

Communicating internal hydrocephalus is the most common malformation in dogs and cats. Treatment of choice is the surgical placement of a ventriculoperitoneal shunt (VPS). In animals as well as in humans, overshunting VPS placement is one possible complication resulting in hemispheric collapse, often with fatal outcome. Large breed dogs with largely dilated ventricles and thin cerebral cortex may be especially predisposed to overshunting. The aim of this case series was to present animals that underwent decompressive surgery after hemispheric collapse as a complication of VPS placement. Seven dogs and two cats were included. Acute deterioration occurred in animals within 4–46 days after VPS placement. Clinical signs include obtundation, ataxia, circling, nystagmus or seizures. Overshunting was diagnosed by magnetic resonance imaging of the brain in all cases. All animals underwent single or bilateral craniotomy to evacuate the accumulated cerebrospinal fluid and hemorrhage from the subarachnoid space. Four animals received additional suboccipital craniectomy. The ventricular catheter of the VPS remained within the ventricular space, except for one cat in which the VPS was entirely exchanged. Three dogs and one cat improved completely showing no more deficits, four dogs improved, but showed further deficits. One cat showed no improvement and was euthanized. The follow‐up after the surgical intervention was 1–36 months. The current case series describes possible emergency intervention in cases with overshunting and hemispheric collapse and shows that despite deteriorated neurological condition, the prognosis may be favorable.

## (FP 13)

### Clinical characterization of paroxysmal dyskinesia in dachshunds

#### D. N. Alza Salvatierra, Ldo Vet MRCVS^1^ , L. De Risio, PhD PGCert Vet Ed DipECVN FRCVS^2^ , S. Bertram, DipECVN MRCVS^3^ , S. Gilbert, BVSc BSc MRCVS^3^ ,B. A. Santos Lopes, DVM GPCert(Neuro) MRCVS^4^ , S. Fitzmaurice, BVSc DipACVIM(Neuro) DipECVN MRCVS RCVS Specialist (Neuro)^5^, C. Stan, DVM MRCVS^1^ , L. Motta, DVM(Hons) DipECVN MRCVS RCVS Specialist (Neuro)

##### ^1^Northwest Veterinary Specialists, Runcorn, UK; ^2^Linnaeus Veterinary limited, UK, UK; ^3^Cave Veterinary Specialists, Wellington, UK; ^4^Anderson Moores Veterinary Specialists, Winchester, UK; ^5^Eastcott Referrals, Swindon, UK

The present study aimed to document a novel paroxysmal movement disorder in Dachshunds. Medical records of 15 Dachshunds presumptively diagnosed with paroxysmal dyskinesia between January 2020 and March 2022 were reviewed. All dogs were normal between episodes. A detailed description of the episodes was available for all dogs, and video footage was available for 9/15 dogs. Episodes were characterized by tremors and dystonia in =1 limb leading to occasional collapse, with preserved consciousness. During, before or after the episodes, 6 dogs presented vomiting, 4 defecation, 3 hypersalivation and/or 2 urination. Median age of onset was 26 months (range, 7–82 months). Median episodes' frequency was one every 4.5 weeks (range, 1–14 weeks). Episode duration ranged from 1 to 20 min in 14/15 dogs, with one dog experiencing 12–14 hour episodes. Neurological examinations were unremarkable. Eleven dogs underwent MRI; 10 were unremarkable, and the remainder had bilateral caudate nuclei lesions. CSF analysis was normal in all tested dogs (3). Anti‐gliadin IgG and transglutaminase‐2 IgA antibodies serum concentration were tested in 8 dogs, and 5 had one or both markers higher than the reference range established for Border Terriers. Follow‐up was available in 11/15 dogs (median 199 days, range 58‐455 days). Antiepileptic drugs were used in three dogs, only one treated with phenobarbital improved the episode frequency. Five dogs started gluten‐free diet, and 2 dogs had a reduction in episode frequency. Most owners believe that the dog's quality of life was good. Further research on the pathophysiology and natural course of this novel paroxysmal dyskinesia is warranted.

## (FP 14)

### Non‐invasive prediction of presumed intracranial hypertension: A prospective study in 60 dogs

#### V. Gonzalo Nadal, DVM MRCVS^1^ , R. Gutierrez Quintana, MVZ MVM DipECVN MRCVS^1^ , M. Czopowicz, DVM PhD^2^ , P. Amengual Batle, DVM DipECVN MRCVS^3^ ,G. Hammond, MA VETMB MVM CertVDI DipECVDI FHEA MRCVS^1^ ,R. Jose Lopez, DVM DipECVN PhD MRCVS^14^

##### ^1^Division of Small Animal Clinical Sciences, School of Veterinary Medicine, College of Medical, Veterinary and Life Sciences, University of Glasgow, Glasgow, United Kingdom; ^2^Division of Veterinary Epidemiology and Economics, Institute of Veterinary Medicine, Warsaw University of Life Sciences—SGGW, Warsaw, Poland; ^3^Hospital Veterinario Puchol, Madrid, Spain; ^4^Hamilton Specialist Referrals—IVC Evidensia, High Wycombe, United Kingdom

This prospective study aimed to evaluate transcranial Doppler measurements of the basilar artery, optic nerve sheath diameter, and optic papilla (OP) elevation as non‐invasive methods of prediction of increased intracranial pressure (ICP) in dogs. Magnetic resonance imaging (MRI) was used as the standard reference and dogs undergoing MRI of the brain were assigned a score based on indicators previously associated with higher direct ICP measurements. Sixty dogs were included. Dogs were divided into three groups: (1) normal brain (*n* = 27), (2)structural lesion/s without signs of suspected raised ICP (*n* = 17), and (3) structural lesion/s with signs of suspected raised ICP (*n* = 16). A clinical severity score was also provided for each dog. Multivariable logistic regression analysis was performed. The clinical score was more severe in dogs with an MRI score suggestive of raised ICP (*P* < 0.001). In dogs with presumptively increased ICP, the OP was significantly elevated both on MRI (*P* = 0.008) and optic ultrasound (OUS) (*P* < 0.001). A positive correlation was observed between OP elevation and higher MRI scores using both MRI and OUS; with OUS yielding more accurate results than MRI. On MRI, dorsal 3D‐constructive interference steady‐state sequence identified OP elevation in a significantly higher number of dogs than T2‐weighted images. None of the other methods differed between dogs with and without presumptively raised ICP. End‐tidal CO2 and mean arterial pressure did not influence these evaluations. Optic papilla elevation significantly correlates with MRI signs of raised ICP. Its identification, particularly through OUS, might represent a readily available method for diagnosing increased ICP in dogs.

## (FP 15)

### Clinical findings, magnetic resonance imaging features, and clinical outcome in a population of dogs and cats with porencephaly and hydranencephaly

#### C. Maeso, DVM^1^ , T. Heredia, DVM^2^ , S. Moya, DVM^3^ , A. Escudero, DVM^4^ , J. M. Segura, DVM^5^ , J. J. Sanchez, DVM^6^ , S. Rodenas, DVM, Dip. ECVN^2^

##### ^1^AniCura Ars Veterinaria Veterinary Hospital, Barcelona, Spain; ^2^AniCura Valencia Sur Veterinary Hospital, Valencia, Spain; ^3^Animal Bluecare Veterinary Hospital, Malaga, Spain; ^4^Aralar Veterinary Hospital, Zaragoza, Spain; ^5^Nexo Novelda Veterinary Clinic, Alicante, Spain; ^6^La rambla de siete palmas Veterinary Clinic, Las palmas, Spain

Porencephaly and hydranencephaly are congenital cerebral cavities, which may communicate with the lateral ventricles and/or subarachnoid space (SS). In veterinary literature, detailed description of these findings is limited to few case‐series reports including a low number of dogs and cats. The purpose of this case series retrospective study was to describe the clinical and magnetic resonance imaging (MRI) findings, in a population of dogs and cats with diagnosis of porencephaly or hydranencephaly, as well as their clinical outcome. Medical records and imaging findings of dogs and cats with diagnosis of porencephaly/hydranencephaly, and brain MRI (0.2 T or 1.5 T) performed between 2016 and 2022 were retrospectively reviewed. Signalment, chief complaint, neurological examination, MRI findings, and clinical outcome were assessed. Twelve dogs and 12 cats were included. Epileptic seizures (ES) was the most common clinical sign observed (15/24), especially in cats (10/12). Neuroanatomic localization was non‐lateralized forebrain (13/24), lateralized forebrain (6/24), and multifocal intracranial (5/24). Porencephaly was detected in 22/24 animals, considering bilateral in 4/22 and unilateral in 18/22; communicating with ventricles and SS in 17/22 and 8/22, respectively; and associated with other intracranial developmental disorders in 10/22 animals. Hydranencephaly was detected in 2/24 animals. Glucocorticoids and antiepileptic drugs were instituted in 14/24 and 16/24 animals, respectively. Finally, 17/24 animals presented improvement of the neurological signs in the follow up, especially regarding to ES (14/15). Results of this study suggest that ES, forebrain localization, and unilateral ventricles communicating porencephaly were the most clinical and MRI findings in these population of dogs and cats.

## (FP 16)

### The effect of oral zonisamide treatment on serum phenobarbital concentrations in epileptic dogs

#### E. Mahon, BVM, BVS, MRCVS^1^ , O. Marsh, BVM, BVS, MRCV ^1^, A. Uriarte, DVM, DipECVN, MRCVS^1^ , F. Stabile, DVM, PhD, DipECVN, MRCVS^1^

##### ^1^Department of Neurology and Neurosurgery, Southfields Veterinary Specialists, Basildon, United Kingdom

Zonisamide is used in dogs for the treatment of epileptic seizures as monotherapy or second‐line anti‐ epileptic medication. Zonisamide clearance is increased, and the elimination half‐life decreased, when it is used concurrently with oral phenobarbital as zonisamide is predominantly metabolized by the CYP450 hepatic enzymatic system. However, the effect that zonisamide may have on phenobarbital pharmokinetics in epileptic dogs has not been previously described. The aim of this case series is to report the effect of oral zonisamide treatment on phenobarbital pharmacokinetics in five dogs. Four dogs diagnosed with idiopathic epilepsy with tier II confidence according to the IVETF criteria and one dog diagnosed with structural epilepsy were started on oral zonisamide at 7.5–10 mg/kg/12 h following an increase in epileptic seizure frequency. All dogs were receiving oral phenobarbital at doses ranging between 3 and 6 mg/kg/12 h leading to phenobarbital serum concentrations within recommended therapeutic range (range 21.6–34.6ug/mL). Following addition of zonisamide and despite no alterations in oral phenobarbital daily dose, all dogs experienced increases in phenobarbital serum concentrations during subsequent measurements (range 27.1–45.0ug/mL). Phenobarbital serum concentrations were measured above the reported hepatotoxic concentrations (>35 ugL/mL) in 4/5 dogs. This resulted in necessary decreases in oral phenobarbital daily doses to avoid phenobarbital related hepatotoxicity. This is the first case series reporting the effect of oral zonisamide treatment on phenobarbital pharmacokinetics showing significant increases in phenobarbital serum concentrations and requiring modification of anti‐epileptic treatment management. Further studies are required to establish the pharmokinetic interactions.

## (FP 17)

### Myoclonus associated with cerebellar signs in steroid‐responsive tremor syndrome in two dogs—Electroencephalographic findings

#### L. Brewińska, DVM^1^ , A. Banasik, DVM^1^ , M. Wrzosek, DVM, PhD, dr hab., DiplECVN^1^

##### ^1^Faculty of Veterinary Medicine, Wroclaw University of Environmental and Life Sciences, Wroclaw, Poland

Myoclonus associated with cerebellar disease is described in both humans and dogs. Increased cortical excitability due to loss of cerebellar inhibitory control via cerebello‐thalamo‐cortical connections resulting in cortical myoclonus (CM) is hypothesized. CM could be detected on EEG. Myoclonus EEG features associated with cerebellar disease has not been previously reported in dog. Two female, mixed breed, middle aged dogs were presented with progressive cerebellar signs. Clinical, neurological, blood, common infectious titers, CSF, MRI and EEG examinations were performed. Neurological examination revealed cerebellar ataxia, intentional tremor, facial muscles and limbs myoclonus and opsoclonus (one case). Additional testing was unremarkable. EEG was performed under medetomidine sedation (myoclonus was persistent during examination). EEG revealed generalized delta (0.5–4 Hz) and theta (4.1–8 Hz) activity and biphasic spike‐like superimposed transients in both cases. Motion artifacts were excluded by time‐ matching standardized video. Anti‐epileptic treatment was initiated but none of the dogs responded. Suspicion of steroid‐responsive tremor syndrome has been made. Immunosuppressive treatment resulted in full resolution of clinical signs. The treatment response as well as absence of generalized seizures/consciousness alterations supports a diagnosis of non‐epileptic myoclonus. The clinical presentation, association with cerebellar symptoms as well as EEG spikes detection might reflect features of CM. However not all CM diagnostic criteria proposed for human patients has been studied. No histopathological examination has been performed to determine presence of cortical and/or cerebellar lesions.

## (FP 18)

### Antibody binding pattern in dogs with steroid‐responsive meningitis‐arteritis—Proof of principle

#### J. Nessler, Dr.^1^, A. Tipold, Prof^1^

##### ^1^Department of Small Animal Medicine and Surgery, University of Veterinary Medicine, Hannover, Germany

The etiopathogenesis of Steroid‐Responsive Meningitis‐Arteritis (SRMA) in dogs is unknown. An unidentified trigger might cause an aberrant inflammation in genetically predisposed individuals. Serum samples of five dogs with SRMA and five control dogs with cervical pain due to intervertebral disc herniation (IVDH) were examined for their different antibody binding patterns (=immunosignature) of immunoglobulin (Ig) A and IgG via customized high‐density peptide microarrays. Dogs were vaccinated against canine distemper virus (CDV) and CDV nucleoprotein peptides served as positive control. Motif discovery was performed via Multiple Expectation maximizations for Motif Elicitation (MEME). Enriched peptide sequences were compared to known protein sequences via data bank search (uniprot.com). IgG and IgA top responses exhibited moderate to very high spot intensities and were attributed to different peptides in both groups. In samples of dogs with SRMA, MEME‐analysis revealed enriched binding of IgG on peptides containing the aminoacid sequence asparagine‐asparagine‐glutamine, a sequence found in the interleukin‐1‐receptor antagonist protein (IL1Ra). In an additional, more restricted peptide microarray antibody binding patterns of IgA and IgG of dogs with IVDH and SRMA on IL1Ra were similar on one end of the protein. But dogs with SRMA showed additional binding of IgA on another part of the protein. IL1Ra is an anti‐inflammatory acute‐phase protein. Different IL1Ra isoforms might predispose individuals for autoimmune diseases. We suggest that different antibody binding patterns on IL1Ra of dogs with SRMA and IVDH might be due to different isoforms of IL1Ra which could be involved in the pathogenesis of SRMA.

## (FP 19)

### Fido's brain on fire: Anti‐gabaa receptor encephalitis in a dog

#### E. I. Hünerfauth, Dr^1^, C. G. Bien, Prof, Dr med^2^, C. Bien, Dr med^2^, H. A. Volk, Prof, Dip ECVN, PHD, PGCAP, FHEA, MRCVS^1^ , N. Meyerhoff, Dr, Dip ECVN^1^

##### ^1^Department of Small Animal Medicine and Surgery, University of Veterinary Medicine Foundation, Hannover, Germany; ^2^Laboratory Krone, Bad Salzuflen, Germany

Immune‐mediated encephalitis secondary to autoantibodies against neurotransmitter receptors is increasingly detected in people but diagnosis in dogs is currently scarce. This case describes a one‐year‐old Cavalier King Charles Spaniel with antibodies against? ‐aminobutyric acid‐A receptor (GABAAR) in cerebrospinal fluid (CSF) and serum. Presenting signs were sudden seizure onset, interictal asymmetrical neurological deficits, and severe wax and waning behavioral changes. Whereas structural MRI and standard analysis of cerebrospinal fluid (CSF) did not yield findings explaining the clinical signs, extensive analysis of the CSF and serum for neural auto‐antibodies revealed binding by antibodies to GABAAR transfected Human Embryonic Kidney cells. Seizures and abnormal behavior were unresponsive to antiseizure medication (ASM) alone but immunotherapy with dexamethasone followed by prednisolone resulted in clinical improvement. At control 4.5 weeks after start of therapy, the dog was clinically unremarkable which was reflected by a decrease of serum titer from originally 1:320 to 1:20, and in CSF from 1:2 to a normal titer. At follow‐up after 8 months, the dog showed no further seizures and was clinically normal. Corticosteroid treatment was reduced and ASM was continued. This case report describes a case of canine anti‐GABAAR encephalitis. Diagnosis was based on clinical signs, detection of serum and CSF anti‐GABAAR antibodies despite normal routine work up including structural MRI and CSF analysis. Dogs with refractory seizures, progressive behavior changes, and neurological deficits should be considered to test for neural autoantibodies. When inflammatory/infectious or neoplastic etiology could be excluded, immunotherapy is recommended and can lead to remission of clinical signs.

## (FP 20)

### Rating neurological impairment in canine meningoencephalitis: The neurodisability scale

#### R. Gonçalves, DVM MVM DipECVN FHEA MRCVS^1^ , T. Maddox, BVSc PhD DipECVDI MRCVS^1^ , S. Phillipps, BA (Hons) BVetMed (Hons) AFHEA MRCVS^1^ , A. Nagendran, BVSc DipECVN MRCVS^1^ , C. Cooper, BVSc DipECVN FHEA MRCVS^1^ , R. Orlandi, DVM AFHEA MRCVS^1^ , R. Fentem, BVSc AFHEA MRCVS^1^

##### G. Walmsley, MA VetMB PhD DipECVN MRCVS^1^

^1^Department of Veterinary Science, Small Animal Teaching Hospital, University of Liverpool, Neston, UK.

This study aimed to design and validate a disability scale that could be used in meningoencephalitis of unknown origin (MUO) to better represent clinical status. Records of 100 dogs diagnosed with MUO were retrospectively reviewed. Based on these results, a neurodisability scale (NDS) was designed and evaluated prospectively in patients diagnosed with MUO by two blinded assessors. The NDS was also evaluated retrospectively by two different blinded assessors using medical records and compared to the score recorded at the time of admission. The NDS score was calculated by attributing a numerical rating of dysfunction (0–3) in the following categories: seizures, ambulatory status, cerebral functions, cerebellar functions, brainstem functions, visual functions, and postural abnormalities. Thirty‐one consecutive patients with MUO were then prospectively examined by two independent assessors with a mean NDS score at presentation of 8 (±2.85). The interobserver intraclass correlation coefficient (ICC) for prospective use of the NDS was 0.83 (0.68–0.91) demonstrating good agreement. The interobserver ICC for retrospective use of the NDS was 0.84 (0.70–0.92) showing good agreement between the two retrospective reviewers and 0.71 (0.56–0.83) showing moderate agreement between prospective and retrospective assessors. There was no association between the NDS score and outcome at 6 months, but the score was associated with survival to discharge (*P* = 0.021) and days spent in ICU (*P* = 0.022). The NDS is a novel clinical scale for recording the degree of disability in MUO and showed good reliability. It could be used in clinical practice and may help overcome current limitations in monitoring MUO patients.

## (FP 21)

### Development of a machine learning based “web‐app” for in field identification of central nervous system infectious‐inflammatory disorders in cattle

#### S. Ferrini, DVM, MRCVS, PhD Student, ECVN Resident^1^, C. Rollo, MSc in physics, PhD Student^2^, C. Bellino, DVM, PhD^1^ , G. Boriello, DVM, PhD^1^ , G. Cagnotti, DVM, MRCVS, PhD, Dip. ECVN^1^ , C. Corona, DVM^3^ , G. Di Muro, DVM, PhD Student^1^, M. D. L. Giacobini, MSc in mathematics, PhD^1^ , B. Iulini, DVM^3^ , A. D'Angelo, DVM, PhD, Dip. ECVN, Dip. ECBHM^1^

##### ^1^Department of Veterinary Science, University of Turin, Grugliasco, Italy; ^2^Departmentof Medical Sciences, University of Turin, Turin, Italy; ^3^Istituto Zooprofilattico del Piemonte Liguria e Valle d'Aosta, Turin, Italy

Introduction: Central nervous system (CNS) infections affecting cattle are important causes of economic losses and are associated with a high rate of mortality. Machine learning (ML) techniques are becoming increasingly widespread in a large variety of predictive tasks, both in human and veterinary medicine. The objective of the present study was to develop a ML‐based clinical decision support tool for diagnoses of infectious‐inflammatory diseases in cattle presented with CNS disorders. Methods: In this study, we compared six different ML methods [Logistic regression (LR); Gradient Boosting (GB); Random Forest (RF); Multi‐layer Perceptron (MLP); Support Vector Machine (SVM); K‐nearest neighbors (KNN)] to predict the presence of infectious‐inflammatory diseases in cattle with CNS disorders, based on clinical‐ diagnostic findings and demographic data. Results: 182 animals were included. Prediction accuracies of all methods were high (± 80%). According to different metrics [accuracy (A), precision (P), recall (R) and F1 score (F1)], LR and SVM had the same best performances in predicting the diagnosis of the CNS disorders (*A* = 84%, *P* = 89%, *R* = 79%, F1 = 83%). Based on the results of the receiving operator characteristic (ROC) curve analysis [LR area under curve (AUC) 0.90; SVM AUC 0.84], LR model was chosen to be implemented in a web application. Discussion: Our findings support the use of ML algorithms as a promising tool to improve diagnosis making for veterinarians. The developed open‐access web app could help clinicians in correctly diagnose infectious‐ inflammatory neurological disorders in livestock, leading to more responsible and accurate use of antimicrobials.

## (FP 22)

### First case of presumed trigemino‐oculomotor synkinesis in a dog

#### S. Pompilio, DVM^1^ , M. Scuttari, DVM^1^ ,^2^, K. Zerbetto, DVM^1^ , M. E. Andreis, DVM, PhD^1^ , F. Tirrito, DVM, MRCVS, Dipl. ECVN^13^

##### ^1^Anicura Istituto Veterinario di Novara, Granozzo con Monticello, Novara, Italy; ^2^Neurologica, Professional Association, Torino, Italy; ^3^Studio Veterinario Associato Vet2Vet di Ferri e Porporato, Orbassano, Torino, Italy

Trigemino‐oculomotor synkinesis is an abnormal connection between the oculomotor nerve and the mandibular branch of the trigeminal nerve; as a result, the mastication activity leads to coordinated activation of the rectus muscles and simultaneous adduction and depression of the eyes. This condition has only been reported in human medicine. Our aim was to describe the case of a dog with presumed, acquired trigemino‐oculomotor synkinesis induced by a retrobulbar inflammation. An 11 year‐old, male Border Collie dog was referred for acute history of mydriasis and reduced pupillary light reflex, ventrolateral strabismus, deficit of vestibulo‐ocular reflex, and exophthalmos in the left eye. The neuroanatomical localization pointed to left oculomotor nerve with involvement of general somatic and visceral parasympathetic efferent components. Computed tomography and magnetic resonance imaging of the head were performed. Imaging findings were compatible with an inflammatory process of the left retrobulbar tissue and of the left temporalis muscle extending intracranially through the left orbital fissure. Cerebrospinal fluid analysis revealed an albuminocytological dissociation. An antibiotic therapy was started with prompt improvement of clinical signs. Four weeks later the dog showed an abnormal, bilateral adduction of the eyes and third eyelid protrusion during chewing the leash. The dog was awake and did not appear to be in discomfort during these episodes. A presumptive diagnosis of acquired trigemino‐oculomotor synkinesis induced by an intracranial inflammation was made. To the authors' knowledge this is the first case of presumed trigemino‐oculomotor synkinesis reported in veterinary medicine.

## ORAL PRESENTATIONS

## (OB 01)

### MRI and clinical findings in 133 dogs with post‐surgical recurrent neurological deficits

#### E. Suiter, BSc BVetMed PGDipVCP MRCVS^1^ , N. Grapes, BVetMed PGDipVCP MRCVS^2^ , L. Martin Garcia, DVM MRCVS^3^ , S. De Decker, DVM, PhD, DipECVN, MVetMed, PGCert Vet Ed, FHEA, MRCVS^2^ , R. Gutierrez‐Quintana, MVZ MVM DIPECVN MRCVS^3^ , A. Wessmann, DrMedVet DipECVN PGCertAcPrac FHEA MRCVS^1^

##### ^1^Pride Veterinary Centre, Derby, UK; ^2^Royal Veterinary College, London, UK; ^3^School of Veterinary Medicine University of Glasgow, Glasgow, UK

The aim of this multi‐centre retrospective study was to describe the magnetic resonance imaging (MRI) and clinical findings in dogs presenting with recurrence of clinical signs following surgical treatment of intervertebral disc herniation (IVDH). Medical records of dogs that underwent decompressive spinal surgery for IVDH followed by a second MRI due to persistent, new, or worsening neurological deficits within 12 months were reviewed. Initial neurological grade, disc location, type of surgery, and neurological signs prior to the second MRI were recorded. 133 dogs met the inclusion criteria. All dogs presented with intervertebral disc extrusion (IVDE) (118 thoracolumbar, 15 cervical) and none with a protrusion. 82.7% had a recurrent IVDE and 17.9% had an alternative diagnosis. ‘Early recurrence’ (up to 10 days post‐operatively) occurred in 30% of the cases. Of these, 64% had an IVDE at the same location (including 3 cervical IVDE) and 36% had an alternative diagnosis. Alternative diagnosis included hemorrhage (*n* = 9), infection (*n* = 4), soft tissue encroachment (*n* = 3), myelomalacia (*n* = 3) or other (*n* = 4) and were exclusively seen in the thoracolumbar cases. Dogs with hemorrhage generally initially improved before they deteriorated and were not painful. No association was seen between the diagnosis for the recurrence and the surgical technique, post‐operative progression, and neurological grade. IVDE was the most common cause for recurrence of neurological deficits following decompressive spinal surgery in our study population. One third of dogs who presented with early recurrence had an alternative diagnosis highlighting those investigations are indicated to decide on appropriate management.

## (OB 02)

### Hansen type I thoracolumbar intervertebral disc disease: Comparison between dachshunds and french bulldogs

#### T. Flegel, Dr, Dipl. ECVN, Dipl. ACVIM^1^ , S. Feiner, student^1^, L. Proske, student^1^, S. Loderstedt, Dr., Dipl. ECVN^1^ , I. C. Böttcher, Dr., Dipl. ECVN^1^ , C. Tästensen, Dr.^1^, S. Gutmann, Dr.^1^, J. Dietzel, veterinarian^1^, L. F. Becker, veterinarian^1^

##### ^1^Dept. for Small Animals, Faculty of Veterinary Medicine, Leipzig University, Leipzig, Germany

Dachshunds (DH) and French Bulldogs (FB) are the two breeds being most frequently affected by Hansen type I thoracolumbar intervertebral disc disease (TLIVDD). However, both breeds seemed to differ with regard to age at presentation, lesion localization, length of spinal cord compression, duration of hospitalization, prognosis and associated costs. Therefore, parameters mentioned above were compared between both breeds in a retrospective study including 81 DH and 97 FB. Dogs were included if the following criteria were fulfilled: clinical signs consistent with T3–S3 myelopathy, spinal imaging performed using MRI or CT‐myelography, diagnosis of Hansen type I TLIVDD between T3 and L6, surgical treatment via hemilaminectomy, follow‐up for at least 3 weeks post surgery. DH were presented at a mean age of 6.2 years and FB at a mean age of 4.0 years. The percentage of female dogs was similar in both groups with 46.4% in DH and 48.7% in FB. The mean number of intervertebral disc spaces surgically approached was 1.5 and 2.1 for DH and FB, respectively. Days of hospitalization were 5.5 for DH and 4.9 for FB. Neurological condition at discharge in comparison to the initial presentation was improved in 37.7% and 56.7%, unchanged in 53.2% and 40.0% and worsened in 9.1% and 3.3% in DH and FB respectively. Costs of the entire hospital stay were 9.9% higher in FB than in DH. FB are affected by TLIVDD at younger age, require more extensive surgery, generate higher costs, but may have a better short‐term prognosis than DH.

## (OB 03)

### Recovery of ambulation and reduction in spinal cord compression in medically managed thoracolumbar disc extrusions in non‐ambulatory small dogs

#### S. Khan, BVetMed^1^ , N. Jeffery, BVSc, PhD, MSc, CertSAO, DipECVS, DipECVN, DSAS^2^ , P. Freeman, MA, VetMB, CertSAO, DipECVN^1^

##### ^1^Department of Veterinary Medicine, University of Cambridge, Cambridge, UK; ^2^Department of Small Animal Clinical Sciences, Texas A&M University, College Station, USA

This study aimed to report the proportion of medically managed non‐ambulatory small breed dogs with thoracolumbar intervertebral disc extrusions (TL IVDE) that recover ambulation. Secondary aims were to report time to ambulation, change in severity of spinal cord compression over time, and to identify potential predictors of recovery. Dogs less than 15 kg that were non‐ambulatory because of suspected TL IVDE, and with owners unable to afford surgery, were prospectively recruited for a study of medical management. Following consultation and neurological examination, MR imaging confirmed TL IVDE and after 12 weeks of medical management dogs were re‐examined and underwent repeat MRI.50 dogs met the inclusion criteria and 3 were subsequently excluded due to having MRI findings inconsistent with IVDE. On presentation 27 were non‐ambulatory paraparetic, 7 were paraplegic with retained distal pelvic limb and tail pain perception, and 13 were paraplegic with absent hindquarter pain perception. Initial MRI showed median compression of 52% (range 17–85), including 22/47 with >50% compression. In total, 37/47 (79%) recovered ambulation including 5/13 (38%) with absent pain perception and 21/22 (95%) with >50% compression on initial MRI. The median time to ambulation was 13 days (range 1–91). Repeat MRI showed median compression of 29% (range 8–63); compression remained unchanged in some with severe initial compression that recovered ambulation.

Many non‐ambulatory small dogs with severely compressive TL IVDE spontaneously recover ambulation within 12 weeks. Spinal cord compression can greatly decrease within the same time frame, although this is not necessary for clinical recovery.

## (OB 04)

### Clinical signs, causes, and outcome of central cord syndrome in 22 cats

#### C. Ros, Ldo vet, Dipl. ECVN, MRCVS^1^ , R. José‐López, Ldo vet, Dipl. ECVN, PhD, MRCVS^2^ ^,3^, C. Font, Lda Vet, Dipl. ECVN^4^ , A. Suñol, Lda Vet, Dipl. ECVN, MRCVS^5^ , E. Alcoverro, Ldo vet, Dipl. ECVN, MRCVS^6^ , J. Nessler, DVM, Dipl. ECVN^7^ , A. Garcia de Carellan Mateo, Lda vet, Dipl. ECVAA, MRCVS^1^ , R. Gonçalves, DVM, Dipl. ECVN, PhD, MRCVS^8^

##### ^1^Memvet‐Veterinary Referral Center, Palma de Mallorca, Spain; ^2^Small Animal, School of Veterinary Medicine, University of Glasgow, Glasgow, United Kingdom; ^3^Hamilton Specialists Referrals—IVC Evidensia, High Wycombe, United Kingdom; ^4^Hospital Veterinary Canis, Girona, Spain; ^5^Hospital for Small Animals, Royal (Dick) School of Veterinary Studies, University of Edinburgh, Edinburgh, United Kingdom; ^6^Chestergates Veterinary Specialists, Telford Court, Gates Line, Chester, United Kingdom; ^7^Department of Small Animal Medicine and Surgery, University of Veterinary Medicine Hannover Foundation, Hannover, Germany; ^8^Small Animal Teaching Hospital, School of Veterinary Science, University of Liverpool, Liverpool, United Kingdom

Purpose: The aim of this study was to describe the signalment, clinical findings, presumptive or definitive aetiological diagnosis, and outcome in cats with acute and chronic central cord syndrome (CCS). Central cord syndrome is a form of tetraparesis in which the thoracic limbs are more severely affected than the pelvic limbs. Methods: Medical records and magnetic resonance imaging (MRI) studies of 22 cats presented for investigation of CCS at seven veterinary neurology specialty services were reviewed. Results: The most overrepresented breed was domestic shorthair (19/22; 86.3%) and median age was 9 years. Acute clinical signs were observed in 13/22 cats (59.1%), whereas chronic and progressive signs were seen in 9/22 (40.9%). Two neuroanatomical localisations were associated with CCS: C1–C5 spinal cord segments in 17/22 cats (77.3%), and C6‐T2 in 5/22 (22.7%). Neurolocalisation did not correlate with lesion location on MRI in 8/22 cats (36.3%). Different diseases were associated with CCS. The most common acute condition was ischaemic myelopathy, 8/22 cats (36.3%), whereas neoplasia was the most frequent chronic etiology and occurred in 5/22 cats (22.7%). Main lesion location within the vertebral column was over C2 and C4 vertebral bodies in 5/22 cats, respectively. Outcome was unsuccessful in 13/22 cats (59%), consisting of 8/9 of the chronic cases (88.8%) and 5/13 of the acute (38.4%). Conclusion: Two neuroanatomical locations were related with CCS, C1–C5 and C6–T2 spinal cord segments. Multiple aetiologies can cause CCS, most commonly ischaemic myelopathy and neoplasia. Prognosis depends on the etiology and onset of clinical signs.

## (OB 05)

### Biomechanics of the canine lumbar spine after performing multiple partial lateral corpectomies

#### L. F. Becker, Veterinarian^1^, S. Schleifenbaum, Dr.^2^, R. Heilmann, Dipl.‐Ing.^2^, E. Riemer, M. Eng.^2^, I. Kiefer, Dr.^1^, T. Siegel, Veterinarian^1^, S. Kohl, Veterinarian^1^, T. Flegel, Dr., DECVN, DACVIM (Neurology)^1^

##### ^1^Dept. for Small Animals, Faculty of Veterinary Medicine, Leipzig University, Leipzig, Germany; ^2^Center for Research on Musculoskeletal Systems, Dept. of Orthopedic Surgery, Traumatology and Plastic Surgery, Faculty of Medicine, Leipzig University, Leipzig, Germany

Different biomechanical studies provided evidence of the spine being significantly destabilized by partial lateral corpectomies (PLC). For this reason until now multiple PLC in one patient have been discouraged. To assess the biomechanical effect of two PLC in a single lumbar spine, 11 cadaveric canine lumbar spinal segments (L1‐L5) were harvested and biomechanically tested. Their native ranges of motion (ROM) in extension and flexion, lateral bending and torsion were compared to the respective ROM of the same spinal segments after having performed one and two PLC. A significant increase in ROM after introducing one and also a second PLC to the spinal segments was determined in extension and flexion and lateral bending. This increase was not linear, as the largest part of increasing the ROM was caused by the first PLC. On average, the first PLC caused 22.6% and the second PLC caused an additional 7.8% increase in ROM in extension and flexion. In lateral bending, on average, the first PLC caused 9.2% and the second PLC caused an additional 6,1% increase in ROM. The ROM in torsion was not significantly affected by any PLC. Any PLC decreases spinal stability. However spinal stability does not decrease in a linear manner with consecutive PLC and positive clinical outcome after multiple PLC is already described. Therefore, performing multiple PLC appears to be relatively safe.

## (OB 06)

### Comparison of standard T2‐W TSE versus vibe mri sequences in the assessment of caudal articular process dysplasia in pug dogs with thoracolumbar myelopathy

#### E. Gilbert, MRCVS BVetMED iBsc^1^ ,C. Driver, BSc BVetMed (Hons) MVetMed PhD DipECVN MRCVS^1^

##### ^1^Lumbry Park Veterinary Specialists, Alton, United Kingdom

The aim of the study was to retrospectively compare the accuracy of classification of caudal thoracic (T10‐T13) caudal articular process (CAP) morphology in Pug dogs with thoracolumbar myelopathy as normal, hypoplastic or aplastic, between T2 Weighted‐Turbo Spin Echo (TSE) and Volumetric Interpolated Breath‐hold Examination (VIBE) Magnetic Resonance Imaging sequences (MRI), in comparison to CT control. We hypothesized a stronger agreement for VIBE in comparison to T2W‐TSE. Images from 10 Pug dogs that underwent MRI with concurrent CT between 2020 and 2022 were included. T2W‐TSE, VIBE and CT images were independently assessed by a board‐certified neurologist and an ECVN resident, evaluating 64 anonymised CAP, classifying them as normal, hypoplastic or aplastic. Diagnostic accuracy was defined as the proportion of dogs with CAP morphology that was correctly classified using CT classification as the control group. Statistical analysis included interobserver agreement and the probability of correct classification. Diagnostic accuracy of T2W‐TSE was inferior to VIBE for aplastic (57%, 95% CI 0.45–0.67 vs. 92.5%, 95% CI 0.76–0.93) hypoplastic (32.5%, 95% CI 0.14–0.55 vs. 97.5%, 95% CI 0.79–1) and normal CAP (86.5%, 95% CI 0.74–0.97 vs, 97.5% 95% CI 0.87–1). Intra‐ and inter‐ observer agreement was inferior for T2W‐TSE (64 and 59%, 70%) versus VIBE (94 and 91%, 90%). Superior accuracy of classification using VIBE versus T2W‐TSE sequences was significant (*P* = 0.003). Three‐dimensionally reconstructable VIBE sequences were significantly more accurate than traditional T2W‐TSE MRI sequences in classifying CAP morphology, which should reduce the need for CT for pre‐operative assessment.

## (OB 07)

### Experimental assessment of imaging measurements to diagnose atlantoaxial instability on computed tomography images

#### D. Flueckiger, Med Vet^1^, B. Vidondo, Dr. Sc. Nat., M.Sc. IT^2^ , F. Forterre, Prof. Dr. Med Vet, DECVS^3^ , J. Guevar, Dr. Med Vet, DECVN MRCVS^3^ , B. Planchamp, Dr. Med Vet^3^, C. Precht, Dr. Med Vet, DECVDI^1^

##### ^1^Clinical Radiology, Department of Clinical Veterinary Medicine, Vetsuisse Faculty, University of Bern, Bern, Switzerland; ^2^Veterinary Public Health Institute, Department of Clinical Research and Veterinary Public Health, Vetsuisse Faculty, University of Bern, Bern, Switzerland; ^3^Division of Small Animal Surgery, Department of Clinical Veterinary Medicine, Vetsuisse Faculty, University of Bern, Bern, Switzerland

Introduction/Purpose: CT measurements and standardized head‐neck positions (HNP) have been proposed for the diagnosis of atlantoaxial (AA) instability in retrospective studies. Study aim: To experimentally assess the diagnostic value of imaging measurements in standardized HNP in dog cadavers before and after transection of AA ligaments. Methods: Sagittal reconstructed CT images of eight small breed dog cadavers were evaluated before and after transection of AA ligaments, whose integrity was evaluated on high‐resolution MR images and by visual inspection. The ligaments were transected via the foramen magnum, leaving other structures of the craniovertebral junction intact. Repeated measures ANOVA and ROC analysis were performed. Results: Several imaging measurements (ventral compression index [VCI], basion‐dens interval, AA‐distance, C1–C2 overlap, and cranial translation ratio) were significantly different between cadavers with intact and transected AA ligaments in extended and flexed/neutral HNP. Highest sensitivity (100%) and specificity (87.5%) is achieved using a cutoff value of =0.42 for the VCI in flexed/neutral HNP. In extended HNP, sensitivity is lower (87.5%). Discussion/Conclusion: Corresponding to previous studies, imaging measurements were significantly different between cadavers with intact and transected AA ligaments. Also, the VCI allows an objective diagnosis of AAI using a single imaging measurement. Recommendation to use a flexed/neutral position is supported, as it has a 100% sensitivity. In extended HNP, the risk to miss an AAI case is higher. Previous studies reported lower VCI cutoff values, which may be explained by less variance in the control group due to a stricter selection based on clinical and subjective imaging diagnosis.

## (OB 08)

### Dynamics of changes in dti values on the course of the spinal cord on the porcine model

#### K. Owsinska‐Schmidt, DVM, PhD Candidate^1^, A. Zimny, MD, PhD, dr hab., prof. UMED^2^ , M. Wrzosek, DVM, PhD, dr hab. prof. UPWr, diplECVN^1^

##### ^1^Department of Internal Diseases with a Clinic for Horses, Dogs and Cats, Faculty of Veterinary Medicine, Wroclaw University of Environmental and Life Sciences, Wroclaw, Poland; ^2^Department of General and Interventional Radiology and Neuroradiology, Wroclaw Medical University, Wroclaw, Poland

The aim of this study was to verify if the parameters obtained using diffusion tensor imaging (DTI) change on the course of the spinal cord. The model organism was porcine without lesion of spinal cord (19 pigs, Polish White, weight 24–120 kg, mean 48 kg, and median 48 kg). Analysis of the DTI data was performed retrospectively. DTI was performed with a 1.5 Tesla magnetic resonance scanner (Philips, Ingenia). The measured were made in three sections: cervical, thoracolumbar and lumbar segment of spinal cord, exactly between C4–C5, Th13‐L1 and L4‐L5 vertebrae and also in each case one segment cranially and one segment caudally from them. Values of fractional anisotropy (FA) and apparent diffusion coefficient (ADC) were obtained for each of the above‐mentioned places and compared. Image post‐ processing was done using the Fiber Track package (Philips Ingenia workstation), by manually drawing region of interest (9 ROIs). The collected values were subjected to statistical analysis. (TIBCO Software Inc. (2017), Statistica (data analysis software system), version 13.3.). It has been shown that FA and ADC measurement values did not significantly change between 9 ROIs on the course of the spinal cord. However, there is a noticeable tendency for FA values to decrease and ADC values to slightly increase caudally within the analyzed ROIs. Findings can be useful to determining reference values for an undamaged spinal cord and allow for a more objective assessment of the spinal cord's microstructure, both in human and veterinary medicine.

## (OB 09)

### Using AI to map mid‐sagittal neuromorphological changes in chiari‐like malformation associated pain and syringomyelia in the cavalier king charles spaniel

#### J. Cumber, MMath^1^ , C. Rusbridge, BVMS PhD DECVN FRCVS^2^ ,^3^, K. Wells, PhD^1^

##### ^1^Centre for Vision Speech and Signal Processing, Faculty of Engineering and Physical Sciences, University of Surrey, Guildford, United Kingdom; ^2^School of Veterinary Medicine, Faculty of Health and Medical Sciences, University of Surrey, Guildford, United Kingdom; ^3^Wear Referrals, Bradbury, United Kingdom

Chiari‐like malformation (CM) is a complex developmental condition resulting in neuroparenchyma/skull and craniospinal junction disproportion and is ubiquitous in Cavalier King Charles Spaniels (CKCS). A common consequence of CM is disruption to cerebrospinal fluid flow, precipitating spinal cord fluid cavitation (syringomyelia; SM). An increase in uptake of diagnostic MRI scans has resulted in an increase in the diagnoses of CM‐associated pain (CM‐P) and SM in CKCS and other brachycephalic toy breeds. This study aims to elucidate head and neck morphological changes associated with CM‐P and clinically‐relevant SM (SM‐S). Data from 130 client owned CKCS with diagnostic MRI from one referral centre was used to study the neuromorphological changes linked to CM‐Pain and SM using machine learning. The study population comprises 34 control (no clinical signs of CM‐P or SM‐S), 51 CM‐P, 35 SM‐S, and 10 CM‐P & SM‐S. Extracted T2‐weighted midsagittal images underwent an initial rigid alignment process, followed by an elastic registration technique (Demon's deformation) to yield deformation mappings for each subject. Using machine learning AI, local (9 × 9 pixel) regions were systematically examined for significant deformations that are distinct for CM‐P or SM‐S compared to unaffected dogs. Specific morphologies linked to CM‐P, and SM‐ S, were identified using Receiver Operating Characteristic curves to determine Area Under the Curve scores of 0.87 (sensitivity 89%; specificity 76%), and 0.85 (sensitivity 84%; specificity 80%) respectively representing state‐ of‐the‐art performance. Principal components analysis of these trait deformations yields further understanding of local discordant morphologies associated with CM‐P and SM‐S.

## (OB 10)

### Spontaneous vocalization in dogs presenting for neurological evaluation between 2016 and 2022: A descriptive study

#### A. Spencer‐Taylor, BVetMed^1^ , P. Freeman, DipECVN^1^ , L. Alves, DipECVN^1^ , A. Vanhaesebrouck, DipECVN^1^ , S. Khan, BVetMed^1^ , G. Harris, DipECVN^1^

##### ^1^Queen's Veterinary School Hospital, University of Cambridge, Cambridge, United Kingdom

Introduction Spontaneous vocalization has been anecdotally associated with cervical disc disease and more robustly associated with Chiari‐like malformation and syringomyelia. This study describes the signalment, localisation and diagnoses in a cohort of dogs presenting with spontaneous vocalization. Methods The records of dogs presenting to the neurology department between 2016 and 2022 with spontaneous vocalization as a presenting sign were reviewed and analyzed. Spontaneous vocalization was defined as a sudden, high‐pitched sound when moving or at rest without an obvious inciting cause. Dogs were excluded if vocalization was an isolated episode, initiated by palpation or was associated with strenuous activity. Results 59 dogs met the inclusion criteria, 44 were male, 40 were neutered and the median age at presentation was 63 months (4–348). French Bulldog (*n* = 10), crossbreed (*n* = 8), and Cavalier King Charles Spaniel (*n* = 7) were the three commonest breeds. The most prevalent localisation was C1–C5 (*n* = 17). The most frequent diagnoses were intervertebral disc herniation (*n* = 26), syringomyelia (*n* = 6), neoplasia (*n* = 5), and atlantoaxial instability (*n* = 4). Of the 33 dogs localized to the cervical spine, intervertebral disc herniation was found in 18 of them. DiscussionIn this cohort of dogs, spontaneous vocalization was most frequently associated with a localisation of the cervical spine (more specifically C1‐C5) and a diagnosis of intervertebral disc herniation. Of those dogs that localized to the cervical spine an intervertebral disc herniation was the most likely diagnosis (18/33). Incorporating this information into a clinical reasoning framework may aid in clinical decision making especially when advanced imaging is unavailable.

## (OB 11)

### Plasma mitochondrial and nuclear dna as a potential early biomarker of progressive myelomalacia in dogs: Preliminary study

#### J. M. De Frias, DVM^1^ , A. Malik, BSc, PhD^2^ , C. Thornton, BSc, PhD, FHEA^3^ , A. H. Crawford, BVM&S BSc PhD DipECVN MRCVS^1^

##### ^1^Clinical Science and Services, Royal Veterinary College, North Mymms, UK; ^2^King's College London, Hogkin Building, Great Maze Pond, London, UK; ^3^Comparative Biomedical Sciences, Royal Veterinary College, London, UK

Progressive myelomalacia (PMM) is a fatal potential sequela of severe spinal cord injury in dogs. There are no available treatments for PMM and prompt recognition is important to enable euthanasia prior to the onset of respiratory compromise. However, reaching an antemortem diagnosis requires identification of consistent clinical signs including progressive neurological deterioration, spinal pain and hyperthermia. Early biomarkers of PMM are needed to avoid reliance on clinical deterioration, with its associated adverse welfare implications. We hypothesized that mitochondrial DNA released into the circulation from damaged and necrotic neurons would provide an early indication of PM. In this prospective study, residual plasma samples from dogs with a range of spinal cord injury severities were collected at the time of presentation. Samples were collected from 8 dogs that developed PMM and 44 dogs that did not develop PMM (5 paraplegic with absent nociception, 12 paraplegic with intact nociception). Total DNA was extracted, pretreated and real time quantitative PCR performed to determine the absolute mitochondrial DNA and nuclear DNA copy numbers in each sample. Both mitochondrial DNA and nuclear DNA were elevated in plasma from dogs that developed myelomalacia compared with those that did not. Plasma mitochondrial and nuclear DNA concentration may represent an early biomarker of PMM in dogs. Future studies are needed to evaluate a larger population of dogs with spinal cord injury and to document changes in plasma DNA concentration over time.

## (OB 12)

### Spinocerebellar ataxia in the bouvier des ardennes breed is caused by the KCNJ10 C.986 T>C variant, previously reported in the belgian malinois breed

#### K. Stee, DMV, DipECVN^1^ , M. Van Poucke, PhD^2^ , M. Pumarola, DVM, PhD, DipECVP^3^ , L. Geerinckx, DVM^4^ , I. Van Soens, DVM, PhD, DipECVN^5^ ,^6^, S. Bhatti, DVM, PhD^1^ , L. Peelman, PhD, Prof^2^, I. Cornelis, DVM, PhD, DipECVN^1^

##### ^1^Dept. of Small Animals, University of Ghent, Ghent, Belgium; ^2^Dept. of Veterinary Biosciences, University of Ghent, Ghent, Belgium; ^3^Dept. of Animal Medicine and Surgery, University of Barcelona, Barcelona, Spain; ^4^Dept. of Pathobiology, Pharmacology and Zoological Medicine, University of Ghent, Ghent, Belgium; ^5^Dept. of Small Animal, University of Liège, Liège, Belgium; ^6^Evidensia Dierenziekenhuis Hart van Brabant, Waalwijk, The Netherlands

In the Belgian Malinois breed, a KCNJ10 variant is reported to cause progressive spinocerebellar ataxia (SCA). Two different phenotypes were reported: SCA with (or without) myokymia, seizures or both (SAMS) and a more severe cerebellar phenotype (SDCA1). Five related Bouvier des Ardennes puppies with spinocerebellar ataxia were examined. Clinical signs started at 6 weeks of age in 1 puppy with a severe cerebellar phenotype, and at 8‐10 weeks of age in the 4 remaining puppies with a spinocerebellar phenotype. The first puppy displayed severe intention tremors and rapidly progressive generalized hypermetric ataxia, whereas the 4 others developed a milder progressive spinocerebellar ataxia. All puppies had absent patellar reflexes and severely decreased to absent menace responses. Euthanasia due to progression to non‐ambulatory status was performed by 9 weeks of age in the puppy with the severe cerebellar phenotype, and by 7–11 months of age in the 4 remaining puppies with the spinocerebellar phenotype. Histopathology revealed a spongy cerebellar degenerative encephalopathy, as well as a focal symmetrical demyelinating myelopathy most prominent in the spinocerebellar and corticospinal tracts. Genetic analysis revealed that the KCNJ10NC_006620.3(XM_545752.6):c.986T>C (p.[Leu329Pro]) variant, previously reported to be pathogenic for SAMS and SDCA1 in the Belgian Malinois, was homozygously present in all affected dogs and segregated perfectly in an autosomal recessive manner in the affected Bouvier des Ardennes family. Inherited spinocerebellar ataxia also affects the Bouvier des Ardennes breed. It is caused by a KCNJ10 variant and can present either as SAMS or as a more severe cerebellar phenotype.

## (OB 13)

### Phenobarbital treatment in canine idiopathic epilepsy: Effects on microbiota, its functions and behavioral comorbidities

#### A. Watanangura, DVM^1^ ,^2^, S. Meller, DVM, PhD^1^ , J. S. Suchodolski, MedVet, DrVetMed, PhD, AGAF, DACVM^3^ , R. Pilla, DVM, PhD^3^ , M. R. Khattab, BVSc, MVSc, PhD^3^ , S. Loderstedt, Dr. Med Vet, DECVN^4^ , L. F. Becker, DVM^4^ , A. Bathen‐Nöthen, Dr. Med Vet, DECVN^5^ , G. Mazzuoli‐Weber, Dr. Med Vet, PhD^6^ , H. A. Volk, DECVN, PhD, PGCAP, FHEA, MRCVS^1^

##### ^1^Department of Small Animal Medicine and Surgery, University of Veterinary Medicine Hannover, Hannover, Germany; ^2^Veterinary Research and Academic Service, Faculty of Veterinary Medicine, Kasetsart University, Nakhon Pathom, Thailand; ^3^Gastrointestinal Laboratory, Department of Small Animal Clinical Sciences, Texas A&M University, Texas, United States; ^4^Department for Small Animal, Faculty of Veterinary Medicine, Leipzig University, Leipzig, German; ^5^Tierarztpraxis, Dr A. Bathen‐Nöthen, Cologne, Germany; ^6^Institute for Physiology and Cell Biology, University of Veterinary Medicine Hannover, Hannover, Germany

Phenobarbital is the first antiseizure drug of choice in canine idiopathic epilepsy (IE). However, how phenobarbital affects gastrointestinal microbiota (GM), its functions and epilepsy comorbidities, has not yet been fully investigated. The aims of this study were to evaluate the effects of phenobarbital on taxonomic and functional alterations of GM, behavioral comorbidities and identifying potential differences between drug responders and non‐responders. Twelve drug‐naïve dogs with IE (Tier II confidence level) were included in this study. Fecal samples from each dog were collected before and 90 days after starting phenobarbital treatment for metagenomics and functional analyses using DNA shotgun sequencing, Dysbiosis Index (DI) and fecal short‐chain fatty acids (SCFA) measurement. The owners were asked to complete standardized behavioral questionnaires before and after the study. At day 90, metagenomics showed a significant decrease of Clostridiales without changes in alpha and beta diversity and DI. The functional analyses found significantly increased concentrations of total SCFA, butyrate and propionate with a trend for acetate. Comparing SCFA concentrations of drug‐responsive (*n* = 7) and drug‐ resistant (*n* = 5) dogs revealed significant higher butyrate level in drug‐responsive dogs. Genes associated trehalose biosynthesis, ribosomal synthesis and gluconeogenesis were increased, whereas V‐ATPase‐related oxidative phosphorylation genes were decreased. The behavioral assessment showed improvement in stranger‐directed and non‐social fear and trainability 90 days post‐treatment. This study revealed that phenobarbital could taxonomically and functionally affect GM, including SCFA production, part of protein synthesis and carbohydrate metabolism of GM. Moreover, the elevated butyrate level could play a crucial role between phenobarbital‐responsive and ‐resistant dogs.

## (OB 14)

### Diagnosis of electrographic seizures and nonconvulsive status epilepticus in dogs and cats that underwent eeg after ceasing of clinical seizure activity: 26 cases

#### C. Taestensen, DVM^1^ , T. Flegel, Dipl. ECVN, Dipl. ACVIM (Neurology)^1^, S. Loderstedt, Dipl. ECVN^1^ , S. Gutmann, DVM^1^ , J. Dietzel, DVM^1^ , L. Becker, DVM^1^ , T. Kalliwoda, DVM^1^ , V. Weiß, DVM^1^ , H. Demeny, DVM^2^

##### ^1^Department for Small Animals, Leipzig University, Leipzig, Germany; ^2^Faculty of Veterinary Medicine, USAMV Cluj‐Napoca, Cluj‐Napoca, Romania

Aim of the retrospective study was to determine the frequency of electrographic seizures (ES) and electrographic status epilepticus (ESE) in dogs and cats with cluster seizures and possibly reduced consciousness that underwent electroencephalography (EEG) after ceasing of clinical seizure activity. Cases were presented between June 2021 and April 2022. Signalment, history, neurological exam findings, diagnostic test results and outcome were reviewed. Electrographic seizures were defined as epileptic discharges lasting = 10 s and electrographic status epilepticus as ES = 10 min. Sixteen dogs and 10 cats met the inclusion criteria. Electrographic seizures were diagnosed in four dogs (15%) and ESE was recorded in three dogs and two cats (19%). Sixteen patients (62%) showed interictal epileptic discharges. Eleven cases (42%) were presented with reduced consciousness, including four of five patients (80%) with ESE and two of four dogs with ES. Four of five patients with ESE were = 6 years old, 3 cases with ESE had structural seizures, one cat with ESE showed reactive seizures, whereas etiology of ESE was unknown in another cat. In‐hospital mortality rate was 80 % for dogs and cats with electrographic status epilepticus and 75 % for dogs with ES. Results indicate that in patients with history of cluster seizures followed by reduced state of consciousness, there is a high risk of ES or ESE. Electroencephalography is an indispensable diagnostic tool in epilepsy patients, potentially increasing the survival rate due to early detection of ES and ESE.

## (OB 15)

### CSF‐specific oligoclonal bands in dogs with idiopathic epilepsy

#### J. Föhr, Med Vet^1^,^2^, J. Prümmer, Dr. Med Vet^1^, A. Maiolini, PhD, Dipl. ECVN^1^ , E. Marti, Professor, PhD^3^ , B. Vidondo, Dr. Sc. Nat., M.Sc. IT^4^ , I. Jelcic, Dr. med.^5^, M. Ziegler, Dipl.‐Ing.(FH)^5^, A. Bathen‐Nöthen, Dipl. ECVN^6^ , A. Tipold, Professor, Dipl. ECVN^7^ , H. Volk, Professor, PhD, Dipl. ECVN^7^ , V. Stein, Professor, PhD, Dipl. ECVN^1^

##### ^1^Division of Clinical Neurology, Vetsuisse Faculty, University of Bern, Bern, Switzerland; ^2^VetTrust AG, Tierklinik Basel, Basel, Switzerland; ^3^Department of Clinical Research and Veterinary Public Health, Division of Neurological Sciences, Vetsuisse Faculty, University of Bern, Bern, Switzerland; ^4^Veterinary Public Health Institute, Department of Clinical Research and Veterinary Public Health, Vetsuisse Faculty, University of Bern, Bern, Switzerland; ^5^Neurology Clinic, University Hospital of Zurich, Zurich, Switzerland; ^6^Tierarztpraxis Dr. A. Bathen‐Nöthen, Hatzfeldstraße, Cologne, Germany; ^7^Department of Small Animal Medicine and Surgery, University of Veterinary Medicine Hannover, Hannover, Germany

Idiopathic epilepsy is the most common form of epilepsy in dogs. Despite adequate treatment, one third of patients develop drug‐resistance to conventional antiseizure drugs. An immune‐mediated etiology is suspected in a subpopulation of dogs with idiopathic epilepsy which could be a reason for drug‐resistance. Detection of cerebrospinal fluid (CSF)‐specific immunoglobulin G (IgG)‐type oligoclonal bands (OCBs) as a sign of intrathecal IgG synthesis would substantiate this suspicion and may explain the lack of response to antiseizure drugs. We hypothesize that CSF‐specific OCBs are found in a higher percentage of dogs with drug‐resistant idiopathic epilepsy. Eighty‐four dogs with idiopathic epilepsy were recruited from three referral centres and divided in two groups based on their response to treatment (drug‐responsive vs drug‐resistant) as defined by the criteria of the IVETF (International Veterinary Epilepsy Task Force). Detection of OCBs was performed by isoelectric focusing (IEF) and immunoblot. Associations of CSF‐specific OCBs with seizure type, severity and response to therapy were calculated with logistics regression models. The prevalence of CSF‐specific OCBs in dogs with idiopathic epilepsy is 15.5% (95% confidence interval 8.5%–25%). No association was found between the presence of CSF‐specific OCBs and seizure type or severity, and drug‐ resistance (odds ratio, 95% CI: 1.9, 0.57–6.35; *P*‐value: 0.2912). However, dogs with drug‐resistant idiopathic epilepsy seem to have a higher prevalence of CSF‐specific OCBs (6/28 vs. 7/56), which could indicate that intrathecal IgG synthesis may play a role in drug resistance in a subpopulation of dogs with idiopathic epilepsy.

## (OB 16)

### Tumor‐associated antigens (TAA) in canine brain tumors for use in adjunctive immunotherapy after surgery

#### W. Pownall, Dr. Med Vet^1^,^2^, L. Lanci, Med Vet^3^, A. Maiolini, Dr. Med Vet PhD^4^ , D. Guillaume, Dr. Med Vet^4^, C. Monney^2^, A. Oevermann, Dr. Med Vet PhD^2^ , F. Forterre, Prof. Dr. Med Vet^1^

##### ^1^Division of Small Animal Surgery, Department of Clinical Veterinary Sciences, Vetsuisse Faculty, University of Bern, Bern, Switzerland; ^2^Division of Neurological Sciences, DCR‐VPH, Vetsuisse Faculty, University of Bern, Bern, Switzerland; ^3^Division of Veterinary Pharmacology and Toxicology, DCR‐VPH, Vetsuisse Faculty, University of Bern, Bern, Switzerland; ^4^Division of Clinical Neurology, Vetsuisse Faculty, University of Bern, Bern, Switzerland

Brain tumor therapy remains challenging in humans and animals. Oncoimmunotherapy directs the immune system toward specific TAA to kill tumor cells and recently became one pillar of human cancer therapy, but has rarely been applied in canine patients. Our bacterial vaccine vector‐based oncoimmunotherapy platform triggers a strong cell‐mediated immune response. To direct this oncoimmunotherapy platform against brain tumors, we screened canine brain tumor population and control brain samples on a tumor microarray for four potential TAA. Immunohistochemistry followed by cell population, qualitative and quantitative staining intensity evaluation was performed with EGFR, EphA2, PDGFRA and CD44 antibodies on a paraffin‐embedded tumor microarray containing 40 oligodendrogliomas, 29 meningiomas, 23 astrocytomas, 10 gliomatosis cerebri, 4 oligoastrocytomas and 3 control brains. EGFR was diffusely expressed in 100% of gliomatosis cerebri cases (average intensity 3.7/4) and in 25%–40% of other brain tumors at lower intensity, while absent in control brains. PDGFRA was diffusely expressed in 100% of oligodendroglioma and meningioma (2.4/4 and 2.2/4 average intensity) and 70‐80% of other tumors at high intensity. PDGFRA was present focally (<20% of the cells) at lower intensity in control brains. CD44 was found in 100% and 85% of gliomatosis cerebri and oligodendroglioma respectively, and in 50%–78% of other tumors. CD44 was present focally in control brains at similar intensity than in tumor tissue. EphA2 was strongly and diffusely expressed in >50% of cells in tumor and control brain samples. EGFR, PDGFRA and CD44 are potential TAA for brain tumor therapy. Further research should focus on their antitumoral immune response induction and search for tumor specific mutations to specifically direct the vaccine against tumor cells and avoid autoimmune reactions, increasing safety.

## (OB 17)

### Resolving an old myth by new technology—The virus behind “staggering disease” in cats

#### K. Matiasek, DrMedVetHabil AM‐ECVN ^1^, F. Pfaff, DrRerNat MSc^2^ , H. Weissenböck, DrMeDVetHabil DECPHM^3^ , C. Wylezich, DrRerNat^2^ , J. Kolodziejek, Dipl.‐Ing. Dr.techn.^4^, S. Tengstrand, DVM^5^ , F. Ecke, PhD MSc^6^ , S. Nippert, DVM^7^ , P. Starcky, Dr^2^, B. Litz, Dr^2^, J. Nessler, DrMedVet DECVN^8^ , P. Wohlsein, DrMedVet DECVP^9^ , C. Baumbach, DrMedVet^10^ , L. Mundhenk, DrMedVetHabil DECVP^11^ , A. Aebischer, Dr^12^, S. Reiche, DrRerNat^12^ , P. Weidinger, MSc^4^ , K. Olofsson Sannö, DVM^13^ , C. Rohdin, PhD DECVN^14^ ^,15^, C. Weissenbacher‐Lang, DrMedVet^3^ , J. Matt, PhD^3^ ^, 16^ M. Rosati, PhD DACVP, T. Flegel, DrMedVetHabil DACVIM (Neurology) DECVN ^6, 2,7,17, 4,18^ B. Hörnfeldt, Dr, D. Höper, Dr, R. G. Ulrich, DrRerNatHabil, N. Nowotny, DrPhil ^2,5,13,2^ M. Beer, DrMedVetHabil, C. Ley, PhD DECVP, D. Rubbenstroth, PhD DrMedVetHabil DECPVS

##### ^1^Section of Clinical & Comparative Neuropathology, Institute of Veterinary Pathology, Centre for Clinical Veterinary Medicine, Ludwig‐Maximilians‐ Universitaet Muenchen, Munich, Germany; ^2^Institute of Diagnostic Virology, Friedrich‐Loeffler‐Institut, Greifswald‐Insel Riems, Germany; ^3^Institute of Pathology, University of Veterinary Medicine Vienna, Vienna, Austria; ^4^Institute of Virology, University of Veterinary Medicine Vienna, Vienna, Austria; ^5^Department of Biomedical Sciences and Veterinary Public Health, Swedish University of Agricultural Sciences, Uppsala, Sweden; ^6^Department of Wildlife, Fish and Environmental Studies, Swedish University of Agricultural Sciences, Umeå, Sweden; ^7^Institute of Novel and Emerging Infectious Diseases, Friedrich‐Loeffler‐Institut, Greifswald‐Insel Riems, Germany; ^8^Department of Small Animal Medicine and Surgery, University of Veterinary Medicine Hannover, Hannover, Germany; ^9^Department of Pathology, University of Veterinary Medicine Hannover, Hannover, Germany; ^10^State Office for Agriculture, Food Safety and Fisheries, Rostock, Germany; ^11^Institute of Veterinary Pathology, Freie Universität Berlin, Berlin, Germany; ^12^Department of Experimental Animal Facilities and Biorisk Management, Friedrich‐Loeffler‐Institut, Greifswald‐Insel Riems, Germany; ^13^Department of Pathology and Wildlife Diseases, National Veterinary Institute (SVA), Berlin, Germany; ^14^Department of Clinical Sciences, Swedish University of Agricultural Sciences, Uppsala, Sweden; ^15^Anicura, Albano Small Animal Hospital, Danderyd, Sweden; ^16^Department of Small Animal Medicine, University of Leipzig, Leipzig, Germany; ^17^German Centre for Infection Research (DZIF), Partner site Hamburg‐Lübeck‐Borstel‐Riems, Greifswald‐Insel Riems, Germany; ^18^College of Medicine, Mohammed Bin Rashid University of Medicine and Health Sciences, Dubai, United Arab Emirates

For decades, the aetiological agent behind the encephalomyelitis leading to “staggering disease” remained a bone of contention. Among proposed candidates, in particular Borna disease virus 1 (BoDV‐1) has gained attention although a natural infection has never been documented on firm grounds. Hence, tissues from staggering disease cats and those exhibiting identical brain changes were collected in a multicentre trial and investigated by metagenomic sequencing. Upon detection of possible viral sequences, the brains were investigated immunohistochemically and by in situ hybridisation (ISH) to evaluate the presence and distribution of viral antigen and RNA throughout PCR‐positive brains as compared to non‐staggering encephalitic and non‐encephalitic controls. In 28 out of 29 candidate cats from Sweden, Austria and Germany, but not in controls, nucleic acid and/or antigen of rustrela virus (RusV), an RNA virus with single‐stranded genome of positive polarity, were identified. RusV is known to infect many species, eutheria and marsupials likewise. Immunohistochemistry and in ISH documented infection of neurons throughout cerebral cortex, cerebellum, brainstem, and spinal gray matter with and without associated cytopathological changes and inflammation. This investigation proved that both historical and recent natural cases of “staggering disease” investigated herein were caused by RusV. The infection route is yet unclear even though a reservoir rodent host has been identified and may transmit the virus as a prey. The broad species range raises the possibility of RusV to cause other hitherto unknown encephalitides of similar pathotype.

## (OB 18)

### Combination of azathioprine and prednisoloneas a treatment of meningoencephalomyelitis of unknown origin in dogs: 50 cases

#### S. Diop, DVM^1^ , T. Troupel, DVM^1^ , N. Van Caenegem, DVM^1^ , V. Mayousse, DVM, ECVN Diplomate^2^, A. Jeandel, DVM, ECVN Diplomate^3^, S. Blot, DVM, PhD, ECVN Diplomate^1^

##### ^1^Ecole nationale vétérinaire d'Alfort, Univ Paris Est Créteil, INSERM, IMRB, Maisons‐Alfort, France; ^2^Centre Hospitalier Vétérinaire des Cordeliers, Meaux, France; ^3^Centre Hospitalier Vétérinaire Pommery, Reims, France

The cornerstone of treatment for meningoencephalomyelitis of unknown origin (MUO) in dogs relies on an association of corticoids with one or several immunosuppressive drugs. Azathioprine is commonly used to treat various immune‐mediated diseases in dogs, but information regarding its efficacy in MUO is limited. The aim of this retrospective study was to evaluate the efficacy and tolerability of a combination of azathioprine and prednisolone as a treatment of MUO. A retrospective medical record review was conducted. Fifty dogs with presumptive diagnosis of MUO, that received azathioprine and prednisolone between January 2011 and December 2021, were included in the study. Diagnosis was based on clinical signs, magnetic resonance imaging features and/or cerebro‐spinal fluid analysis. The overall median survival time was 1630 days (range: 30–1825). Median survival time was not statistically different for dogs that initially presented seizures or for dogs which presented relapses. Suspected adverse drug reactions to azathioprine were identified in 12 dogs (24%), and included myelosuppression in 10 dogs (83%), hepatotoxicity in 1 dog (8%), and pancreatitis in 1 dog (8%). In 10 dogs, side effects resolved with drug discontinuation or dose reduction. Two dogs (6%) died from acute hepatic necrosis (1/50) and irreversible myelosuppression (1/50). Results suggested that combination of azathioprine and prednisolone is an efficient and reasonably safe treatment option in dogs with MUO. Adverse drug reactions were reversible in most cases. As such, hematological values and hepatic parameters should be monitored closely during treatment.

## (OB 19)

### Immunohistochemical evaluation of fibrin/ fibrinogen and D‐dimers in canine granulomatous meningoencephalitis and necrotizing encephalitides

#### M. Raposo, DVM, ECVN resident^1^,^2^, C. De La Fuente, DVM, PhD, DipECVN^2^ , M. Pumarola, DVM, PhD, DipECVP^3^ , J. Rios, BSc, MSc^4^ , S. Añor, DVM, PhD, DipACVIM(Neurology), DipECVN^12^

##### ^1^Departament de Medicina i Cirurgia Animals, Facultat de Veterinària, Universitat Autònoma de Barcelona, Bellaterra, Barcelona, Spain; ^2^Fundació Hospital Clínic Veterinari, Facultad de Veterinària, Universitat Autònoma de Barcelona, Bellaterra, Barcelona, Spain; ^3^Unitat de Patologia Murina i Comparada (UPMiC) and Networking Research Center on Bioengineering, Biomaterials and Nanomedicine (CIBER‐BBN), Departament de Medicina i Cirurgia Animals, Facultat de Veterinària, Universitat Autònoma de Barcelona, Bellaterra, Barcelona, Spain; ^4^Unitat de Bioestadística, Facultat de Medicina, Universitat Autònoma de Barcelona, Bellaterra, Barcelona, Spain

Granulomatous meningoencephalitis (GME) and necrotizing encephalitides (NE) are common inflammatory diseases of the canine central nervous system. Multiple sclerosis (MS) is a similar inflammatory disease affecting humans. There is a proven relationship between the hemostatic system and the development and progression of MS. The aim of this study was to identify fibrin/fibrinogen and D‐dimer deposits, as well as the presence of intravascular thrombosis (IVT) using immunohistochemical techniques in histopathological samples from dogs with a final diagnosis of GME or NE. Sixty‐four dogs were included in the study, 32 with GME and 32 with NE. Fibrin/fibrinogen deposition was detected in all samples, but statistical differences were found in the compartment affected (*P* < 0.001): in GME deposits were mainly located in the extracellular space, and glial cells were mainly affected in NE. IVT was detected in the microvasculature of 29/64 samples with a statistically significant higher score in NE (21/29) than in GME samples (8/29) (*P* = 0.001). The fibrinolytic system seems to be activated in both diseases, especially in NE. This finding might be related to the origin of the necrotic lesions in NE, which could be chronic ischemic lesions. Further studies are needed to investigate the association between coagulation and the lesions found in GME and NE and the role of the fibrinolytic system as a potential therapeutic target.

## (OB 20)

### Challenging the therapy of muo—A pilot study evaluating the effect of a shortened whole brain radiation therapy protocol

#### R. Herzig, Med Vet^1^, F. Steffen, DECVN^1^ , C. Rohrer Bley, DECVDI (Radiation Oncology), DACVR (Radiation Oncology)^2^

##### ^1^Neurology Department, Clinic of Small Animal Surgery, Vetsuisse Faculty Zurich, University of Zurich, Zurich, Switzerland; ^2^Division of Radiation Oncology, Department for Small Animals, Vetsuisse Faculty, University of Zurich, Zurich, Switzerland

A variety of treatment options have been described for canine meningoencephalitis of unknown origin (MUO). Only a few studies focused on radiation therapy (RT), implicating its effective use including patients refractory to medical therapy alone. However, a standard RT protocol is lacking, and further research will help to evaluate the effect of different dose regimens. Ten dogs (two with focal and eight with multifocal lesions) diagnosed with MUO based an MRI and CSF findings were prospectively enrolled to evaluate the efficacy of a shortened whole brain RT protocol (5× 4 Gy). Seven dogs were diagnosed de novo and 3 with a history of relapsing, pharmacoresistant MUO. Neurological status improved in all 10 dogs during RT, with 4 of 10 returning to normal shortly after RT, and further 2 dogs within 3 months after RT. Two dogs suffering from epileptic cluster seizures became seizure‐free after RT. Three dogs died within the first 3 months after RT, one of them with a confirmed relapse. In seven dogs, follow‐up MRI and CSF examination was performed after 3‐6 months. Lesions improved in 5 dogs on follow‐up MRI, including 2 cases with complete remission and progressed in 1 dog. After control MRI, dogs were further treated with prednisolone alone (2 dogs) and second line immunosuppressive drugs for example, ciclosporin (4 dogs) and mycophenolate‐mofetil (1 dog) in conjunction. Shortened whole‐brain RT could be an effective treatment option for MUO, specifically for cases that require rapid relief of symptoms and with relapsing history.

## (OB 21)

### Plasma carnitine deficiency in five dogs with neurological disease

#### M. Foreman, MA VetMB PGCert(VM&S) MRCVS^1^ , S. Eminaga, DVM DipECVN^2^ , G. D. Shelton, DVM PhD DACVIM^3^

##### ^1^Neurology and Neurosurgery Department, Dick White Referrals, Six Mile Bottom, UK; ^2^Neurology and Neurosurgery Department, Veterinary Referral Hospital, Melbourne, Australia; ^3^Department of Pathology, University of California San Diego, La Jolla, California, USA

Primary carnitine deficiency is a well‐described metabolic disease in human medicine with varied clinical signs, attributed to an autosomal recessive disorder of carnitine transport. This study aimed to retrospectively evaluate the clinical presentation, diagnostic approach, and response to treatment of dogs presumptively diagnosed with low total and free plasma carnitine and suspected of having primary carnitine deficiency. Five dogs were included in the study, presenting with a combination of signs including episodic dystonia (1), ataxia (2), head tremors (2), exercise intolerance (3) and lethargy (4). Investigations varied from case‐to‐case, but included hematology, biochemistry, urinalysis, magnetic resonance imaging, cerebrospinal fluid analysis, infectious disease testing, genetic testing, urine metabolic screening, muscle histopathology, plasma/muscle/ urine carnitine quantification and urine organic acid quantification. Carnitine deficiency was diagnosed based on low plasma and muscle carnitine concentrations in three dogs without urinary carnitine loss, and low total and free plasma concentrations alone in two dogs. All cases were treated with oral L‐carnitine supplementation. Three cases were also supplemented with coenzyme‐Q10 and riboflavin. One dog was euthanised after 6 months due to lack of improvement. Of the surviving cases, one dog had a resolution of clinical signs following supplementation and three had an improvement in clinical signs, with follow up over 2 years. Dogs with episodic encephalopathic signs had more favorable responses than those with concurrent gait abnormalities. Carnitine deficiency should be considered as a differential in dogs with a suspicion of metabolic encephalomyopathy, or where no other cause of the signs is identified on initial investigations.

## (OB 22)

### Quantitative proteomics of cerebrospinal fluid and serum using tandem mass tags in dogs with recurrent epileptic seizures

#### R. Baka, DVM, MSc, PhD^1^ , D. Eckersall, BSc, PhD, FRCPath^2^ , A. Horvatic, Msc, PhD^3^ , A. Gelemanovic, Msc, PhD^4^ , V. Mrljak, PhD^5^ , J. Kules, Msc, PhD^5^ , M. McLaughlin, BSc, PhD^2^ , L. V. Athanasiou, DVM, PhD^6^ , H. Q. Hanh, Msc, PhD^7^ , Z. Polizopoulou, DVM, PhD, DipECVCP^1^

##### ^1^Diagnostic Laboratory, Faculty of Veterinary Medicine, School of Health Sciences, Aristotle University of Thessaloniki, Thessaloniki, Greece; ^2^Institute of Biodiversity, Animal Health & Comparative Medicine, and School of Veterinary Medicine, College of Medicine, Veterinary Medicine and Life Sciences, University of Glasgow, Glasgow, UK; ^3^Department of Chemistry and Biochemistry, Faculty of Food Technology and Biotechnology, University of Zagreb, Zagreb, Croatia; ^4^Mediterranean Institute for Life Sciences (MedILS), Split, Croatia; ^5^Laboratory of Proteomics, Internal Diseases Clinic, Faculty of Veterinary Medicine, University of Zagreb, Zagreb, Croatia; ^6^Department of Medicine, Faculty of Veterinary Medicine, University of Thessaly, Karditsa, Greece; ^7^Diagnostic Laboratory, Faculty of Veterinary Medicine, School of Health Sciences, Aristotle University of Thessaloniki, Thessaloniki, Greece

Cerebrospinal fluid (CSF) and serum are important biological materials since both can highlight potential biomarkers in canine epilepsy. This prospective study included four groups (group A: healthy dogs, groups B, C, D: dogs with epileptic seizures of different etiology). CSF and serum proteomic profiles were compared among the four groups. Samples were analyzed by a quantitative Tandem Mass Tag‐based proteomic approach using LC‐MS/MS. Identification and relative quantification were performed using Proteome Discoverer. Gene ontology terms were analyzed based on Canis lupus familiaris database. Eighteen (CSF) and 27 (serum) proteins displayed statistically significant altered levels among the four groups (*P* < 0.05). CSF MMP2 and EFEMP2 appeared down‐regulated whereas HP and APO‐A1 were up‐regulated (groups B, D). CSF CLEC3B and PEBP4 were up‐regulated whereas APO‐A1 was down‐regulated (group C). CSF IGLL1 was down‐regulated (groups B, C) and up‐regulated (group D). CSF EFEMP2 was the only protein detected among the four groups and CSF PEBP4 was significantly different among the epileptic dogs (groups B, C and D). Similar to the CSF findings, serum HP and APO‐A1 appeared up‐regulated in all groups of epileptic dogs. CSF proteome changes could reflect that MMP2, HP and APO‐A1 may contribute to a blood‐brain barrier disruption (BBB) through the brain seizure‐induced inflammatory process. Serum HP up‐regulation indicate the passage to the CSF through the disrupted BBB. CSF MMP2 change may indicate the activation of protective mechanisms within the brain. Antiepileptic medication could influence several cellular responses and alter the CSF and serum proteome composition.

## (OB 23)

### Mitochondrial fission encephalopathy: Genetic characterization of a disease affecting bullmastiffs for 40 years

#### A. Kaczmarska, DVM MRCVS^1^ , M. Christen, DVM PhD candidate^2^, H. Vandenberghe, DVM DipECVN MRCVS^3^ , J. Penderis, BVSc MVM PhD CVR DipECVN^4^ , R. José‐López, DVM DipECVN PhD MRCVS^5^ , I. Griffiths, BVMS PhD FRCVS^1^ ^,2^, V. Jagannathan, PhD, T. Leb, PhD,^2,6^ R. Gutierrez‐Quintana, MVZ MVM DipECVN MRCVS ^1,6^

##### ^1^Small Animal Hospital, School of Veterinary Medicine, College of Medical, Veterinary and Life Sciences, University of Glasgow, Glasgow, UK; ^2^Institute of Genetics, Vetsuisse Faculty, University of Bern, Bern, Switzerland; ^3^Highcroft Veterinary Referrals, Bristol, UK; ^4^Vet‐Extra Neurology, Stirling, UK; ^5^Hamilton Specialist Referrals—IVC Evidensia, High Wycombe, UK; ^6^Shared senior authorship

Hereditary cerebellar ataxia with hydrocephalus is a hereditary neurodegenerative disease affecting Bullmastiffs that was reported for the first time in 1983. Affected puppies showed progressive ataxia, behavioral abnormalities, and impaired vision, associated with symmetrical hydrocephalus and histopathological evidence of cerebellar vacuolization, gliosis, and axonal degenerations. We recently identified two new cases with a similar phenotype and hypothesized that the dogs might be affected by the same phenotype and initiated a genetic investigation. A monogenic autosomal recessive mode of inheritance was suspected. Genomic DNA was isolated from EDTA blood from the two recently identified cases and from archived formalin‐fixed paraffin‐embedded tissue samples from two affected cases closely related to the dogs reported in 1983. The genome of one of the recent cases was sequenced and the data was compared to 564 control genomes of dogs from diverse breeds. A private homozygous frameshift variant in the mitochondrial fission factor (MFF) gene was identified, XM_038574000.1:c.471_475delinsCGCTCT, that is predicted to truncate 55% of the wild type MFF protein, XP_038429928.1: p.(Glu158Alafs*14). Genotypes of the four affected and 70 unaffected Bullmastiffs showed perfect association with the disease phenotype. Human patients with pathogenic MFF variants suffer from a similar disease phenotype called ‘encephalopathy due to defective mitochondrial and peroxisomal fission 2’. To our knowledge, this is the first report of an MFF‐related disease in domestic animals, representing a potential spontaneous large animal model. We propose to name this disease mitochondrial fission encephalopathy. This finding enables genetic testing to detect carriers and avoid breeding of affected dogs.

## (OB 24)

### Brain magnetic resonance imaging in the DE50‐MD dog model of duchenne muscular dystrophy reveals regional reductions in cerebral gray matter

#### A. Crawford, BVM&S BSc PhD FHEA DipECVN MRCVS^1^ , A. G. de la Fuente, BSc PhD^2^ ,^3^, R. Piercy, MA VetMB MS PhD Dip ACVIM FRCVS^1^

##### ^1^Comparative Neuromuscular Diseases Laboratory, Department of Clinical Science and Services, Royal Veterinary College, London, United Kingdom; ^2^Institute of Health and Biomedical Sciences of Alicante (ISABIAL), Alicante, Spain; ^3^Institute of Neuroscience CSIC‐UMH, San Juan de Alicante, Spain

Duchenne muscular dystrophy (DMD), an X‐linked disease characterized by severe and progressive muscle weakness, alongside cognitive impairment and a range of neurobehavioral disorders (including autism spectrum disorder, attention deficit hyperactivity disorder and anxiety), is caused by loss of dystrophin expression in muscles and brain. Aside from musculoskeletal signs, affected boys have reduced cerebral gray matter with altered white matter ultrastructure compared to age‐matched controls. We studied the DE50‐MD canine model of DMD, which has an intron 50 splice site mutation in the dystrophin gene, absence of full length dystrophin protein (Dp427) in muscle and brain and associated neurocognitive deficits. Eight DE50‐MD and 6 age‐matched littermate wild type (WT) male dogs underwent serial brain MRI. Statistically significant reduced regional gray matter was detected in DE50‐MD dogs compared with WT, specifically in the piriform lobe, hippocampus and cingulate gyrus. Lateral ventricle volume was higher in DE50‐ MD dogs. These differences did not progress with age. White matter volume was the same in DE50‐MD and WT dogs. Dp427 deficiency in the canine brain results in structural changes that likely contribute to the neurocognitive phenotype and provide important insights into key brain regions to evaluate for relevant neurobehavioral comorbidities contributing to brain dysfunction in DMD in dogs and huma.

## (OB 25)

### Surgical and clinical findings of brachial plexus and spinal nerve tumor excision with limb preservation in dogs

#### F. Aranda, resident ECVS^1^ , I. Mateo, Dipl. ECVN^1^

##### ^1^Hospital veterinario vetsia, Leganés, Spain

Although compartmental peripheral nerve tumor resection with limb preservation has been demonstrated excellent long term results, the surgical technique and outcome of dogs undergoing excision of brachial plexus with spinal nerve tumors has not been reported. The aim of this presentation is to describe the surgical technique and long term results of neurectomy with limb preservation in five dogs with caudal brachial plexus and C8 spinal nerve tumors. An incision was made from the midpoint of the cranial border of the scapula to the greater tubercle of the humerus exposing the omotransversarius muscle which was transected over the cranial border of the scapula. Dissection was continued ventrally through the dorsolateral border of the cleidobrachialis muscle and proximally to the trapezius muscle. Both muscles were partially incised, allowing caudal elevation and retraction of the scapula. Then, the scalenus muscle was sectioned just cranial to the first rib to expose the tumored brachial plexus and C7, C8 and T1 spinal nerves. Dissection continued distally until normal nerve tissue was found and the peripheral nerves were transected. The epaxial musculature was elevated from the lateral aspect of the C7 and T1 to allow minihemilaminectomy. Dorsal and ventral nerve roots transection proximally from the ganglion allowed ventral traction for complete removal of the tumor avoiding damage to the vertebral artery and vein. Although limb function was improved in two dogs, pain was relieved in all cases. Tumor recurrence occurred locally in one dog and distal metastases occurred in two.

## (OB 26)

### Assessment of correlation between dti values, medullary kinking angle and neurological symptoms in the course of cm‐sm syndrome in Cavalier king Charles Spaniel

#### K. Owsinska‐Schmidt, DVM, PhD student^1^, A. Banasik, DVM, PhD student^1^,A. Zimny, MD, PhD, dr hab., prof. UMED^2^ , M. Wrzosek, DVM, PhD, dr hab. prof. UPWr, diplECVN^1^

##### ^1^Department of Internal Diseases with a Clinic for Horses, Dogs and Cats, Faculty of Veterinary Medicine, Wroclaw University of Environmental and Life Sciences, Wroclaw, Poland, Wroclaw, Poland; ^2^Department of General and Interventional Radiology and Neuroradiology, Wroclaw Medical University, Wroclaw, Poland, Wroclaw, Poland

This study aimed to find a correlation between diffusion tensor imaging (DTI) values, medullary kinking angle, and neurological symptoms in the course of syringomyelia secondary to Chiari‐like malformation (CM‐SM) in Cavalier King Charles Spaniel dogs (CKCS). Forty CKCS (16 in the symptomatic and 24 in the asymptomatic group), 11–90 months of age (mean 37 and median 29), 4.8–11.8 kg (mean 7.8 and median 7.6), were qualified for the research. All animals underwent the same study protocol that included a clinical and neurological examination followed by MRI. Standard MRI sequences and DTI were performed with a 1.5 Tesla magnetic resonance scanner (Philips, Ingenia). Medullary kinking angle (between the caudal margins of a medulla oblongata and the ventral plane of a spinal cord) and two DTI parameters: fractional anisotropy (FA) and apparent diffusion coefficient (ADC) were measured. Measurement of FA and ADC values was made by manually drawing the region of interest (ROI) at the craniocervical junction (CCJ). Image post‐processing was done using the Fiber Track package (Philips Ingenia workstation). The collected values were subjected to statistical analysis. Statistical analysis showed no significant differences in the medullary kinking angle between the symptomatic and asymptomatic groups. However, it can be seen that the medullary kinking is slightly greater in the symptomatic group compared to the asymptomatic group. The study confirmed statistically significant differences in FA (*P* = 0.002) and ADC (*P* = 0,040) values between the symptomatic and asymptomatic groups. FA values are lower and ADC values higher in the symptomatic group compared to the asymptomatic group. Findings suggest that at the site of CCJ, the spinal cord can be damaged at the level of its microstructure, which correlates with the development of CM‐SM symptoms.

## POSTER PRESENTATIONS

## (P 01)

### Atypical magnetic resonance imaging features of a middle ear tympanokeratoma in an american bulldog

#### K. Santifort, DVM, resident ECVN^1^ ,^2^, A. van der Lee, DVM, Dip. ECVD^1^ , K. van Amersfort, DVM, resident ECVD^1^ , S. Platt, DVM, Dip. ACVIM (neurology), Dip. ECVN^3^ , N. Corzo‐Menendez, DVM, Dip. ECVDI^3^

##### ^1^Evidensia Small Animal Hospital, Arnhem, The Netherlands; ^2^Evidensia Small Animal Hospital ‘Hart van Brabant’, Waalwijk, The Netherlands; ^3^VetOracle Teleradiology, Norfolk, United Kingdom

A middle ear tympanokeratoma (TK, misnomer ‘cholesteatoma’) is an epidermoid cyst composed of keratinizing stratified squamous epithelium and extensive keratin debris. MRI provides several advantages in the diagnosis of TK. A 4.5‐year‐old male American Bulldog was presented with chronic left‐sided vestibular signs and facial paralysis. The dog had a chronic history of otorrhea and had been treated empirically for a suspected otitis media. MRI revealed an expansile, lobulated, mixed signal intensity mass in the left tympanic bulla and horizontal ear canal. The lesion extended medially into the inner ear, the ventral bulla wall was thin/lytic and the mass invaded beyond the ventral margin of the ear canal into the surrounding tissues. The content of the bulla was mainly T2‐hyperintense and T1‐isointense. On T1‐weighted post‐contrast images, the mass showed diffuse contrast enhancement. Signs of intracranial extension of inflammation were evident. Cytology and histopathology of video‐otoscopy acquired samples confirmed a diagnosis of TK with associated bacterial otitis externa/media. Atypical MRI findings of TK in this case included: 1/ the diffuse contrast enhancement of the TK and 2/thinning and lysis of the bulla tympanica. Contrasting findings are typically reported for TK: 1/only peripheral enhancement of bulla lining and surrounding soft tissues without enhancement of bulla content and 2/thickening of the bulla wall and expansion of its dimensions. Also taking into account clinical history, signalment and all other findings, it is important to not exclude TK as a differential diagnosis in case of atypical MRI features.

## (P 02)

### Vertebral vascular canal dysplasia in cats

#### K. Santifort, DVM, MSc, Resident ECVN^1^ ,^2^, N. Bergknut, DVM, MSc, PhD, Dip. ECVN^1^ , E. Glass, DVM, MSc, Dip. ACVIM (neurology)^3^, M. Bernardini, DVM, MSc, Dip. ECVN^45^

##### ^1^Evidensia Small Animal Hospital ‘Hart van Brabant’, Waalwijk, The Netherlands; ^2^Evidensia Small Animal Hospital Arnhem, Arnhem, The Netherlands3 Red Bank Veterinary Hospital Section of Neurology and Neurosurgery, Tinton Falls (NJ), USA; ^4^AniCura Veterinary Hospital ‘Portoni Rossi’, Zola Predosa (Bologna), Italy5 University of Padua, Dept. Animal Medicine, Productions and Health, Legnaro, Italy

A vertebral anomaly termed vertebral vascular canal dysplasia (VVCD) has recently been described in French and English bulldogs. VVCD has not been reported in cats. The authors report findings of VVCD in cats. VVCD was defined as a defect in the ossification of the vertebral body of variable extent, centered around the vascular canal. A retrospective search for affected feline cases was conducted in hospital imaging databases (MRI and CT, 2018–2022). Cases with imaging findings consistent with VVCD were included. Signalment and clinical history were recorded. Imaging studies were reviewed by an ECVN resident and board‐certified neurologists. A total of 10 cats were identified; 7 domestic shorthairs (4 male neutered (MN), 3 female neutered (FN)), 1 MN British longhair, 1 MN Maine Coon and 1 MN Scottish Fold. Ages ranged from 9 months—12 years. MRI was performed in 9/10 cases, CT was performed in 1/10 cases. Radiographs were available for review in 7/10 cases. Thoracic and lumbar vertebrae were affected to a variable extent. Contents of the dysplastic vertebral vascular canal were consistent with fat in all included cases. Based on findings in this study, VVCD occurs in cats. Clinical significance of this finding may be uncovered as this anomaly is recognized in more patients, but the diagnosis of VVCD is, for instance, likely of importance in those cases where vertebral implant procedures are planned. Increased awareness of this vertebral anomaly, especially among neurologists and radiologists, could help to recognize and document future cases.

## (P 03)

### Outcome of surgical intervention of haematomyelia with a suspected traumatic cause in a pug

#### S. Guo, BSc, BVSc, Dip. ECVN, MRCVS^1^

##### ^1^CityU Veterinary Medical Centre, Hong Kong, Hong Kong

Haematomyelia is defined as intramedullary hemorrhage of the spinal cord and is a rare condition in dogs. The underlying causes that have been reported in dogs included trauma, coagulopathy, vascular malformation, vasculitis, neoplasia and primary haematomyelia where no etiology was identified. The intramedullary hemorrhage and subsequent haematoma formation may cause dysfunction of the spinal cord. Surgical intervention of the condition has been reported with variable outcome. A 10‐year‐old male neutered pug was presented with acute progressive paraplegia and urinary and fecal incontinence, after chased by three kids. There was no deep pain sensation in the right pelvic limb and the tail. Neurological examination was consistent with a painful L4‐S3 myelopathy. MRI revealed a well‐delineated intramedullary lesion over L5‐L6. The lesion was moderately heterogeneous, mainly hyperintense in T2WI, FLAIR and T1WI sequences with mild periphery contrast enhancement. Multiple areas of signal void could be identified on gradient echo images. Blood test results and buccal mucosa bleeding time were normal. A right hemilaminectomy at L5‐L6 and myelotomy were performed to remove a dark‐red friable mass. Histopathological examination identified haematoma. Post‐operatively, the dog regained ambulation in 2 weeks. Seven months after surgery, the dog could walk normally. However, he continued to have fecal and urinary incontinence. Intramedullary lesions in the spinal cord are uncommon in dogs and often associated with neoplasia. Haematomyelia should be considered as a differential diagnosis, especially when the symptoms are acute and following suspected traumatic event. Surgical decompression can result in satisfactory outcome, though neuro deficits may remain.

## (P 04)

### Investigation of neutrophil extracellular traps in dogs with steroid‐responsive meningitis‐arteritis

#### J. C. Wohlsein, Veterinaria ^1^, M. Meurer, PhD^2^ ,^3^, M. Mörgelin, Dr.^4^, J. Nessler, Dr.^1^, T. Flegel, Prof., Dr.^5^, H. C. Schenk, Dr.^6^, K. Jurina, Dr.^7^, K. Rentmeister, Dr.^8^, A. Fischer, Prof., Dr.^9^, T. Gödde, Dr.^10^, M. von Köckritz‐Blickwede, Prof., Dr., PhD^2^ ,^3^, A. Tipold, Prof., Dr.^1^

##### ^1^Department of Small Animal Medicine, University of Veterinary Medicine Hannover, Hannover, Germany2 Department of Biochemistry, University of Veterinary Medicine Hannover, Foundation, Hannover, Germany; ^3^Research Centre for Emerging Infections and Zoonoses, University of Veterinary Medicine Hannover, Foundation, Hannover, Germany; ^4^Colzyx AB, Medicon Village, Lund, Sweden5 Department of Small Animal Medicine, University of Leipzig, Leipzig, Germany6 Tierklinik Lüneburg, Lüneburg, Germany7 AniCura Tierklinik Haar, Haar, German; ^8^Tieraerztliche Praxis für Neurologie, Dettelbach, Germany9 Clinic of Small Animal Medicine, Ludwig‐Maximilians‐University Munich, Munich, Germany10 Small Animal Clinic Piding, Piding, Germany

Neutrophil extracellular traps (NET) are chromatin fibers decorated with antimicrobial enzymes serving as host defense mechanism to fight infectious diseases. Excessive formation or impaired degradation of NETs is observed in immune‐mediated diseases. Aim of the study was to investigate markers of NETs in serum and cerebrospinal fluid (CSF) samples and perform visualization of NETs in CSF and histopathology samples of dogs suffering from steroid‐responsive meningitis‐arteritis (SRMA) representing a neutrophil dominated, immune mediated disease. Serum and CSF levels of citrullinated histone H3 (H3Cit) were quantified in acute diseased dogs (*n* = 30) in comparison to control groups of healthy dogs (*n* = 6), meningioma (*n* = 4) and bacterial encephalitis (*n* = 3) with ELISA. Visualization of NETs in CSF cells and histopathological samples was performed by co‐staining of characteristic NET‐markers like DNA‐histone‐1‐complex and H3Cit or myeloperoxidase with antibody‐based techniques and detection by immunofluorescence microscopy. Immunogold‐labelling of neutrophil elastase and H3Cit was performed in CSF neutrophils of one exemplary dog for ultrastructural, intracellular visualization of NET‐formation with transmission electron microscopy. H3Cit was measurable in ranges of 0.23–56.75 ng/mL in serum and 0.29–309.51 ng/mL in CSF samples during acute SRMA. NETs were visualizable in CSF cells of dogs suffering from acute SRMA and ultrastructural evidence of NETs verifies early vesicular NET‐formation. NETs were detectable histopathologically in lesions of affected tissues of cervical meninges and arteries of two dogs with chronic, recurrent SRMA. In conclusion, the appearance of NETs in SRMA enables research on specific treatment possibilities targeting this distinct immunologic dysregulation.

## (P 05)

### Description of tomographic patterns in the presumptive diagnosis of acute non‐compressive nucleus pulposus extrusion

#### E. Hidalgo Crespo, LV^1^ , A. Farré Mariné, LV, DipECVN^1^ , A. Luján Feliu‐Pascual, LV, DipECVN^1^

##### ^1^Neurology/Neurosurgery Service of AÚNA Especialidades Veterinarias hospital, Valencia, Spain

Acute non‐compressive nucleus pulposus extrusion (ANCNPE) represents the extrusion of hydrated disc material causing spinal cord contusion without significant compression. Clinical presentation is characterized by an acute, often asymmetric, and non‐progressive myelopathy. MRI findings show focal hyperintense signal within the spinal cord immediately dorsal to the affected disc space on T2W images and narrowing of the intervertebral disc (IVD) space. The objective of this study is todescribe the myeloCT characteristics in dogs presumptively diagnosed with ANCNPE. Diagnostic images of all dogs presumptively diagnosed with ANCNPE from 2013 to 2019 that underwent a myeloCT were retrospectively reviewed. The diagnosis was based on acute, non‐progressive focal myelopathy, exclusion of other spinal pathologies by myeloCT and CSF findings, and clinical improvement with NSAIDs and physiotherapy. Dogs with <3 months follow‐up were excluded. Twenty‐two dogs were included. Three main imaging patterns (IP) were encountered: IP S (9/22 = 40.9%) was characterized by a focal spinal cord swelling immediately dorsal to a narrow IVD with (8/9 = 88,89%) or without (1/9 = 11.11%) obliteration of the subarachnoid space with no spinal cord compression; IP C (10/22 = 45.5%) included a slight elevation of the ventral subarachnoid space and mild spinal cord deviation; and, in IP T (3/22 = 13.6%) a linear intramedullary tract filled with subarachnoid contrast dorsal to the affected disc space was seen in addition to the spinal cord swelling and IVD narrowing. In conclusion, although high‐field MRI is the most sensitive imaging technique to presumptively diagnose ANCNPE, myeloCT can demonstrate myelographic patterns suggestive of its presence.

## (P 06)

### Diffuse cranial neurofibroma in a 4‐month‐old dog

#### E. Hidalgo Crespo, LV^1^ , A. Farré Mariné, LV, DipECVN^1^ , M. Pumarola, DVM, PhD, Dip ECVP^2^ , A. Luján Feliu‐Pascual, LV, MRCVS, DipECVN^1^

##### ^1^Neurology/Neurosurgery Service of AÚNA Especialidades Veterinarias hospital, Valencia, Spain; ^2^Murine and Comparative Pathology Unit. Department of Animal Medicine and Surgery, Universitat Autònoma de Barcelona, Barcelona, Spain

Diffuse neurofibroma is a rare neurofibroma subtype that affects head and neck skin and subcutaneous tissues in children and young adults. Diagnosis is based on clinical symptoms, location, and histopathology. Twelve cases have been described in dogs, none affecting the cranial nerves diffusely. A 4‐month‐old female entire Pitbull cross was referred with a two‐month history of left facial paralysis, difficulty swallowing and head tilt. The neurological exam suggested a multifocal neurolocalization (left vestibulocochlear, facial, oculomotor, and hypoglossal nerves). Computed tomography revealed a well‐defined extra‐axial lesion occupying the left nasal cavity rostrally, penetrating through the round foramen into the cranial cavity and extending along the skull base and the cranial nerves through their respective foramina causing their enlargement. Left lateral ventricle dilatation, right midline shift, and bilateral middle‐ear content were observed. Cerebrospinal fluid analysis results were unremarkable. Incisional biopsy of the distal facial nerve was compatible with a fusiform cell neoplasia. Multiple nerve tract affectation, infiltrative pattern, and the presence of axons, indicated a peripheral nerve origin (neurofibroma). Prednisone (0.5 mg/kg BID) was initiated but withdrawn due to lack of clinical response. Nine months after diagnosis, the animal remains stable. To the authors knowledge, this is the first report describing a diffuse neurofibroma in a puppy. The lack of long‐ term studies of the disease renders the prognosis uncertain, but it appears to grow slowly due to absence of neurological deterioration after nine months. Diffuse neurofibroma should be included as differential diagnose in puppies with simultaneous involvement of multiple cranial nerves.

## (P 07)

### Long‐term changes of 17th and regulatory t cells in dogs with spinal cord injury after intervertebral disc herniation

#### M. Wesolowski, Med Vet^1^, P. Can, DVM, PhD^3^ , K. Warzecha, Med Vet^1^, F. Freise, Dr.^2^, J. Neßler, Dr. Med Vet, DiplECVN^1^ , A. Tipold, Prof., DiplECVN^1^

##### ^1^Department of Small Animal Medicine and Surgery, University of Veterinary Medicine, Hannover, Germany; ^2^Department of Biometry, Epidemiology and Information Processing, University of Veterinary Medicine, Hannover, Germany; ^3^Department of Surgery, Faculty of Veterinary Medicine, University of Ankara, Ankara, Turkey

Altered Th17 and regulatory T cell (Treg) values are thought to be involved in the pathogenesis of acute intervertebral disc herniation (IVDH). To evaluate whether acute IVDH itself or potential accompanying diseases are causing the dysregulation in the Th17/Treg ratios, Th17 and Treg cells were determined in peripheral blood samples at two different time points. The first determination was performed at the acute stage of the disease and the second 14 months after decompressive surgery, at time points, when inflammatory reactions in spinal cord are not detectable anymore. Twenty‐six paretic or plegic dogs with IVDH with and without coexisting immunological disease were investigated. In addition, a healthy control group was included (*n* = 14). Quantification of T cells was performed by multicolor flow cytometry. After recovery, significantly higher levels of Th17 (*P* = 0.0265) and Tregs (*P* = 0.00025) were detected in comparison to acute IVDH. No significant influences were found by examining Th17/Treg ratios. Recovered dogs and the control group did not differ significantly. Accompanying immunological diseases (*n* = 7) resulted in lower Th17 and Treg values. A significant increase in Th17 and Treg levels was detected in dogs with paresis (*n* = 16), whereas an insignificant increase was observed in dogs with plegia (*n* = 10). These findings may suggest that altered Th17 and Treg levels are caused by specific pathophysiological responses in the acute stage of IVDH and may be considered when evaluating new treatment strategies.

## (P 08)

### Clinical, magnetic resonance imaging and surgical features of intervertebral disc extrusions in french bulldogs

#### G. Albertini, DVM, MRCVS^1^ , F. Stabile, DVM, PhD, DECVN, MRCVS, EBVS and RCVS Recognized Specialist^1^, O. Marsh, BVM, BVS, MRCVS^1^ , A. Uriarte, DVM, DECVN, MRCVS, EBVS and RCVS Recognized Specialist^1^

##### ^1^Neurology and Neurosurgery Department, Southfields Veterinary Specialists, Basildon, United Kingdom

Intervertebral disc (IVD) extrusion (IVDE) is the most commonly reported neurological condition in French bulldogs (FBD). The aim of this study was to retrospectively evaluate neurological status (5‐scale grading system), magnetic resonance imaging (MRI) and surgical findings of IVDE in FBD referred to a single institution between January 2020 and March 2022. Data was gathered from medical records and analyzed via Fischer's Exact‐Test and Kruskal Wallis‐tests. Statistical significance was assumed when *P* < 0.05. Thirty‐nine FBD were diagnosed with IVDE. Cervical‐IVDE (CIVDE) was diagnosed in 11/39 cases; the C3‐C4 IVD was the most common site (5/11). Thoracolumbar‐IVDE (TLIVDE) was diagnosed in 28 cases; the L3‐L4 IVD was the most common site (7/28). At admission, CIVDE was significantly associated with less severe neurological status (grade 1‐2) compared to TLIVDE (grade 2–5) (*P* < 0.001). The extruded IVD‐material (EIVDM) was hypointense in T2w‐images in 11/11‐CIVDE versus 2/28‐TLIVDE, and hypointense in T1w‐images in 10/11‐CIVDE versus 1/28‐TLIVDE. The EIVDM was hyperintense in T2w‐images in 0/11‐CIVDE versus 26/28‐TLIVDE and iso‐to‐hypointense in T1w‐ images in 1/11‐CIVDE versus 27/28‐TLIVDE (*P* < 0,001). The EIVDM extended over =2 IVD‐spaces in 0/11‐CIVDE versus 19/28‐TLIVDE (*P* < 0,001). 10/11‐CIVDE underwent single ventral‐slot, 1/11‐CIVDE underwent unilateral cervical‐ haemilaminectomy. 28/28‐TLIVDE underwent unilateral‐haemilaminectomy and 19/28‐TLIVDE underwent unilateral‐haemilaminectomy over =2 IVD‐spaces (*P* < 0,001). Haemorrhagic‐EIVDM was noticed intraoperatively in 1/11‐CIVDE versus 28/28‐TLIVDE (*P* < 0,001). In FBD, TLIVDE tended to cause higher grade of neurological dysfunction, tended to result in compression of neural structures over multiple IVD spaces and required more extensive surgical treatment than CIVDE.

## (P 09)

### Recurrent pyogranulomatous polymyositis and intramuscular abscesses secondary to corynebacterium species in a young beagle

#### E. Mahon, BVM, BVS, MRCVS^1^ , G. Albertini, DVM, MRCVS^1^ , O. Marsh, BVM, BVS, MRCVS^1^ , A. Staudacher, DVM, DipECVDI, MRCVS^2^ , F. Stabile, DVM, PhD, DipECVN, MRCVS^1^

##### ^1^Department of Neurology and Neurosurgery, Southfields Veterinary Specialists, Basildon, United Kingdom; ^2^Department of Imaging, Southfields Veterinary Specialists, Basildon, United Kingdom

Corynebacterium species (CSpp) are Gram‐positive bacteria reportedly causing dermatitis, otitis, endocarditis, urinary‐tract infections, cutaneous abscesses in dogs and pyogranulomatous polymyositis (PPM) and intramuscular abscesses in horses. PPM and intramuscular abscesses secondary to Cspp have not been reported in dogs. A 16‐month, male entire, beagle presented for a suspected relapse of steroid responsive meningitis‐arteritis, 12 months after diagnosis by magnetic resonance imaging (MRI) and cerebrospinal fluid analysis. Following initial improvement with prednisolone 1 mg/kg/12 h and ciclosporin 10 mg/kg/12 h, the dog developed lethargy and right pelvic limb (PL) lameness. Neurolocalisation was to the L4–S1 right‐sided spinal cord segments/associated peripheral nerves. Muscular disease could not be ruled‐out due to right PL myalgia. MRI revealed multiple, peripherally contrast enhancing, fluid‐isointense, cavitary lesions within the right and left great adductor and gracilis and right semimembranosus, internal and external obturator, superficial glutaeal and coccygeal muscles. Culture and sensitivity of muscle fine needle aspirations revealed Cspp without evidence of resistance. Clinical signs resolved on amoxicillin‐clavulanate 25 mg/kg/12 h and clindamycin 20 mg/kg/12 h, but relapse occurred after 6 weeks. Addition of marbofloxacin 2 mg/kg/24 h achieved clinical resolution. After 2 weeks the dog relapsed, and the owner elected euthanasia. Post‐mortem biopsies of the gracilis and semitendinosus muscles revealed PPM with necrosis consistent with recurring abscesses. Cspp should be considered a differential diagnosis for PPM and intramuscular abscesses in dogs. Although generalization is not possible, lack of response to appropriate antibiotic treatment in previously immunosuppressed dogs may carry a poor prognosis.

## (P 10)

### Magnetic resonance imaging findings and treatment of spinal subarachnoid diverticula in two dogs with positive cerebrospinal fluid titers for *Cryptococcus neoformans*

#### G. Bertoldi, DVM, MRCVS^1^ , A. Anson, DVM, PhD, Dip ECVDI^2^ , A. Uriarte, DVM, MRCVS, Dip ECVN^1^ ^1^ Southfields Veterinary Specialists, Basildon, Essex, United Kingdom

##### 2 Tufts University Cummings School of Veterinary Medicine, Massachusetts, USA

Cryptococcosis is a fungal infection that commonly presents as progressive meningoencephalomyelitis. Spinal subarachnoid diverticula (SAD) are fluid‐filled dilations in the subarachnoid space that can produce compressive myelopathy. SAD are presumed to be congenital or secondary to inflammation of the arachnoid membrane. Two adult female dogs were presented with progressive general proprioceptive ataxia and ambulatory tetraparesis. The MRI showed a focally T2W hyperintense, T1W/FLAIR hypointense fluid dilated spinal subarachnoid space with a characteristic tear‐drop shape consistent with a SAD, associated syringohydromyelia, and faint meningeal contrast enhancement. Case 1 also had a heterogenous T2W/FLAIR hyperintense, T1W isointense intramedullary lesion extending from the cranial aspect of C2, consistent with spinal cord edema/gliosis. CSF on both patients showed increased proteins, eosinophilic pleocytosis, and positive titers for *C. neoformans*. Both patients were treated with fluconazole (5 mg/kg/PO/BID) for 14 (case 1) and 5 months (case 2). Case 1 showed initial improvement but subsequent deterioration of the gait. At this time, a second MRI was performed and showed similar findings. Surgical decompression was attempted when serum and CSF antibody titters became negative. Case 2 was also treated with prednisolone for 5 months (0.5 mg/kg/SID) with a positive response but worsening after tapering. The MRI diagnosis of a SAD with meningeal contrast enhancement on a patient whose signalment and presentation are not typical of the breed, age, and/or spinal localisation should raise suspicion of Cryptococcosis. CSF analysis must be considered as a diagnostic tool in these patients. Surgical and medical treatment of SAD secondary to Cryptococcus might be challenging, and long‐term follow‐up is required in these cases.

## (P 11)

### An investigation of the nanomechanical and biomineral properties of the canine intervertebral disc

#### V. Rojas, MV, PhD candidate.^1^, R. Jugdaohsingh, PhD, MRC, Senior Investigative Scientist.^1^, J. Powell, PhD, Senior Lecturer, Head of Section MRC‐HNR.^1^, R. Oliver, Professor, MEng, DPhi, FREng, FIMMM.^2^, P. Freeman, MA, VetMB, Cert SAO, Dip ECVN, MRCVS^1^

##### ^1^Dept for Clinical Science and Services, Royal Veterinary College, United Kingdom; ^2^Dick White Referrals, United Kingdom

Several factors contribute to the herniation of the intervertebral disc (IVD) in dogs including mineralization and structural changes of the nucleus pulposus (NP). The purpose of this study is to describe differences between the NP of canine extruded and non‐extruded discs, using Atomic Force Microscopy (AFM) for topographical and nanomechanical assessment, and Fourier Transform Infrared spectroscopy (FTIR) for mineral composition of the disc material. Chondrodystrophic dogs diagnosed with intervertebral disc extrusion (either by MRI or CT) which underwent decompressive surgery were included. Extruded material from the vertebral canal as well as non‐extruded nucleus pulposus removed during prophylactic fenestration of the affected and adjacent non‐extruded discs was collected. Samples for AFM were smeared directly onto a microscope slide and tested using AFM in peak force mode. Samples for FTIR were freeze dried prior to the analysis. Samples were collected from 16 dogs, all chondrodystrophic breeds. Topographical images at a nanoscale were obtained from 5 samples (a mix of extruded and non‐extruded) using AFM. Extruded materials showed a surface with “hexagonal‐like” agglomerates suggestive of hydroxyapatite particles. A structure compatible with collagen fiber was found in one of the non‐extruded materials. In addition, a total of 47 FTIR spectra were processed suggesting mainly crystalline calcium (hydroxyapatite) in extruded material versus amorphous calcium phosphate in non‐extruded NP ´s. This study demonstrates that AFM can be applied to investigating nanomechanical properties of the canine intervertebral disc. Also, FTIR reveals potentially important differences in the mineral component of extruded versus non‐extruded discs in chondrodystrophic dogs.

## (P 12)

### Cervical myoclonus as a sign of cervical pain or myelopathy in dogs

#### M. Olender, DVM, Res. ECVN^1^ , C. Jérôme, DVM, Dip. ECVN^1^ , G. Laure, DVM, PhD, Dip. ECVDI^2^

##### ^1^AzurVet Veterinary Specialists Center, Neurology Unit, Saint‐Laurent‐du‐Var, France; ^2^Centre Hospitalier Vétérinaire Pommery, Reims, France

The objective of the study was to describe and try to classify cervical muscle jerks associated with cervical pain or myelopathy and evaluate their clinical and diagnostic significance. Dogs presenting cervical muscle jerks associated with cervical pain and/or myelopathy were included in the study. Detailed history recorded, full neurological and clinical examination and computed tomography (CT)‐ based diagnostic evaluation were available for review. Muscle jerks were observed by a board‐certified neurologist during clinical evaluation or registered on a video by the owner. All dogs received medical or surgical treatment. The presence of jerks was rechecked at short‐ and long‐terms. Twenty dogs entered the study, 13 of which were French bulldogs. All dogs had a diagnosis of cervical intervertebral disc extrusion (IVDE). There were no dogs with an extrusion caudal to C4‐C5 space. Cervical jerks were more common in French bulldogs than in other breeds. Jerks were all focal repetitive rhythmic contractions on lateral aspect of the neck (one or both sides). The jerk was classified as myoclonus based on its characteristic, according to human and veterinary classification of involuntary movements. Cervical jerk in is a rare sign associated with cervical pain or myelopathy that was exclusively secondary to cranial cervical intervertebral disc herniation in this study. Full recovery was observed following medical or surgical treatment of IVDE. The exact origin and classification of this involuntary movement is yet to be established.

## (P 13)

### Double adjacent ventral slots in cervical disc extrusion with epidural hemorrhage in four dogs

#### M. Olender, DVM, Res ECVN^1^ , J. Couturier, DVM, Dip ECVN^1^ , L. Couturier, DVM, Dip ECVDI^1^ , H. Brissot, DVM, Dip ECVS^1^ , L. Gatel, DVM, PhD, Dip ECVDI^2^

##### ^1^AzurVet Veterinary Specialists Center, Neurology Unit, Saint‐Laurent‐du‐Var, France; ^2^Centre Hospitalier Vétérinaire Pommery, Reims, France

The aim was to describe double ventral slots (VSs) as a treatment of acute cervical intervertebral disc extrusion (IVDE) with secondary extensive epidural hemorrhage (EH) in four dogs. Four dogs were retrospectively evaluated. All of them were male French bulldogs, presented with non‐ambulatory tetraparesis and cervical pain. The lesion was classified as IVDE with EH by a board‐certified radiologist based on post‐contrast computed tomography (CT) images and confirmed during surgery. Dogs were treated surgically by double adjacent VSs. A post‐operative CT study was performed in all dogs. The efficiency of the surgery was assessed by measurement of the amount of residual material on CT studies and the outcome was evaluated by clinical recheck. The extension of the lesion was between 1.3 and 2.8 times the length of C5 vertebral body. The localization of compression varied from ventral to dorsolateral to the spinal cord. The area of maximal spinal cord compression on transverse CT images was between 36% and 58%. The area of maximal residual compression on post‐operative CT was between 0% and 20%. All dogs recovered uneventfully after the surgery. This case series describes the first detailed report of extensive cervical IVDE with EH where a double adjacent VSs was used in the treatment of such lesions.

## (P 14)

### Thoracic to lumbar vertebral column length and length ratios in miniature dachshunds with and without thoracolumbar intervertebral disc extrusion

#### C. Fletcher, BVetMed, BScNeuroSci, DipAdvCanBhv, MRCVS^1^ , E. Ives, MA, VetMB, DipECVN, MRCVS^2^ ,F. Kajin, DVM, PhD^3^ , I. Seath, BSc(Hons)^4^, N. Grapes, BVetMed(Hons), PGDipVCP, MRCVS^1^ ,B. Lopes, DVM, GPCert(Neuro), MRCVS^2^ , A. Knebel, DVM^3^ , H. Volk, DipECVN, PhD, PGCAP, FHEA, MRCVS^3^ , S. De Decker, DVM, PhD, DipECVN, MVetMed, PGCertVetEd, FHEA, MRCVS^1^

##### ^1^Royal Veterinary College, University of London, Hatfield, United Kingom; ^2^Anderson Moores Veterinary Specialists, Winchester, United Kingdom; ^3^University of Veterinary Medicine, Hannover, Germany; ^4^Sunsong, Flackwell Heath, United Kingdom

The chondrodystrophic body type, consisting of a long back with short limbs, predisposes miniature dachshunds for thoracolumbar intervertebral disc extrusion (IVDE). The relationship between thoracolumbar IVDE and the relative lengths of the thoracic and lumbar vertebral column has not yet been evaluated. Prospective, multicentre study, including 151 miniature dachshunds with (*n* = 47) and without (*n* = 104) thoracolumbar IVDE. All dogs had their thoracic and lumbar vertebral columns measured with a tape measure. Detailed descriptions were provided to facilitate consistent measurement. A thoracic to lumbar vertebral column ratio was calculated to compensate for size differences. Thoracolumbar IVDE was confirmed by magnetic resonance imaging or computed tomography. The thoracic to lumbar vertebral column length ratio and absolute thoracic vertebral column length were significantly smaller in miniature dachshunds with IVDE compared to those without IVDE (*P* < 0.0001 for both). There were no significant differences for lumbar vertebral column length, age, gender, and neuter status between both groups. The results of this study suggest that the relative lengths of the thoracic and lumbar vertebral column segments could potentially contribute to the development of thoracolumbar IVDE in miniature dachshunds. Further studies are needed to validate measurements and evaluate the ideal thoracic to lumbar vertebral column length ratio in miniature dachshunds for reducing the risk of IVDE in this breed.

## (P 15)

### Spinal decompression and stabilization ina cat with lumbar vertebral pathological fracture and subluxation, following discospondylitis and spinal epidural empyema

#### A. Proteasa, DVM, PhD, PGCert(SAS)^1^, B. Walton, BVSc DSAS(Orth)^2^, I. Carrera, DVM, MVM, PhD, DipECVDI^3^ , L. Garosi, DVM, DipECVN^3^ , E. Alcoverro, DVM, DipECVN^1^ , M. Heyes, MA, VetMB, CertAVP(SAM), BA^1^ , A. Tauro, DVM, GPCert(Neuro), DipECVN^1^

##### ^1^ChesterGates Veterinary Specialists, Chester, United Kingdom; ^2^Movement Referrals, Neston, United Kingdom; ^3^VetOracle Teleneurology (CVS UK), CVS Limited, Norfolk, United Kingdom

A 1‐year‐old neutered male Maine Coon cat was referred because of a 1‐week history of spinal pain progressing to spastic paraplegia and absent nociception. Magnetic resonance imaging of the thoracolumbar spine showed an aggressive lytic lesion centred on L1–L2 vertebral endplates causing pathological fracture of the caudal L1 endplate and dorsal subluxation of the L1 vertebra. The endplates, associated disc and paravertebral muscles were heterogeneously hyperintense on T2W and STIR images, with marked contrast enhancement. A well‐defined, circumferentially compressing L1–L2 extradural lesion showed T2W‐hyperintensity and marked contrast‐enhancement. These findings were compatible with L1–L2 discospondylitis, spinal epidural empyema (SEE), and secondary L1 pathological vertebral fracture and subluxation. Surgical treatment involved a L1–L2 right‐sided hemilaminectomy, debridement of the SEE and spinal cord decompression. A unilateral construct was applied with two positive‐profile threaded pins into each vertebra and secured with polymethylmethacrylate. Staphylococcus aureus was isolated both from extradural material (intra‐operatively sampled) and blood culture. Initial medical therapy included intravenous broad‐spectrum antibiotics (amoxicillin‐clavulanic acid and marbofloxacin), oral antifungal treatment (itraconazole) and multimodal analgesia. Antibiotic therapy was continued with 6 weeks of oral amoxicillin‐clavulanic acid, based on susceptibility results. The outcome was excellent with a gradual improvement and complete neurological recovery at eight‐week post‐operative check. Radiographs of the thoracolumbar spine revealed good apposition and alignment of the L1–L2 vertebrae, with an intact apparatus and signs of vertebral fusion. To the authors' knowledge, surgical stabilization following discospondylitis and SEE has not been previously reported in cats.

## (P 16)

### Cross sectional survey of canine idiopathic epilepsy management in primary care in the United Kingdom

#### S. Griffin, BVetMed(hons) CertAVP SAM PgCertVPS MRCVS^1^ , F. Stabile, DVM(hons) MRCVS PhD DECVN DVM^2^ , L. De Risio, DVM (Hons), PhD, DECVN, PGCert Vet Ed, FHEA, FRCVS^34^

##### ^1^Vet4Life Teddington, Teddington, UZ; ^2^Southfields Veterinary Specialists, Basildon, UK; ^3^Linnaus Veterinary Limited, Sherley, UK4 DNottingham Trent University, Southwell, UK

Primary‐care veterinarians (PCV) in UK were invited to complete an 18‐question survey between October 2021 and January 2022 to gain insight on how PCV diagnose and treat CIE, assess treatment‐outcome, and what they perceive as challenges in the management of CIE. Responses of 3 questions were scored in comparison to IVETF guidelines. 235 PCV completed the survey. 87.8% of participants used phenobarbital as first‐line anti‐seizure drug (ASD) at a mean initial dose of 2.1 mg/kg/12 hours. When considering how closely participants' decisions aligned with IVETF guidelines on the topics of diagnosis, treatment, and treatment‐outcome assessment, responses only partially aligned to the IVETF guidelines. 53.2% of participants recommended neutering dog with CIE. 53.2% of participants did not recommend additional treatments beyond ASDs. The remainder recommended diet‐ modification/supplementation and environmental modification. Main reported challenges included: management of client's expectations and management of cluster‐seizures/ status‐epilepticus. When scoring how beneficial participants found the possibility to discuss CIE‐management with a specialist in veterinary neurology form 1 (not needed) to 7 (extremely beneficial), the mean score was 5.3. This was statistically significantly negatively correlated with number of years in clinical practice. Participants reported the need for various educational resources, including long‐term ASD‐treatment and CIE information for owners. The main limitation of this study is the relatively low response rate; hence the results may not represent the entirety of the PCV UK population. However, the results of this study provide a starting point to inform educational resources and support strategies to improve quality care of CIE in primary care.

## (P 17)

### Dietary hyperthyroidism in a dog with epileptic seizures

#### M. Foreman, MA VetMB PGCert(VM&S) MRCVS^1^ , F. Valls Sanchez, DVM DipECVIM‐CA MRCVS^1^ , E. Scarpante, DVM DipEVCN MRCVS^1^

##### ^1^Dick White Referrals, Six Mile Bottom, UK

Dietary hyperthyroidism has been reported in dogs fed a raw meat diet containing offal. Clinical signs reported in dogs include behavioral changes, polyuria, polydipsia, weight loss, lethargy and shaking. Thyrotoxicosis in humans and cats is rarely associated with seizures, suspected secondary to increased glucose and oxygen requirements, decreased seizure threshold or increased propensity to cerebral vascular accidents. Here we discuss a two‐year‐old crossbreed dog that presented with generalized tonic‐clonic seizures, that were progressive despite treatment with phenobarbitone. The dog was fed a raw‐meat diet for 4 months prior to the onset of seizures. Clinical examination showed anxiety, aggression, and tachycardia. Neurological examination was unremarkable. Hematology, biochemistry, urinalysis, bile acid stimulation testing, magnetic resonance imaging and cerebrospinal fluid analysis were unremarkable, and serologies for *Toxoplasma gondii* and *Neospora caninum* were negative. Serum thyroxine (T4) concentrations were persistently elevated on separate days, (80 nmol/L and 67.1 nmol/L, Ref. 15–50). Thyroid stimulating hormone was within reference range. Serum phenobarbitone levels were 22 mg/L. Despite an increase in phenobarbitone dosage and a recommendation to transition from the raw food diet, the dog suffered an episode of status epilepticus and died. Analysis of a selection of the diets detected total T4 in several flavors, confirming the diet as the source of exogenous T4. To the authors' knowledge, this is the first case of dietary hyperthyroidism in a dog presenting with seizures, although a causal link is not proven. Assessing T4 concentrations during the investigation of epilepsy may be valuable, especially in dogs fed a raw meat diet.

## (P 18)

### Computed tomography findings of acute subarachnoid hemorrhage in two dogs with presumptive steroid responsive meningitis‐arteritis A

#### García‐Fluixà, GV^1^ , I. Serrano‐Puerto, GV, RCVS^1^ , A. Farré‐Mariné, LV, DipECVN^1^ , A. Lujan‐Feliu‐Pascual, LV, DipECVN, MRCVS, RCVS^1^

##### ^1^AÚna Especialidades Veterinarias—IVC Evidensia, Paterna (Valencia), Spain

Steroid responsive meningitis‐arteritis (SRMA) is an immune‐mediated inflammatory disorder targeting the leptomeninges and associated vessels. Two young dogs (<12‐month‐old cross‐breed) were referred with an acute history of severe cervical pain and pyrexia. In addition, Case 1 showed progressive tetraparesis, cerebellar ataxia and nystagmus. Neuroanatomical localization was multifocal in Case 1 and cervical in Case 2. Both presented neutrophilic leukocytosis. A 16‐slides computed tomography (CT) using soft tissue algorithm revealed a well‐defined intradural‐extramedullary hyperattenuating lesion surrounding the cervical spinal cord (65–110 HU) in both cases. The spinal cord surface and the duramater homogeneously enhanced after iodinated intravenous contrast in Case 2. Case 1 showed obstructive hydrocephalus and caudal transtentorial herniation; therefore, CSF collection was not attempted. Lumbar collection in Case 2 showed xanthochromia with severe neutrophilic pleocytosis, abundant erythrocytes and a positive Pandy reaction. A presumptive diagnosis of SRMA with subarachnoid hemorrhage (SAH) was made. Both cases showed a favorable response to tapering dose of prednisone starting at 1 mg/kg BID. Case 1 worsen after 2 months and mycophenolate mofetil (10 mg/kg BID) was added. Two years (Case 1) and 2 weeks (Case 2) after diagnosis, both dogs are neurologically normal with prednisone 0,25 mg/kg EOD and mycophenolate mofetil (10 mg/kg BID) in Case 1 and prednisone only in case 2. To the authors' knowledge, this is the first report describing CT findings of presumptive SAH in dogs with SRMA. SAH should be considered a differential diagnosis in dogs with suspected SRMA and intradural‐extramedullary circumferential hyperattenuating CT lesions with homogeneous meningeal contrast enhancement.

## (P 19)

### Does transient postictal hyperammonaemia occur in dogs?

#### C. Nilsson, DVM^1^ , S. Van Meervenne, DVM^1^

##### ^1^Anicura Kalmar Animal Clinic, Kalmar, Sweden

Introduction: Hyperammonaemia is mainly associated with acute and chronic hepatic encephalopathy with portovascular abnormalities and cirrhosis as differentials. Studies in humans and cats have shown a link between transient hyperammonaemia and epileptic seizures in cases without concurrent signs of liver failure. The aim of this study was to determine how common this is in dogs. Methods: The medical records of dogs presented at a Swedish veterinary hospital between 2017 and 2021 were retrospectively reviewed to find those that were presented after or during an epileptic seizure. The medical record had to include information on at least one ammonia value taken in close proximity to a seizure. To be diagnosed with transient hyperammonaemia the dog needed to have exceeded the normal upper limit of blood ammonia concentration on initial testing (reference interval 0–95 μmol/L), and there needed to be a normalized follow‐up value within a maximum of 3 days. Results: Seventy seven dogs qualified for inclusion. Three had hyperammonaemia on initial testing (range 98–196 μmol/L), but only one dog had a follow‐up value. Initial value of this dog was 196 μmol/L which decreased within 3 days without any specific treatment for the hyperammonaemia. Further work‐up with abdominal ultrasound revealed no cause of the transient hyperammonaemia. Discussion/Conclusion: According to this retrospective study, transient hyperammonaemia may uncommonly be noted after epileptic seizures in dogs. Consequently, a differential diagnostic list in canine patients with epileptic seizures and hyperammonaemia should not only include hepatic‐related pathologies.

## (P 20)

### Detection of leptomeningeal carcinomatosis by cerebrospinal fluid in a dog with an unremarkable brain MRI

#### T. Al Kafaji, DVM, ECVN Resident^1^, C. Cantile, DVM, PhD, Associate Professor^2^, F. Tocco, DVM^1^ , A. Gallucci, DVM, PhD, ECVN Diplomate^1^

##### ^1^Centro Neurologico Veterinario “La Fenice”, Selargius (CA), Italy; ^2^Veterinary Neuropathology‐ Department of Veterinary Medical Sciences, Pisa (PI), Italy

An 11‐year‐old intact female French Bulldog dog was presented for acute onset of epileptic seizures and a 2‐week history of disorientation. On physical examination a nodular mass at the fourth mammary gland level was observed. Neurological evaluation showed obtundation and compulsive behavior. Brain magnetic resonance imaging (MRI) did not reveal any abnormalities. Cerebrospinal fluid (CSF) collected from cerebellomedullary cistern showed a marked increase of total nucleated cell count (400 cell/μL). Cytological evaluation identified the presence of a monomorphic round cell population characterized by large cell bodies, a single eccentrical located nucleus with high nuclear: cytoplasmatic ratio and marked atypia features with anisocytosis, anisokaryosis and multiple nucleoli. Leptomeningeal carcinomatosis (LC) was primarily suspected. The dog was euthanatized for worsening of clinical signs. Histopathology identified an anaplastic mammary carcinoma. Infiltration of neoplastic cells exhibiting the same morphological features was detected along leptomeninges of telencephalon and cerebellum. Cortical and subcortical parenchymal micrometastases were also present. LC is a malignant complication of solid tumor. In human medicine, CSF cytology is the gold diagnostic standard with a higher specificity in comparison to MRI exam. Ante‐mortem diagnosis obtained by identification of neoplastic cells into CSF is reported only in one dog in association with multifocal brain lesions on MRI imaging. This is the first case of LC in a dog detected by CSF evaluation without any MRI abnormalities. LC diagnosis could be underestimated, and this finding emphasizes the usefulness of CSF cytology in patients with suspected LC even in the absence of any MRI identifiable lesions.

## (P 21)

### Surgical management of cranial thoracic stenosis and concurrent kyphoscoliosis by vertebral stabilization using low‐profile 3d‐printed patient specific drill guides

#### D. Hajdu, Msc, DrMedVet, MRCVS^1^ , B. Oxley, MA VetMB, DSAS (ortho), MRCVS^2^ , C. Driver, BVetMed, BSc MVetMed, DipECVN, PhD, MRCVS^1^

##### ^1^Lumbry Park Veterinary Specialists, Alton, United Kingdom; ^2^Vet3D, Cumbria, United Kingdom

Reports regarding the surgical management of cranial thoracic stenosis in dogs are limited to decompressive procedures. The aim of the study was to describe the clinical and imaging findings, treatment and outcome for a juvenile British‐Bulldog with cranial thoracic vertebral canal stenosis and concurrent mild thoracic kyphoscoliosis. Data on clinical presentation, diagnostic imaging, surgical procedure, complications, recovery and outcome were evaluated. Marked progressive pelvic limb ataxia and spastic ambulatory para‐paresis were present for 6 weeks prior to surgery. Multiple thoracic vertebral body and dorsal arch malformations were detected by CT and MRI. These resulted in spinal cord compression due to vertebral canal stenosis at T5‐6 and T9‐10; as well as kyphoscoliosis in the same region. Surgical vertebral stabilization between T2 and T10 was performed by placing bi‐cortical 2.0 mm screws into the lamina, pedicle and vertebral bodies using a novel design of unilateral 3D‐printed patient specific drill guides, to preserve the dorsal spinous processes. Post‐operative CT confirmed adequate implant positioning. Return of independent ambulation was achieved within 6 days. Neurological status worsened 5 weeks post‐operatively, coinciding with seroma formation around the surgical site. Neurological signs had completely resolved 2 months after surgery. A follow‐up examination and CT scan 15 months post‐operative revealed normal neurological status, resolution of cranial thoracic stenosis and no further change in kyphotic and scoliotic cobb angles. This surgical technique limited several post‐operative risks including spinal cord compression through a laminectomy defect and vertebral listhesis, associated with the instability caused by loss of the spinous processes.

## (P 22)

### Spongiotic polioencephalomyelopathy (SPM) in a cat: Clinical presentation, magnetic resonance imaginG (MRI) findings and histopathology features

#### M. Moral, BSc^1^ , C. Blanco, BSc^1^ , M. Pumarola, BSc PhD^2^ , V. Lorenzo, BSc^1^

##### ^1^Neurología Veterinaria, Getafe, Madrid, Spain; ^2^Murine and Comparative Pathology Unit. School of Veterinary Science, Autonomous University of Barcelona, Barcelona, Spain

Spongy degenerations are a group of degenerative diseases characterized by a spongy state in the central nervous system (CNS) that can affect gray, white matter or both. Feline Spongiotic Polioencephalomyelopathy (SPM) has been described sporadically in Birman, Persian and Shorthair kittens affecting the gray matter. To our knowledge, this is the first case showing severe MRI changes with a final histopathologic diagnose. A 2‐year‐old, indoor spayed female, domestic shorthair cat, was referred due to progressive incoordination gait and episodic facial twitching since it was adopted 6 months ago. Neurological examination showed moderate hypermetric cerebellar ataxia and intention tremor. The neurolocalisation was intracranial‐multifocal (cerebellar predominantly) and the differential diagnose included infectious, malformative or degenerative encephalopathies. At that moment, owners declined to continue with the workup process. Eight months later, the cat was visited again because of progressive worsening and visual impairment. Blood tests were unremarkable and feline viral tests were negative. Brain MRI (1.5 T) disclosed severe intra‐axial symmetric and bilateral lesions widely distributed in the entire encephalon been the gray matter more affected; ventriculomegaly of the lateral ventricles and brain parenchyma atrophy was also seen. Cerebrospinal fluid showed mild hyperproteinorrachia. Euthanasia was ultimately elected by the owners considering cat's quality of life. Necropsy revealed diffuse vacuolisation and spongiosis restricted to the encephalic and cervical spinal gray matter, associated with severe neuronal loss and diffuse gliosis, compatible with SPM. This case contributes to scientific knowledge of neurodegenerative diseases and provides MRI description which could help clinicians in making a diagnosis.

## (P 23)

### Dandy Walker‐like malformation in an adult cat with seizures: Case description and magnetic resonance imaging characteristics

#### S. Formoso, DVM^1^ , H. Padley, BVSc^2^ , L. Alves, DVM, PhD, DipECVN^1^

##### ^1^Dept. of Small Animal Medicine, Queen's Veterinary School Hospital, University of Cambridge, Cambridge, United Kingdom; ^2^Anderson Moores Veterinary Specialists, Hursley, United Kingdom

Dandy Walker Malformation (DWM) is a congenital abnormality described in people characterized by hypoplasia of the cerebellar vermis, cystic dilation of the fourth ventricle and enlarged posterior fossa with displacement of the tentorium cerebelli and torcula, with similar abnormalities described in animals. The computed tomographic features of a kitten with DWM have been previously published. However, its magnetic resonance imaging (MRI) characteristics have not yet been reported. A 2‐year‐old male neutered domestic short hair cat was referred for investigations of a 10‐month history of self‐limiting, generalized tonic‐clonic seizures. The cat was reported to be normal interictally but had always a static abnormal gait. General physical examination was unremarkable. Neuroanatomical localization was compatible with a diffuse cerebellar and diffuse forebrain lesion. Complete blood count, biochemistry, bile acid stimulation test, urinalysis, cisternal cerebrospinal fluid (CSF) analysis, Toxoplasma Gondi serology and Toxoplasma Gondi polymerase chain reaction in CSF were all unremarkable. MRI revealed an abnormal caudal fossa, absent cerebellar vermis and small cerebellar hemispheres with distension of the fourth ventricle. There were no forebrain abnormalities identified in the MRI or CSF changes that could justify the seizures. Considering the clinical presentation, cat's neurological examination and MRI features, a presumptive diagnosis of Dandy Walker‐like syndrome and epilepsy of unknown etiology was made. This is the first case report of an adult cat diagnosed with cerebellar malformation resembling Dandy‐Walker like syndrome with concomitant seizures. Three years follow‐up consultation revealed static neurological status with two to four seizures per year. His quality of life remains good.

## (P 24)

### Clinical and magnetic resonance imaging features of *Candida albicans* invading the nervous system in a dog

#### E. Royaux, DVM, MRCVS, DipECVN^1^ , M. Czerwinska, MRCVS, CertAVP(VC)^1^,D. Corbetta, DVM, MVM, MRCVS, AFHEA Dipl ECVP EBVS^2^ , M. Genain, MRCVS, DipECVDI^1^

##### ^1^Davies Veterinary Specialists, Hitchin, UK; ^2^Cambridge University, Cambridge, UK

*Candida* spp. are pleomorphic fungi that are normal inhabitants of the oral, gastrointestinal, upper respiratory and urogenital mucosa of mammals. Opportunistic infection may develop as result of a break in the mucosal barrier, immunosuppression, and treatment with broad‐spectrum antibiotics. *Candida albicans* is the most common pathogen among the candida species. A 3‐year‐old female neutered German Shepherd Dog presented for a 3‐day history of abnormal mentation, neck pain and ataxia. Past medical history included an open pyometra treated surgical 2 months earlier and chronic gastrointestinal problems. Neurological examination revealed an abnormal mentation, right sided head turn, non‐ambulatory tetraparesis and neck pain. A multifocal lesion was suspected, and neoplastic, inflammatory and infectious conditions were considered. MRI scan of the brain revealed multiple, intra‐axial, target‐like nodular lesions scattered throughout the brain parenchyma and nodules distributed within the temporal muscles. Examination of the CSF showed a pleocytosis and increase in total protein. On cytology of the CSF, there were predominantly non‐degenerate neutrophils. Culture of the CSF was negative. Cytology of the fine needle aspirates of the nodules in the temporal muscles revealed a neutrophilic inflammation with hyalohyphomycosis. The dog was euthanized because of the poor prognosis. A post mortem examination revealed severe systemic pyogranulomatous to granulomatous inflammatory process affecting multiple organs including the brain. The changes were accompanied by severe vasculitis and intralesional fungal hyphae. Culture of brain, kidney and heart was positive for *C. albicans*. This is the first report that describes the MRI and CSF findings of a dog with neurocandidiasis.

## (P 25)

### Cerebellar hypoplasia resembling human Dandy‐Walker syndrome in a mixed breed dog

#### R. Baka, DVM, MSc, PhD^1^ , M. N. Patsikas, DVM, MD, PhD, DipECVDI^2^ , G. D. Brellou, DVM, PhD^3^ , E. Apostolopouou, DVM, PhDc^3^ , Z. S. Polizopoulou, DVM, PhD DipECVCP^1^

##### ^1^Diagnostic Laboratory, School of Veterinary Medicine, Faculty of Health Sciences, Aristotle University of Thessaloniki, Thessaloniki, Greece; ^2^Laboratory of Diagnostic Imaging, School of Veterinary Medicine, Faculty of Health Sciences, Aristotle University of Thessaloniki, Thessaloniki, Greece; ^3^Department of Pathology, School of Veterinary Medicine, Faculty of Health Sciences, Aristotle University of Thessaloniki, Thessaloniki, Greece

Cerebellar malformation resembling human Dandy–Walker syndrome had been previously diagnosed in purebred dogs in autosomal recessive inheritance mode. A 10‐month‐old, male intact, mixed‐breed dog was admitted for four‐limb progressive ataxia. Ataxia and hypermetria were first detected at the age of 2 months and worsened progressively, leading to frequent episodic falling and rolling. A generalized, tonic epileptic seizure and episodic hyperesthesia appeared the day before admission. Physical examination was unremarkable, except for hindlimb polydactyly, whereas neurological examination revealed severe ataxia, hypermetria of all limbs, posterior proprioceptive deficits, and absent menace response bilaterally. Brain MRI revealed hypoplasia of cerebellar vermis and hemispheres, dilatation of the fourth ventricle and a large‐fluid‐filled cystic structure located between the tentorium cerebelli and cerebellum. Atrophy of the cerebral white matter was noted, exhibiting high signal intensity, thalamus and quadruped tectum was inhomogeneous due to multiple low signal intensity sites on T2–weighted images. A diagnosis of cerebellar hypoplasia of congenital origin, resembling human Dandy–Walker malformation was suspected. Symptomatic therapy included anti‐inflammatory (prednisone), antiepileptic (levetiracetam) medication, along with neuropathic pain control (gabapentin). During follow‐up the dog was deteriorating progressively with non‐ambulatory ataxia, proprioceptive deficits, and cognitive dysfunction. The dog was euthanized 1 month after initially admitted. Macroscopic finding at necropsy confirmed MRI findings. Cerebellar hypoplasia resembling human Dandy–Walker syndrome should be considered in both purebred and crossbred dogs. Although polydactyly is a common finding, clinically insignificant in dogs, in humans it has been connected to Dandy–Walker syndrome.

## (P 26)

### Malformation of cortical development associated with severe clusters of epileptic seizure

#### A. Cocchetto, DVM^1^ , A. Gallucci, DVM, PhD, ECVN Dipl.^2^, F. Biggio, DVM, GPCert neurology^2^, C. Cantile, DVM, associate professor^3^

##### ^1^San Marco Veterinary Clinic and Laboratory, Neurology and Neurosurgery Division, Veggiano (PD), Italia; ^2^Veterinary Neurological Centre “La Fenice”, Selargius (CA), Italia; ^3^Veterinary Neuropathology‐ Department of Veterinary Medical Sciences, University of Pisa, Pisa (PI), Italia

In humans, malformation of cortical development (MCD) is a heterogeneous group of lesions due to neuronal migration and organization disorders, which have been recognized increasingly in patients with drug‐resistant epilepsy. Reports of epileptic seizure activity due to MCD in animals, as well as descriptions of such neural malformation, are very rare. Here we describe the neuropathological features of three cases of young animals with MCD affected with severe clusters of epileptic seizures despite the starting of antiepileptic therapy. Cases included a mixed breed dog (case 1) and a Border collie pup (case 2) with focal and diffuse cortical dysplasia, respectively, and a kitten (case 3) with lissencephaly. Gross examination at necropsy revealed morphologic alteration of the telencephalic region in case 1 and 3. Histopathologically, disorganization of cortical lamination with presence of large randomly located cytomegalic neurons was found in case 1. Altered organization of the cerebrocortical layers with abnormally distributed non‐stratified neurons, also present in the subcortical white matter, was observed in case 2. The cortical architecture of the lissencephalic cat was highly disordered with no recognizable lamination and no preservation of the molecular layer. Multifocally there were areas of neuroglial tissue extending over the cortical surface forming nodules of disorganized cortex in the leptomeningeal space. With these case series we strongly support the hypothesis that, as in humans, also in the veterinary patient, MCD could be accountable for the development of refractory epilepsy.

## (P 27)

### Investigating the effect of medium‐chain triglycerides on Th17 and regulatory T cells in dogs with idiopathic epilepsy

#### K. Warzecha, Med Vet^1^, P. Can, DVM, PhD^2^ , R. Carlson^1^, N. Meyerhoff, Dr. Med Vet, DiplECVN^1^ , F. Twele, PhD^1^ , H. A. Volk, Prof., DiplECVN^1^ , J. Neßler, Dr. Med Vet, DiplECVN^1^ , A. Tipold, Prof., DiplECVN^1^

##### ^1^Department of Small Animal Medicine and Surgery, University of Veterinary Medicine, Hannover, Germany; ^2^Department of Surgery, Faculty of Veterinary Medicine, University of Ankara, Ankara, Turkey

Increased levels of Th17 cells and a Th17/Treg cell imbalance have been suggested to influence seizure severity in a subset of dogs with idiopathic epilepsy (IE). Diets enriched with medium‐chain triglycerides (MCT) are used to treat epilepsy in dogs and humans. In a subset of healthy dogs being fed a MCT enriched diet increased Treg cell levels have been detected. The aim of our study was to determine whether oral supplementation of MCT has an effect on these cells in patients with IE. Th17 and Treg cells were measured in peripheral blood of 9 dogs with IE and 7 clinically healthy Beagles (control group) using multicolor flow cytometry. After the first blood sampling, oral supplementation of MCT was added to the treatment plan. The absolute number of cells was controlled at least once 1–10 months after the first measurement. Compared to the healthy control group, there were no statistically significant differences in Th17 and Treg cell levels and Th17/Treg ratio in dogs with IE after adding MCT. Similar to findings in healthy dogs, elevated Treg cell levels were detected in single dogs with IE (*n* = 4/9) after adding oral supplementation of MCT to the treatment plan. These findings suggest an effect of MCT on Treg cells in individual dogs potentially leading to immune system regulation in cases with epilepsy.

## (P 28)

### Stability of compounded levetiracetam solution over time

#### K. Foss, DVM, MS, DACVIM (Neurology)^1^, J. Wagner, DVM^1^ , F. Lauren, PharmD, DICVP, FSHVP^1^ , R. Jennifer, DVM, PhD, DACVIM (SAIM), DACVCP^1^

##### ^1^Department of Veterinary Medicine, College of Veterinary Medicine, University of Illinois at Urbana, Champaign, Urbana, Illinois, United States

Introduction: Levetiracetam is an antiepileptic drug commonly used in dogs. Oral and intravenous routes are employed routinely but other routes of are of interest, particularly for acute administration when intravenous or oral access is not possible. These include including rectal and intranasal routes. Intranasal administration of medications is inherently more attractive compared to rectal administration as it requires little training, is easily performed, and carries little risk of injury. The commercially available injectable product with a concentration of 100 mg/mL requires relatively large volumes of medication to be administered. This volume is easily accommodated when given intravenously over several minutes but may complicate administration by other routes given possible large volumes required in larger dogs. Purpose: The purpose of this study was to evaluate the stability of a compounded levetiracetam solution intended for intranasal administration. The formulation was prepared by dissolving levetiracetam powder in bacteriostatic saline through continuous stirring to produce a solution with a final concentration of 460 mg/mL. The stability of the compounded levetiracetam solution was evaluated for up to 60 days using a stability‐indicating assay through a commercial lab. Results: Stability testing showed that the compounded product can be stored in a plastic amber bottle at room temperature for up to 60 days while continuing to meet United States Pharmacopeia requirements for a non‐ sterile formulation. Conclusion: This stability study suggests that higher concentrated, compounded levetiracetam can be used for research or clinical use in animals, particularly for administration outside of intravenous and oral routes.

## (P 29)

### Intrathecal chemotherapy for the management of lymphoblastic lymphoma in a 4‐year‐old dog

#### F. Lyseight, MVSc MRCVS^1^ , C. Dupont, DVM MRCVS^1^ , S. Verganti, Dip ECVIM‐CA (Onc) MSc (Clin Onc) MRCVS^1^ , G. Cherubini B, DVM DipECVN FRCVS^1^

##### ^1^Dick White Referrals, Cambridge, England

In veterinary medicine the clinical benefit of intrathecal chemotherapy for the treatment of central nervous system (CNS) neoplasia remains to be defined, unlike to human medicine. A 4‐year‐old, male entire, cross breed dog, presented with a 24‐h history of severe lethargy, pelvic limb weakness and urinary retention. Examination revealed generalized peripheral lymphadenomegaly and neurological findings were compatible with T3–L3 myelopathy. Investigations (hematology, biochemistry, flow cytometry, thoracic radiographs, and abdominal ultrasound) were consistent with a multicentric lymphoblastic B‐cell lymphoma (stage Vb) with suspected CNS involvement. Complete clinical remission was achieved with systemic chemotherapy (modified L‐LOP with cytosine arabinoside). After 4 weeks there was acute neurological deterioration (spinal pain and proprioceptive deficits) without lymphadenomegaly. MRI findings and CSF analysis (nucleated cell count 1900/uL, RI: <5, predominated by atypical, large, lymphoid cells; protein 0.34 g/L, RI: 0.14–0.30), were consistent with meningeal and spinal cord lymphoma infiltration at the level of L3. Intrathecal chemotherapy (100 mg cytosine arabinoside and 2.5 mg methotrexate) was administered in the cisterna magna, with systemic dexamethasone (0.2 mg/kg q24h PO) and analgesia. Within 24‐h the neurological signs had resolved. The patient remained asymptomatic for 3.5 weeks after which relapse was suspected (proprioceptive deficits and severe thoracolumbar pain). The owners declined repetition of intrathecal chemotherapy and the patient was euthanised 9 weeks after diagnosis. Intrathecal chemotherapy was well tolerated and provided a rapid improvement of the clinical signs, although the response was short‐lasting. As in humans, repeated administrations may be required to improve remission status, further studies are required.

## (P 30)

### Vertebral giant cell tumor of bone in a domestic shorthair cat

#### J. Caldero Carrete, DVM, MRCVS^1^ , C. Rusbridge, BVMS, DipECVN, PhD, MRCVS^1^ ,^2^, J. Tabanez, DVM, MRCVS, Neurology Registrar^1^, A. Civello, BSc (Hons), BVSc, MVetMed, MRCVS, FRCPath, DipACVP^3^

##### ^1^Fitzpatrick Referrals Orthopedics and Neurology, Eashing, UK; ^2^School of Veterinary Medicine, Faculty of Health and Medical Sciences, University of Surrey, Guildford, UK; ^3^SYNLAB's Veterinary Pathology Group (VPG), Exeter, UK

Giant cell tumors of bone (GCTBs) are rare in veterinary patients and usually originate in the epiphysis of long bones. Surgery is the treatment of choice in human medicine. A 10‐year‐old male neutered domestic shorthair cat was presented with a 5‐month history of progressive non‐ ambulatory paraparesis. Initial vertebral column radiographs revealed a bony expansile lytic lesion of the right L2–L3 articular facet joint. Neurological examination revealed non‐ambulatory paraparesis with proprioceptive deficits, normal withdrawal and hyperactive patellar reflexes in both pelvic limbs. Lumbar hyperesthesia was detected. Neurological findings were consistent with a T3–L3 myelopathy. MRI of the spine revealed a well‐demarcated, compressive, extradural mass lesion affecting the caudal lamina, caudal articular processes, and right pedicle of the L2 vertebra. The mass was hypointense/isointense on T2WI, isointense on T1WI and mildly contrast enhancing. No additional neoplastic foci were found. The lesion was removed by en bloc resection via a dorsal L2‐L3 laminectomy. Vertebral stabilization was performed with titanium screws and polymethylmethacrylate (PMMA) cement. Histopathology revealed a neoplasm composed of spindle cells and multinucleated giant cells, demonstrating a low mitotic count and absence of cellular atypia. A GCTB was diagnosed. Postoperative full‐body CT at 6 months showed absence of local recurrence or metastasis. There were signs of instability of the stabilization construct demonstrated by multifocal PMMA fracturing and L2 implant loosening. The cat returned to normal activity and was ambulatory with subtle paraparesis. This is the first report describing the imaging findings, surgical treatment, histopathology, and outcome of a vertebral GCTB in a cat.

## (P 31)

### Serial magnetic resonance imaging and medical management of an intracranial abscess in a dog

#### A. Manzanedo‐Delgado, MRCVS^1^ , M. Benito‐Benito, MRCVS^1^ , J. McConnell, BVM&S, Cert SAM, DVR, DipECVDI, FRCVS^2^ , D. Sanchez‐Masian, Lic. Vet, MRCVS, DipECVN^1^

##### ^1^Hamilton Specialist Referrals, Dept of Neurology and Neurosurgery, High Wycombe, UK; ^2^Department of Veterinary Science, Small Animal Teaching Hospital, University of Liverpool, Cheshire, UK

An 11 year‐old, neutered female, Chihuahua was admitted with an acute, progressive history of behavioral changes and gait abnormalities over the last 10 days. Neurological examination showed obtunded mentation with a generalized proprioceptive ataxia and left hind limb postural reaction deficits. Magnetic Resonance Imaging (MRI) of the head showed a solitary, irregular shaped, intra‐axial mass lesion within the right temporo‐occipital lobes. The mass was located within the white matter, with extensive perilesional oedema/T2 hyperintensity. The mass had a thick, but uniform peripheral capsule/rim, which on the T2w images appeared to show a “dual rim” sign with mid‐low signal outer layer and higher signal inner layer. The center of the lesion was hyperintense on T2w, with no suppression on FLAIR images and was mildly hypointense on T1w images with no contrast enhancement. The capsule/periphery of the lesion showed dense, homogenous contrast enhancement. A large mass effect was associated with the lesion causing caudal transtentorial herniation and mild‐line shift with compression of the adjacent parenchyma. Based on the clinical and MRI findings a brain abscess was suspected. Treatment with an anti‐inflammatory dose of prednisolone for 5 days in combination with clindamycin and marbofloxacin was implemented with a remarkable improvement of the clinical signs. Two subsequent MRI studies at 1‐ and 4‐months post treatment were performed to monitor the progression of the lesion, which showed a complete resolution of the lesion in the last MRI study. The present report shows serial MRI studies of a brain abscess successfully treated with conservative management.

## (P 32)

### Pedicle screw fixation of the thoracolumbar spine in dogs

#### D. Mauler, DVM, CCRP, DECVN^1^ , R. Bergman, DVM, MS, DACVIM (Neurology)^1^, M. Dantio, DVM, DACVIM (Neurology)^1^, K. Bray, DVM, DACVIM (Neurology)^2^, R. Quigley, DVM, DACVIM (Neurology)^1^, S. Rutherford, BVMS, DECVS, MRCVS^3^ , G. Kaiman, VMD, DACVIM (Neurology)^4^, P. Early, DVM, DACVIM (Neurology)^5^

##### ^1^Carolina Veterinary Specialists, Matthews, NC, USA; ^2^Carolina Veterinary Specialists, Winston‐Salem, NC, USA; ^3^Frank Pet Surgeons, Leeds, UK; ^4^Veterinary Referral Associates, Gaithersburg, MD, USA; ^5^Neurology Service, College of Veterinary Medicine, NC State University, Raleigh, NC, USA

Pedicle screw and rod fixation (PSF) has been reported in treating lumbosacral instability in dogs. This study aimed to describe the utilization of a PSF system (Ivetra Neuromed) in the thoracolumbar spine of dogs. Twelve surgeries were performed between 2020 and 2022. Indications for surgery were vertebral fractures/luxations and a possible iatrogenic instability following bilateral hemilaminectomies. Eleven client‐owned dogs were included in this study. One dog underwent two surgeries for two fractures. Pre‐operative computed tomographic (CT) images were acquired for surgical planning, identifying the vertebrae, and measuring the width, length, and proposed angle of insertion for the pedicle screws (PS). Pedicle screws were predominantly placed in the vertebral body and occasionally vertebral pedicle. The PS were then connected with a rod and secured with a threaded locking cap system. Post‐operative CT images were immediately acquired. No apparent canal breach was observed. Multiple screws in one dog demonstrated suboptimal bone purchase that required revision surgery. Postoperatively, one dog developed a seroma, which resolved. In one fixation, a screw broke after suspected bone healing was already achieved, so no revision surgery was required. All other dogs recovered uneventfully. One dog with negative nociception before surgery did not improve. All other dogs remained ambulatory or regained the ability to ambulate. This descriptive study demonstrated that PSF could successfully stabilize vertebral fracture/luxations in the thoracolumbar spine of dogs. The PSF system provides a clinically rigid fixation system that allows for early return to function. The PSF system allows timely and efficient adjustment of screws, as opposed to the modification of implants and PMMA fixations that can be challenging and time‐consuming.

## (P 33)

### Relapsing meningoencephalomyelitis of unknown origin: Neurological presentation, imaging characteristics and treatment

#### R. Aragão, DVM^1^ ,^2^, A. Cocchetto, DVM^1^ , G. Bertolini, DVM, Ph.D^3^ , L. Ventura, DVM^4^ , M. Menchetti, DVM, Ph.D, Dipl ECVN^1^

##### ^1^Neurology and Neurosurgery Division, San Marco Veterinary Clinic, Veggiano (PD), Italy; ^2^CIISA‐Center for interdisciplinary research in animal health, Faculty of Veterinary Medicine, University of Lisbon, Lisbon, Portugal; ^3^Dagnostic and Interventional Radiology Division, San Marco Veterinary Clinic, Veggiano (PD), Italy; ^4^Department of Statistical Sciences, University of Padova, Padova, Italy

Meningoencephalomyelitis of Unknown Origin (MUO) is a challenging disease to treat and many dogs eventually relapse, die or are euthanised. The aim of this study is to describe and compare the clinical presentation, magnetic resonance imaging (MRI) findings, cerebrospinal fluid (CSF) analysis and treatment protocols used between dogs with MUO that survived, relapsed or died. Medical records of 52 client‐owned dogs with a diagnosis of MUO presented from May 2017 to December 2021 were reviewed retrospectively. 24/52 dogs were successfully treated (46%), 13/52 relapsed (25%) and 15/52 died or were euthanised because of the disease (29%). The type of onset of neurological signs, presence of seizures and/or hyperesthesia were not predictive of the outcome. 67% of dogs treated with corticosteroids alone were euthanised or died (10/15) [*P* = 0.0005]. 62% of dogs that relapsed were treated with 3 or more drugs (8/13) [*P* = 0.001]. The most prevalent MRI lesion localisations were the cerebral hemispheres (63%), thalamus (40%) and spinal cord (44%). Multifocal lesions, brain herniation, presence of meningeal and parenchymal contrast enhancement, and type and cell count of CSF pleocytosis were not associated with a higher risk of relapse or death. According to the literature, the prognosis for MUO is considered generally poor and the cornerstone treatment modality is a multimodal immunosuppressive therapy. The therapy of steroids alone is generally considered not sufficient to successfully treat MUO. However, if the disease is particularly aggressive, the relapse is difficult to treat despite 3 or more drugs.

## (P 34)

### Predictive value of acute phase protein for the outcome of meningoencephalitis of unknown origin: A retrospective study

#### A. Cocchetto, DVM^1^ , R. Aragão, DVM^1^ ,^2^, L. Ventura, Full Professor^3^, M. Menchetti, DVM, Ph.D, Dipl ECVN^1^

##### ^1^Neurology and Neurosurgery Division, San Marco Veterinary Clinic, Veggiano (PD), Italy; ^2^CIISA‐Center for interdisciplinary research in animal health, Faculty of Veterinary Medicine, University of Lisbon, Lisbon, Portugal; ^3^Department of Statistical Sciences, University of Padova, Padova (PD), Italy

Meningoencephalitis of unknown origin (MUO) is one of the most common non‐infectious inflammatory diseases of the central nervous system. Acute phase proteins (APPs) are serum proteins, which change in concentration following inflammation and are highly sensitive indicators of systemic inflammation but with low specificity. Currently, their usefulness as biomarkers of MUO is not known. A retrospective case cohort study was designed with the aim to investigate the serum APPs in dogs with MUO. Secondarily, the aim of the study was to compare the APP profile between dogs successfully treated (group 0) with dogs relapsing of MUO (group 1) or euthanized/deceased because of MUO (group 2). Fifty‐two client‐owned dogs with a diagnosis of MUO were enrolled in the study, 24 of them were successfully treated (46%), 13 relapsed (25%) and 15 died/were euthanized because of MUO (29%). At the time of relapse, a higher level of IgM was associated with higher risk of relapse (*P* = 0.042). Despite a not fully statistical significance, a higher value of ferritin at the time of diagnosis was associated with higher risk of relapse or death (*P* = 0.052) and a higher level of neutrophils at the time of recurrence of neurological signs was possibly associated with higher risk of relapse of MUO (*P* = 0.09). These findings suggest that hyperferritinemia is a potential prognostic indicator for death and/or relapse in dogs diagnosed with MUO. Neutrophilia and higher levels of IgM could be used as indicators of relapse in dogs with a clinical suspicion of relapsing MUO.

## (P 35)

### Inflammatory biomarkers in discospondylitis: retrospective case control study of 149 dogs

#### G. Galli, DVM^1^ , A. Zoia, DVM, Ph.D, Dipl ECVIM‐CA^2^ , L. Ventura, Full Professor^3^, M. Menchetti, DVM, Ph.D, Dipl ECVN^1^

##### ^1^Neurology and Neurosurgery Division, San Marco Veterinary Clinic, Veggiano (PD), Italy; ^2^Division of Internal Medicine, San Marco Veterinary Clinic, Veggiano (PD), Italy; ^3^Department of Statistical Sciences, University of Padova, Padova (PD), Italy

Discospondylitis is an infection of the vertebral endplate comprising secondarily the intervertebral disc; it can be caused by bacterial or, less commonly, fungal or algal agents. Acute phase proteins (APP) are serum proteins whose concentration changes in response to the immune system's inflammation or stimulation regardless of the stimulus. Increased leucocyte count and C‐reactive protein titer have been used in human medicine as diagnostic parameters of discospondylitis in addition to clinical and neurological examination. The aim of this study is to assess the inflammatory biomarkers in dogs with discospondylitis. A retrospective case control study was conducted. Complete bloodwork of 149 dogs with a confirmed diagnosis of discospondylitis were examined and compared to bloodwork of a matched control group of dogs suffering from other diseases. Median leukocytes, neutrophils and monocytes values were significantly higher in dogs with discospondylitis (*P* < 0.05). Among APPs, in the discospondylitis group, the values of C‐reactive protein and haptoglobin were significantly increased, while albumin was significantly decreased (*P* < 0.05). Conversely, values of fibrinogen, ferritin and transferrin did not differ significantly between the two groups of dogs. Values of alpha2‐globulines, globulins, IgG and total proteins were significantly increased (*P* < 0.05). Consequently, albumin/globulin values were also significantly decreased (*P* < 0.05) in dogs affected by discospondylitis. In suspected cases of discospondylitis, alterations of the leukogram, specific APPs and globulins may significantly help in the diagnosis and differentiation from other disease processes.

## (P 36)

### Opportunistic *Candida albicans* granulomotous meningoencephalitis and adjacent osteolysis in an immunocompromised bullterrier

#### A. Siauciunaite, Med Vet^1^, A. Oevermann, Dr.Med Vet, DECVP, Prof.^2^, K. Beckmann, Dr.Med Vet, DECVN^1^ , S. Meunier, Dr.Med Vet, DACVIM^3^ , M. Waschk, Dr.Med Vet, DECVDI^4^

##### ^1^Section of Neurology and Neurosurgery, Small Animal Clinic, Vetsuisse Faculty, University of Zurich, Zurich, Switzerland; ^2^Neurocenter, Department for Clinical Research and Public Health, Vetsuisse Faculty Bern, University of Bern, Bern, Switzerland; ^3^Clinic for Small Animal Internal Medicine, Vetsuisse Faculty Zurich, University of Zurich, Zurich, Switzerland; ^4^Department of Diagnostic and Clinical Services, Clinic for Diagnostic Imaging, Vetsuisse Faculty Zurich, University of Zurich, Zurich, Switzerland

Opportunistic Candida infection of the central nervous system is an emerging disease in humans with immunosuppression and has recently been reported in two dogs without bony changes. Calvarial bone lysis in candida infections in humans is rare and limited to chronic cases. Risk factors for opportunistic Candida infection include corticosteroid, cyclosporine treatment and leukopenia. We report a case of a 10‐year‐old female spayed Bullterrier who was presented with multifocal neurological signs and seizures. The patient was diagnosed with immune‐mediated hemolytic anemia 6 months prior and had been treated with immunosuppressive medication including corticosteroids and cyclosporine. Under treatment the dog developed severe leukopenia, generalized seizures, and intermittent neurological deficits. At presentation in our hospital the patient showed bilateral sanguineous nasal discharge, generalized lymphadenomegaly and multifocal neurological deficits. MRI and CT examination showed multifocal intra‐axial lesions accentuated in the forebrain with severe meningitis. Additionally, extensive multifocal aggressive bone lysis of the cribriform plate, destructive rhinitis, and sinusitis, a heterogenous nasopharyngeal mass, and associated regional lymphadenomegaly. Histology and bacteriology of the nasopharyngeal mass revealed Candida albicans infection with bacterial co‐infection. The patient responded transiently to systemic voriconazole and local itraconazole treatment and was euthanized due to clinical deterioration. Postmortem examination confirmed granulomatous meningitis, fungal hyphae and associated polio‐ /leukoencephalomalacia. This is the second report about opportunistic candida infection causing granulomatous meningoencephalitis in dogs. In contrast to the first report, we could also find aggressive bone lyses, a rare feature of chronic candida infection in humans.

## (P 37)

### Choroid plexus cyst in a dog: Clinical presentation, imaging and histopathological findings

#### M. Benito, DVM MRCVS^1^ , P. Smith, DVM MRCVS PhD Dip ECVN^1^ , F. Constantino Casas, MVZ MRCVS PhD^2^ , D. Sanchez Masian, DVM MRCVS Dip ECVN^1^

##### ^1^Hamilton Specialist Referrals, Dept of Neurology and Neurosurgery, High Wycombe, UK; ^2^The Queen's Veterinary School Hospital, Department of Veterinary Medicine, Cambridge University, Cambridge, UK

A 3‐year‐old, female, Cocker spaniel was presented to the clinic with a clinical history of right temporal muscle atrophy over the preceding 3 weeks. Neurological examination indicated an isolated right‐sided trigeminal neuropathy, but further investigations were declined. The dog continued to deteriorate and was re‐examined 8 weeks later. Neurological examination at this time revealed severe right temporal muscle atrophy, with no facial sensation on the right and an absent palpebral reflex and menace response on the same side; there was also a vestibular ataxia, with an absent oculocephalic reflex in the right eye and reduced postural reactions on the right. An MRI scan of the brain revealed a solitary right‐sided extra‐axial lesion at the level of the pons, with compression of the brainstem and dorsal displacement of the cerebellum. The lesion was well‐defined and was isointense with the CSF in all sequences, with mild peripheral contrast enhancement. The dog was euthanised without treatment and underwent post‐mortem examination, revealing a thin‐walled, fluid‐filled structure at the pons. There was no identifiable connection with the ventricular system. Histopathological examination showed a cyst wall lined by a simple squamous epithelium, with areas of cuboidal epithelium. Within the cyst, there were areas of fibrovascular stroma supporting tubular‐like structures covered by simple cuboidal epithelium. There were neither mitotic figures nor morphological features typical of neoplasia, findings which indicate a diagnosis of choroid plexus cyst. This benign lesion is very rarely reported, and the current case is unique in having no connection with the ventricular system.

## (P 38)

### A fatal case of doramectin intoxication in a main coon kitten harboring ABCB1 genetic defect

#### A. Siauciunaite, Med Vet^1^, A. Kehl, Dipl. Biol.^2^, E. Mueller, Dr.Med Vet^2^, L. Golini, Dr.Med Vet, DECVN^1^

##### ^1^Section of Neurology and Neurosurgery, Small Animal Clinic, Vetsuisse Faculty, University of Zurich, Zurich, Switzerland; ^2^Laboklin GmbH & Co.KG, Bad Kissingen, Germany

Avermectin intoxication in cats has rarely been reported in veterinary medicine, and the ABCB1 status of purebred cats is not a routine investigation. A 6‐month old male non‐castrated Maine Coon was presented due to hyperacute onset with rapid progression of obtundation, reduced general condition, and hypersalivation after subcutaneous injection of parasiticide treatment of Doramectin by the referring veterinarian. At admission, hypothermia (37.5), bradycardia (144 bpm), stuporous mental status, coarse whole‐body tremors and jerk myoclonus of the head, generalized proprioceptive deficits, and absent menace reaction were presenting signs compatible with a multifocal forebrain disease. Bloodwork was unremarkable. Intralipid and antiepileptic medications were administered. Brain MRI and CSF were normal. Despite supportive care, he continued to deteriorate and died within 24 h. A genetic test showed the presence of the ABCB1 c.1930_1931delTC variant in homozygous status. A previous report has been published on avermectin intoxication in cats mainly characterized by obtundation and coma. In the present case, existence of ABCB1 gene defect can be postulated to play a role in the fatal outcome of our patient. ABCB1 gene defect prevents functionality of p‐glycoprotein resulting in toxic accumulation of multiple substances within the central nervous system. With this case report we wish to bring attention to the presence of the ABCB1 variant in some feline breeds, and to raise awareness between the veterinary community about the need of a better understanding of the impact of this mutation within feline patients.

## (P 39)

### Successful medical treatment of a suspected idiopathic hypertrophic ganglioneuritis in one dog

#### L. Espino, DVM PhD^1^ , M. Suarez, DVM PhD^1^ , N. Miño, DVM PhD^1^ , M. Vila, DVM^1^

##### ^1^Hospital Veterinario Universitario Rof Codina, Universidad de Santiago de Compostela, Lugo, Spain

Focal hypertrophic inflammatory neuropathy is a rare clinical entity in people and dogs. Information on treatment and outcome in dogs is limited and in the only case reported, describing a unilateral chronic ganglioneuritis, the resolution of the clinical signs was achieved only after surgical removal of the nerve root. This report describes the clinical signs, computed tomographic findings and clinical and imaging outcome after successful medical treatment in a dog with a suspected idiopathic hypertrophic ganglioneuritis. A 6‐year‐old spayed female Maltese bichon was examined because of a four‐month history of cervical hyperesthesia. A severe neck pain was the only abnormality on neurological examination. Computed tomography revealed a soft tissue mass (0.6 cm diameter) at the level of C5‐C6 left intervertebral foramen, with a marked homogeneous contrast enhancement, causing a moderate spinal cord compression and widening of the intervertebral foramen. The main differential diagnosis was a spinal nerve neoplasia and less likely an inflammatory neuritis. A palliative treatment with glucocorticoids was instaured because the owners refused the surgical biopsy. On recheck CT after 3 months, the mass disappeared, and the only abnormality was a widening intervertebral foramen. One year after discontinuation of treatment there was no recurrence of clinical signs. In people with hypertrophic inflammatory neuropathy, medical treatment led to improvement of clinical signs in most cases. To the author's knowledge, this is the first report in which medical treatment results in a complete remission of clinical and imaging signs in a dog with a suspected idiopathic hypertrophic ganglioneuritis.

## (P 40)

### Intradural‐extramedullary inflammatory pseudotumor in the cervical spinal cord of a dog

#### H. Mendo, DVM^1^ , K. Matiasek, DVM, Full Professor^2^, B. Bravaccini, DVM^1^ , G. Dalla Serra, DVM^3^ , M. Frizzi, DVM^4^ , M. Menchetti, DVM, Ph.D, Dipl ECVN^1^

##### ^1^Neurology and Neurosurgery Division, San Marco Veterinary Clinic, Veggiano (PD), Italy; ^2^Section of Clinical and Comparative Neuropathology, Centre for Clinical Veterinary Medicine, Ludwig Maximilians University of Munich, Munich, Germany; ^3^Diagnostic Imaging and Interventional Radiology, San Marco Veterinary Clinic, Veggiano (PD), Italy; ^4^Surgery Division, San Marco Veterinary Clinic, Veggiano (PD), Italy

The Inflammatory Pseudotumor (IPT) is a rare lesion characterized by myofibroblastic spindle cells and inflammatory cells and there is ongoing debate about its inflammatory or neoplastic nature. In veterinary medicine, IPT has been rarely described. In this report, we describe the second case of an intradural‐extramedullary canine IPT. An 8‐year‐old, male, mixed‐breed dog was presented with a one‐month history of cervical hyperesthesia and tetraparesis. The dog was previously treated with steroid therapy at anti‐inflammatory dose, with partial response. The neurological exam revealed tetraparesis and mild proprioceptive ataxia in the four limbs. MRI (3T) of the cervical spine showed a dorsal compressive intradural‐extramedullary mass at the level of C1–C2, which had isointense signal with an hypointense ventral margin on T2W images and isointense signal on T1W, with strong contrast enhancement. A C1–C2 partial dorsal laminectomy was performed, and the lesion was removed en bloc. Histopathological and immunohistochemical analysis revealed the proliferation of spindle cells with plasma cell and neutrophilic infiltration, positive to IBA‐1, MHC‐II and CD‐18. No etiologic agent was identified. The diagnose was consistent with IPT. The dog became tetraplegic after surgery and improved during the first month. This case highlights the difficulty to diagnose the IPT without the histopathologic examination. The prognosis of spinal cord IPT is mostly guarded. However, if the lesion appears to be extramedullary in nature, a possible en bloc resection is still possible.

## (P 41)

### Suppression of inner ear signal intensity on flair MRI in 41 cats with vestibular disease

#### S. Everest, BSc, BVM&S^1^ , L. Gaitero, DVM, dipl. ECVN^2^ , G. Monteith, MSc^2^ , F. Samarani, DVM, dipl. ECVN^2^

##### ^1^Ontario Veterinary College Health Science Centre, University of Guelph, Guelph, Canada; ^2^Department of Clinical Studies, University of Guelph, Guelph, Canada

Otitis media/interna (OMI) is the most common cause of peripheral vestibular disease in cats. The inner ear contains endolymph and perilymph, with perilymph being very similar in composition to cerebrospinal fluid (CSF). As a very low protein fluid, it would be expected that normal perilymph should suppress on fluid‐attenuated inversion recovery (FLAIR) magnetic resonance imaging (MRI) sequences. Therefore, MRI FLAIR sequences should provide a non‐invasive way of diagnosing inflammatory/infectious diseases such as OMI in cats, something that has previously been demonstrated in humans, and in dogs. This was a retrospective, cohort study, in which 41 cats were included. They were grouped into 4 groups based on diagnosis: (a) clinical OMI, (b) inflammatory central nervous system (CNS) disease, (c) non‐inflammatory structural disease, (d) normal brain MRI (control group). Transverse T2‐weighted and FLAIR MRI sequences at the level of the inner ears were compared in each group. The inner ear was selected as a region of interest (ROI) using HorosTM, with a FLAIR suppression ratio calculated to account for variability in signal intensity between MRI's. This FLAIR suppression ratio was then compared between groups. The OMI group had significantly lower FLAIR suppression scores compared to all other groups. The CSF cell count was significantly increased in the OMI and inflammatory CNS disease groups compared to the control group. In conclusion, this study demonstrates the utility of MRI FLAIR sequences in diagnosing OMI in cats, similarly to in humans and dogs.

## (P 42)

### Paracetamol add‐on treatment for perioperative pain management in dogs undergoing single‐site thoracolumbar hemilaminectomy: A prospective clinical study

#### N. Burger, DVM^1^ , T. Bosmans, DVM, PhD^1^ , S. Bhatti, DVM, PhD^1^ , S. Ooms, DVM^1^ , B. Broeckx, DVM, PhD, Prof.^2^, I. Polis, DVM, PhD, Prof.^1^, L. Van Ham, DVM, PhD, Prof., Dipl ECVN^1^ , I. Cornelis, DVM, PhD, Dipl ECVN^1^

##### ^1^Small Animal Department, Faculty of Veterinary Medicine, Ghent University, Merelbeke, Belgium; ^2^Department of Veterinary and Biosciences, Faculty of Veterinary Medicine, Ghent University, Merelbeke, Belgium

The aim of the study was to evaluate whether paracetamol as an adjunct to NSAID and opioid analgesia might limit the requirements for intraoperative fentanyl and postoperative opioids, in dogs undergoing single‐site thoracolumbar hemilaminectomy. A prospective, double‐blinded, randomized, clinical study was set up including 12 client‐owned dogs that underwent surgical decompression for a thoracolumbar intervertebral disc extrusion (2020–2022). Dogs were randomly assigned to two multimodal analgesia groups: NSAID + paracetamol (group NP) and only NSAID (group N). All dogs received methadone at induction. Intra‐ and postoperative analgesic assessments were based on respectively clinical evaluation and application of the Glasgow Composite Measure Pain Scale‐Short Form on regular intervals up to 20 hours after extubation. Intraoperative and postoperative rescue analgesia consisted of fentanyl and methadone, respectively. The occurrence of adverse effects was recorded. One dog from group N was excluded as the protocol was not followed, leading to a total sample size of 11 dogs. No statistically significant difference between both groups was found for the intraoperative need for fentanyl (*P* = 0.18). The probability of having to administer postoperative rescue analgesia was significantly higher in group N (*P* = 0.01). No serious adverse effects were reported, and there was no significant difference regarding the development of side effects between the two groups (*P* = 0.55). Despite multimodal perioperative pain management consisting of a full μ‐agonist opioid, a NSAID and paracetamol in dogs undergoing single‐site thoracolumbar hemilaminectomy, intraoperative rescue analgesia was still required. The need for postoperative opioid analgesia was significantly higher in group N comparted to group NP.

## (P 43)

### Clomipramine for the treatment of behavioral comorbidities in six dogs withh idiopathic epilepsy

#### M. Tenger, DVM^1^ , S. Van Meervenne, DVM, DIPL ECVN^2^

##### ^1^Anicura Djursjukhuset Albano, Stockholm, Sweden; ^2^Anicura Kalmar Djursjukhus, Kalmar, Sweden

Introduction: The recent recognition of behavioral comorbidities in dogs with idiopathic epilepsy (IE) urges the need for appropriate therapy. Clomipramine is a tricyclic antidepressant used for behavioral disorders in dogs. The aims of this study is to describe the effect of clomipramine on behavioral comorbidities and seizure frequency in dogs with IE. Method: Retrospective study of dogs presented to two animal hospitals in Sweden during 2016‐2018 with presumed IE and behavioral comorbidities severely affecting the dogs and owners' quality of life. Inclusion criteria were treatment with clomipramine together with at least one anti‐epileptic drug due to presumed IE and a follow up time for at least 6 months after start of clomipramine treatment. Results: Six dogs were included in the study. Four owners stated that their dog's behavior was at least 50% better than before treatment, mean follow up time for these dogs were 3.6 years. Two dogs did not respond to add on with clomipramine and the treatment was discontinued. Two dogs decreased in seizure frequency after treatment with clomipramine, three had increased seizure frequency and for one dog seizure frequency didn ´t change. Discussion: Clomipramine can be an option for dogs suffering from IE and behavioral comorbidities. Due to the controversial concern of an increase in seizure frequency when administering clomipramine in patients with IE, future prospective studies with other behavioral drugs, like fluoxetine, could be considered.

## (P 44)

### Parenchymatous malignant neuroendocrine tumor with an originally cystic appearance in the para‐sellar region of a dog

#### J. Espinosa, DVM MRCVS^1^ , O. Travetti, DVM PhD DipECVDI MRCVS^2^ , G. Scarin, DVM MRCVS^3^ , P. Graham, BVMS PhD Cert VR DipECVCP MRCVS^3^ , C. Bianco, DVM PhD MRCVS^3^ , J. Goh, Student from University of Nottingham^3^, A. Wessmann, DrMedVet DipECVNPGCertAcPrac FHEA MRCVS^1^

##### ^1^Neurology & Neurosurgery Service, Pride Veterinary Centre, Derby, United Kingdom; ^2^Diagnostic Imaging Service, Pride Veterinary Centre, Derby, United Kingdom; ^3^School of Veterinary medicine and Science (SVMS), University of Nottingham, Sutton Bonington, United Kingdom

Hypophyseal adenomas are the most common tumors affecting the hypophyseal fossa in dogs. Rarely reported, non‐adenohypophyseal tumors of the sellar region mimic non‐secreting hypophyseal adenomas. This case report describes the clinical, repeat magnetic resonance (MRI) and histopathological findings of this uncommon neoplasia affecting the sellar region in a dog. A 6‐month‐old male cocker spaniel presented with a 2‐month history of progressive blindness as the only deficit. MRI showed a large, suspected benign, cystic lesion occupying the mid‐sagittal para‐sellar and para‐chiasmatic regions compressing the optic chiasm and optic nerves yet sparing the hypophysis. The dog developed bilateral mydriasis and divergent strabismus over the following months. A sudden, more marked deterioration 18 months after the first MRI was characterized by behavior changes and lethargy. Follow‐up MRI showed a malignant transformation of the previous cystic mass leading to a large parenchymatous lesion of intradural origin compressing the thalamic region; the neurohypophysis was not displaced. The dog deteriorated and was euthanised. The histology and the cytology were suggestive of a malignant neuroendocrine tumor; the immunohistochemistry was positive for synaptophysin, negative for pancytokeratin whilst C‐Kit revealed rare positive cells. Combined with the MRI features, a non‐hypophyseal malignant neuroendocrine tumor was diagnosed. This case report raises the possibility of a malignant transformation of a suspected benign cystic lesion more than a year later. Moreover, the position of the neurohypophysis on MRI was helpful in excluding an adenohypophyseal origin. Histopathological findings should always be interpreted together with clinical and imaging features to achieve the most accurate diagnosis.

## (P 45)

### Acute idiopathic polyradiculoneuritis associated to glucantime treatment

#### A. Recio Caride, DVM, Dip ECVN^1^

##### ^1^Clínica Veterinaria Levante, San Javier (Murcia), Spain

Acute idiopathic polyradiculoneuritis has been proposed to be associated with recent vaccination, upper respiratory and gastrointestinal infections, breed and season or raw chicken consumption. A 6 year old female Labrador retriever was referred for a 2 weeks history of symmetrical weakness progressing to non‐ambulatory flaccid tetraparesis over the first days. The dog was recently diagnosed with Leishmania and was under standard treatment with Glucantime. Neurological examination confirmed a generalized neuromuscular disease with dysphonia, preserved tail wagging and urine and fecal control. A complete blood analysis revealed a non‐regenerative anemia with increased globulin fraction level. Electrophysiologycal tests were performed showing presence of spontaneous activity on EMG, decreased M wave amplitude, absence of F waves and normal motor conduction velocity. CSF revealed an albuminocytological dissociation. Clinical signs and findings of diagnostic tests were consistent with an acute polyradiculoneuritis. Glucantime therapy was discontinued, and symptomatic therapy was applied. The dog showed an evident improvement over the next 2 weeks, regaining ambulatory. As the dog was stabilized, glucantime therapy was restarted and in less than 24 h a sudden relapse with even more severe symptoms was notified and the dog was again hospitalized confirming the same findings as previously. Glucantime was discontinued definitively, and the symptoms disappeared in a few days again and remain still without symptoms after 2 years. The use of glucantime at the onset and at the relapse of the symptoms could consider this medication as a potential risk factor in this case, but should be confirmed by further studies.

## (P 46)

### Middle ear squamous cell carcinoma in a 6‐year‐old weimaraner: Clinical presentation, mri and post‐mortem findings

#### J. Amey, MA, VetMB, MRCVS^1^ , A. To, BVetMed, MRCVS^1^ , G. B. Cherubini, DVM, DECVN, FRCVS^1^ , P. Mantis, DVM DipECVDI FHEA FRCVS^1^ , A. Morey Matamalas, DVM, MVM, MRCVS, AFHEA^2^ , M. Pereira, DVM, MSc WAH, MRCVS^2^

##### ^1^Dick White Referrals, Six Mile Bottom, UK; ^2^Veterinary Pathology, School of Veterinary Medicine and Science, University of Nottingham, Nottingham, UK

Introduction: This study describes the clinical, MRI and histopathology findings from a 6‐year‐old male entire Weimaraner with middle ear squamous cell carcinoma. Methods: The animal presented with;3 months' history of upper respiratory stertor and;2 days' history of vestibular ataxia, right Horner's syndrome, pain when opening the mouth and change in bark. Neurological exam further revealed reduced mentation, low head carriage, rotary nystagmus, reduced right palpebral reflex and reduced swallow reflex. Ocular phenylephrine test was suggestive of post‐ganglionic Horner's syndrome. MRI showed an expansile mass arising from the right tympanic bulla causing osteolysis of the osseous bulla. Marked contrast enhancement was noted in the surrounding tissues and the thick adjacent meninges. FNA cytology was compatible with tympanokeratoma. The animal arrested after developing acute respiratory distress syndrome secondary to aspiration pneumonia. The animal then underwent post‐mortem examination. Results: Histopathology was consistent with right middle ear squamous cell carcinoma with severe tympanic bone osteolysis. Lung pathology showed acute, unilateral bronchopneumonia compatible with aspiration pneumonia. Discussion/Conclusion: Squamous cell carcinomas are rarely described in the canine middle ear. A previous report described pain upon opening the dog's mouth, lethargy and inappetence. We present the MRI findings and histopathology of a case that also developed acute onset neurological signs, which have been recognized in other species. Squamous cell carcinomas although rare should be considered as a differential diagnosis for middle ear neoplasia

## (P 47)

### Relapses in idiopathic generalized tremor syndrome in dogs

#### F. Kajin, Dr^1^, N. Meyerhoff, Dr, Dipl. ECVN^1^ , S. Meller, PhD^1^ , R. Carlson, MLT^1^ , A. Tipold, Prof, Dipl. ECVN^1^ , H. A. Volk, Prof, PhD, Dipl. ECVN^1^

##### ^1^Department of Small Animal Medicine and Surgery, University of Veterinary Medicine, Hanover, Germany

Idiopathic generalized tremor syndrome (IGTS) are rare clinical entities. The aim of the current study was to evaluate and describe history, clinical and laboratory findings and relapses in dogs with IGST. Dogs clinically diagnosed with IGTS between 2012 and 2022 were included in this retrospective monocentric study. Dogs had to have unremarkable magnetic resonance imaging (MRI) of the brain. Dogs with electrolyte disturbances or a history of toxin exposure were excluded. History, clinical signs, complete blood count findings, cerebro‐spinal fluid (CSF) analysis, serum and CSF levels of IgA, as well as therapy, outcome and relapses were recorded. Eighteen dogs (16 females and 2 males) met the inclusion criteria. Seven dogs (39%) had an increased CSF nucleated cell count and two dogs (11%) had increased serum and CSF IgA values. Prednisolone was administered to all dogs in this study. Add‐on immunomodulatory drugs were given to six dogs (33%); ciclosporin (4), cytarabine (2) and mycophenolate mofetil (1). Short‐term (1–6 months) and long‐term outcome (=6 months) was available in 6 and 10/18 dogs respectively. 9/16 dogs had an excellent outcome and 7 had an acceptable outcome (characterized as having one or more recurrences). Seven dogs suffered at least one relapse, five had two, and three dogs relapsed more than twice. No statistically significant correlations were found between outcome and clinical or laboratory findings. Contrary to previous reports, a substantial number of dogs with IGTS suffered one or more relapses and required long‐term immunomodulatory therapy or additional drugs. No prognostic factors for IGTS were identified.

## (P 48)

### Surgical management of congenital thoracic vertebral malformations in three large breed dogs

#### F. Couto, DVM MRCVS^1^ , J. Tabanez, Neurology Registrar DVM MRCVS^2^ , C. Driver, BSc BVetMed (Hons) MVetMed PhD DipECVN MRCVS^1^

##### ^1^Lumbry Park Veterinary Specialists, Alton, United Kingdom; ^2^Fitzpatrick Referrals Orthopedics and Neurology, Godalming, United Kingdom

This retrospective case series aims to evaluate the long‐term outcome following decompression‐stabilization of complex thoracic vertebral malformations, characterized by combined failures of segmentation and formation, in three large breed dogs. All cases were presented with progressive, non‐painful, neurological deficits localizing to the T3–L3 spinal cord segments. MRI and CT scans revealed spinal cord compression at a single level, within the T8‐T13 region. Spinal cord compression was caused by a combination of ‘tethering’ of the spinal cord by nerve roots, over a wedge‐ shaped block vertebra, with vertebral canal stenosis. All three cases were treated surgically, with a combination of ventral decompression (via bilateral mini‐hemilaminectomy and partial lateral corpectomy) and concurrent vertebral stabilization with polyaxial screws and rods (one case) or threaded pins and bone cement (two cases). In one case, a titanium mesh laminoplasty was also performed. Transient postoperative neurological deterioration was seen in two cases. Follow‐up was obtained by repeat neurologic examinations up to 3, 6 and 12 months post‐operatively; these confirmed improved neurologic function with persisting mild ambulatory paraparesis in all cases. Two cases had follow‐up CT at 6‐ and 12‐months post‐operative, which revealed persisting increase in vertebral canal volume. Clinically relevant thoracic vertebral malformations, such as dorsal hemivertebra, are most commonly identified in ‘screw‐tailed’ brachycephalic dogs; however, such malformations are rarely reported in large breed dogs. Combined failure of vertebral formation and segmentation can cause ventral spinal cord compression and vertebral canal stenosis. Surgical decompression‐stabilization resulted in satisfactory outcomes in the three cases reported.

## (P 49)

### MRI, CSF and BAER changes in a dog with acute blindness and deafness due to systemic protothecosis

#### C. Taestensen, DVM^1^ , A. Steinmetz, DVM^1^ , C. Blobner, DVM^1^ , J. Stiller, Dipl. ACVIM (SAIM)^1^, S. Rau, DVM^2^ , I. Spitzbarth, Dipl.ECVP^2^ , T. Flegel, Dipl.ECVN, Dipl.ACVIM (Neurology)^1^

##### ^1^Department for Small Animals, Leipzig University, Leipzig, Germany; ^2^Institute of Veterinary Pathology, Leipzig University, Leipzig, Germany

A two‐year‐old female Rhodesian Ridgeback was presented with acute blindness and deafness. The dog had a history of chronic large bowel diarrhea for 3 months. On presentation a head tilt to the left, bilateral deafness and mild generalized ataxia was evident. The lesion was localized to the peripheral vestibular system. Ophthalmological examination revealed exudative retinal detachment. On brainstem auditory evoked response a flat‐line was evident on the left ear and a delayed wave I was evident on the right ear not until 90 dB. MRI of the head showed reduced T2 signal intensity of the left cochlea. CSF analysis revealed a severe eosinophilic pleocytosis of 3260 cells/μl with 63% eosinophils and a protein of 0.64 g/L. Coloscopy was performed, and colonic mucosal pinch biopsies were submitted for histologic evaluation. Histopathologic examination revealed colitis with lymphocytes, neutrophils and eosinophils. Numerous PAS‐positive oval‐shaped organisms and endosporulation were present. CSF culture revealed Prototheca species. Treatment was started 2 days before presentation with Itraconazole (10 mg/kg SID) and Doxycycline (11 mg/ kg BID). Nine days after discharge the dog showed neurological deterioration and was euthanized due to owner ‘s request. This case report illustrates a severe systemic protothecosis, which is a rare disease of people and animals. Several case reports and some cohort studies exist in the veterinary literature. In some patients deafness was evident and histological examination revealed inner ear involvement. To the authors knowledge this is the first report of MRI cochlear changes and BAER changes due to systemic protothecosis in a dog.

## (P 50)

### An unusual presentation of a choroid plexus tumor in a shetland sheepdog

#### N. Schneider, Resident ECVN^1^ , B. Parzefall, Diplomate ECVN^1^ , A. Blutke, German Specialist in Veterinary Pathology^2^

##### ^1^Small Animal Clinic Oberhaching, Oberhaching, Germany; ^2^Institute of Veterinary Pathology, Center for Clinical Veterinary Medicine, Ludwig‐Maximilians Universität München, Munich, Germany

Choroid plexus tumors (CPT) account for 7%–10% of intracranial, primary tumors and are commonly described as intraventricular mass lesions, frequently accompanied by hydrocephalus. A 3‐year‐old Shetland sheepdog was presented for slowly progressive lethargy, vision impairment and cognitive deficits. Neurological examination was consistent with a multifocal central nervous system (CNS) disease. Magnetic resonance imaging (MRI) revealed multifocal, intra‐axial lesions within the brain and a subdural fluid accumulation (SFA) overlying the left parietotemporal lobe leading to a compression of the adjacent cerebral hemisphere and left lateral ventricle. Cerebrospinal fluid (CSF) analysis showed a mononuclear pleocytosis with negative results for infectious agents. The dog was treated with prednisolone but further deteriorated and therefore underwent burr hole craniotomy with puncture of the SFA which revealed to be CSF‐like fluid. The dog improved post‐surgically but then worsened again within the following 4 weeks despite additional cytarabine therapy and was euthanized. Necropsy revealed a resolution of the SFA, multifocal dural hemorrhage, a marked ventricular dilation, a scant diffuse neoplastic infiltration of the ventricles, the cerebral, cerebellar, and spinal cord meninges and the cribriform plate. Histopathological findings were consistent with a diffuse, infiltrative choroid plexus tumor involving the entire surface of the ventricular system, meninges and the spinal cord, as well as multifocal infiltration of the adjacent neuroparenchyma. In conclusion, this is the first report about a choroid plexus tumor presenting as a diffuse CNS neoplasia that is associated with a subdural fluid accumulation that could already be identified on MRI.

## (P 51)

### Hamartosis of the thoracolumbar spine in a 3‐month old rhodesian ridgeback

#### J. Hart, Dr.^1^, C. Günther, Dr., FTA^1^ , K. Matiasek, Prof., Dr., FTA^2^ , S. Rupp, Dr., FTA^1^ , T. von Klopmann, Dr., DipECVN^1^

##### ^1^Tierklinik Hofheim, Hofheim am Taunus, Germany; ^2^Institut für Tierpathologie, Ludwig‐Maximilians‐Universität, München, Germany

Hamartomas are rare malformations which are considered to be a nonneoplastic proliferation of tissue and cells indigenous to the affected organ. Lesions may progress for several years without causing clinical signs depending on the localization or increase in size. This case report describes the clinical, imaging, and histopathological findings of multiple osseous Hamartomas in the thoracolumbar spinal cord of a 3‐month‐old Rhodesian Ridgeback puppy presented because of progressive paraparesis and proprioceptive ataxia. After clinical and neurological examination, as well as a complete blood work the MRI study revealed diffuse T2‐weighted hyperintensities of Th12, L4 and S2 vertebral bodies, and the vertebral arcus of Th13. The outer border of the cortex of the right vertebral arcus of Th12, Th13 and L4 was irregular and hyperostotic consistent with substantial bony proliferation into the vertebral canal and marked compression of the spinal cord. A whole‐ body‐CT examination was carried out to further characterize the lesion and to exclude other pathologies. Histopathology of one vertebra showed diffuse moderately thickened cortices and marrow cavities appeared multifocally markedly expanded presenting with diffuse mild to moderate myxoid changes. These changes were accompanied by moderately to markedly, tortuous dilated thin‐walled blood vessels corresponding to abnormal venous plexuses and lipid and cartilaginous emboli. These findings were most consistent with osseous hamartoma. In conclusion, hamartoma should be kept in mind as a differential diagnosis in young dogs presenting with neurological signs.

## (P 52)

### Application accuracy of a frameless optical neuronavigation system for orientation during craniotomies

#### S. Gutmann, DVM^1^ , M. Heidhoffer^1^, R. Möbius, MSc^2^ , T. Siegel, DVM^1^ , T. Flegel, Dipl. ECVN, Dipl. ACVIM (Neurology)^1^

##### ^1^Department for Small Animals, Faculty of Veterinary Medicine, Leipzig University, Leipzig, Germany; ^2^Department of Neurosurgery, University Clinic of Leipzig, Faculty of Medicine, Leipzig, Germany

For neurosurgical interventions, such as brain biopsies or removal of brain tumors, modern neuronavigation systems are becoming increasingly important in small animal medicine. Those systems create a virtual reality image of the brain allowing the surgeon to track instruments in real time. Aim of the study was to determine the application accuracy of the frameless optical neuronavigation system “NAV 1 optical” of the company STORZ for targeting predefined points on the skull surface for surgical access to the cerebrum, cerebellum and pituitary gland. The optical neuronavigation systems consist of a dual camera system emitting infrared light, a special computer software, a patient tracker with reflective markers and the probe with three reflective spheres. On five canine cadaver heads 10 target points (Os parietale (6), Os occipitale (2), Os basisphenoidale (2)) were marked in a preoperative CT scan. These target points were found on the cadaver skulls using the optical neuronavigation system and then a small borehole (1.5 mm) was drilled at the target points. Subsequently, another CT scan was made. Both CT data sets were fused in the neuronavigation software and the actual target point coordinates were identified. The target point deviation was determined as the difference between the planned and drilled target point coordinates. The calculated deviation was compared between two examiners. The analysis of the target point accuracies of all dogs in both examiners taken together showed a median target point deviation of 1.57 mm (range width 0.42‐5.14 mm). No significant differences were found between the investigators or the target regions.

## (P 53)

### Ipsilateral mydriasis associated with naturally occuring ischaemic infarcts affecting the cerebellar interposital nucleus in two dogs

#### C. Danciu, DVM(Hons), MRCVS^1^ , J. Fenn, BVetMed, MVetMed, DipECVN, FHEA, MRCVS^1^ , E. Beltran, Ldo Vet, DipECVN, PGCertVetEd FHEA, MRCVS^1^

##### ^1^Department of Clinical Science and Services, Royal Veterinary College, University of London, Hatfield, United Kingdom

Anisocoria can be physiological, pathological, or pharmacological in origin. Experimental studies described anisocoria associated with cerebellar lesions affecting the fastigial and interposital nuclei in cats, however to the authors' knowledge, this has not yet been reported in naturally occurring lesions in dogs. Two dogs, a 10‐year‐old male neutered Collie Cross (Dog 1) and an 8‐year‐old female neutered Greyhound (Dog 2) presented following a peracute onset of cerebellar ataxia. Neurological examination of Dog 1 revealed anisocoria (left pupil mydriatic and slightly responsive to light), spontaneous rotatory nystagmus with fast phase to the left and absent postural reactions in the left thoracic and pelvic limbs. Neuroanatomical localisation was left cerebellum with paradoxical vestibular syndrome. Examination of Dog 2 revealed anisocoria with the left pupil being mydriatic and responsive to light, left positional ventral strabismus, absent menace response in the left eye and postural reaction deficits on the left side. Neuroanatomical localisation was left cerebellum and brainstem. In both cases, MRI identified a left‐sided, sharply demarcated, wedge‐shaped T2W/FLAIR hyperintense and T1W hypointense lesion in the left cerebellar hemisphere, with no‐to‐minimal contrast enhancement and restricted diffusion. In both dogs, the lesion was associated with a well‐demarcated extension into the cerebellar nuclei, in the location of the left interposital nucleus. Imaging findings were consistent with a left cerebellar ischaemic territorial infarct. These two dogs demonstrate the occurrence of anisocoria with ipsilateral mydriasis associated with a naturally occurring cerebellar ischaemic infarct in the region of the interposital nucleus.

## (P 54)

### Narcolepsy/cataplexy in a rottweiler with degenerative encephalopathy

#### A. Recio Caride, DVM, Dip ECVN^1^ , M. Pumarola, DVM, PhD, Dip ECVP^2^

##### ^1^Clínica Veterinaria Levante, San Javier (Murcia), Spain; ^2^Department of Animal Medicine and Surgery UAB Barcelona, Barcelona, Spain

Narcolepsy is a sleep disorder caused by the absence or scarcity of hypocretin or its specific receptor. In dogs, two forms of Narcolepsy can occur, congenital and acquired. The acquired form has been described in few cases, secondary to a case of postvaccinal distemper, a pituitary macroadenoma and more recently to various presentations of meningoencephalitis of unknown origin. A 6‐year‐old female Rottweiler was referred for a month history of increasing events of daytime sleepiness, accompanied by episodes of cataplexy. The neurological examination was apparently normal, but during the consultation the dog showed some sleep crisis and cataplexy episodes. These neurological signs were consistent with a lesion involving the hypocretinergic system at the hypothalamic‐brainstem axis. The magnetic resonance imaging showed a generalized cortical cerebral and cerebellar atrophy, with increased CSF fluid signal, widening of sulci and folia, smaller interthalamic adhesion, as well as increased subarachnoid space at brainstem level. CSF analysis was unremarkable. Genetic tests for Rottweiler degenerative diseases and for the hypocretin receptor 2 gene mutation were performed and were negative. Due to a suspicion of a progressive juvenile degenerative disease, the owner rejected any symptomatic therapy and the dog was euthanized. Histopathological findings showed incipient signs of neuronal paleness, chromatolysis and microspongiosis associated to a marked astrocytic gliosis and moderate microgliosis. This lesional pattern affected selectively, and with a bilateral and symmetrical pattern, some nuclei of the hypothalamus and brainstem, and was consistent with a neuronal degenerative disease. To authors knowledge this is the first description of a narcolepsy related to a degenerative disease and should be included into the differential diagnose in narcolepsy cases.

## (P 55)

### Clinical findings, MRI features, and short‐term outcome of medium‐to‐large breed dogs with hydrocephalus

#### J. Bongers, MSc^1^ , M. Bernardini, DVM, DECVN^2^ ,^3^, C. Rusbridge, BVMS, PhD, DECVN, FRCVS^4^ ,^5^,E. Boudreau, DVM, PhD, DACVIM^6^ , K. Santifort, MSc^7^ ,^8^, E. Glass, MS, DVM, DACVIM^9^ , A. Shores, DVM, MS, PhD, DACVIM^10^ , L. Green, DVM, DACVIM^11^ , M. Ortega, LV, MSc, DECVN^12^ , G. Dutil, Dr. Med Vet^13^, B. Lindsay, BSc/BVMS, DACVIM^14^ , M. Pasierbinska, DVM^1^ , C. Stalin, VetMB, BA, DECVN, MRCVS^1^ , R. Gutierrez‐Quintana, MVZ, MVM, DECVN, MRCVS^1^

##### ^1^School of Veterinary Medicine, University of Glasgow, Glasgow, United Kingdom; ^2^Anicura Portoni Rossi Veterinary Hospital, Bologna, Italy; ^3^University of Padova, Padua, Italy; ^4^Fitzpatrick Referrals, Eashing, United Kingdom; ^5^School of Veterinary Medicine, University of Surrey, Guildford, United Kingdom; ^6^College of Veterinary Medicine & Biomedical Sciences, Texas A&M University, College Station, USA; ^7^Evidensia Small Animal Hospital Arnhem, Arnhem, The Netherlands; ^8^Hart van Brabant’ Waalwijk, Waalwijk, The Netherlands; ^9^Red Bank Veterinary Hospital, Tinton Falls, USA; ^10^College of Veterinary Medicine, Mississippi State University—Animal Health Center, Mississippi State, USA; ^11^Veterinary Neurology and Imaging of the Chesapeake—Towson, Towson, USA; ^12^Centro Clinico Veterinario Indautxu, Bilboa, Spain; ^13^Centre Hospitalier Vétérinaire Atlantia, Nantes, France; ^14^Center for Veterinary Specialty + Emergency Care, USA, Indianapolis, USA

Although hydrocephalus in small brachycephalic and toy breed dogs has been extensively reported and is well‐recognized, there is little information available about this condition in other breeds. This retrospective study aimed to report the clinical findings, magnetic resonance imaging (MRI) features, and outcome of medium‐to‐large breed dogs diagnosed with hydrocephalus. Medium‐to‐large breed dogs with internal hydrocephalus and the absence of other causes of encephalopathy on MRI were included in this retrospective multicentric study. Signalment, clinical history, neurological findings, MRI characteristics, treatment, and short‐term outcome were recorded. Thirty dogs with internal hydrocephalus met the inclusion criteria. Most affected dogs presented at a young age (median: 8.5 months, range: 2 months—9 years). Multiple breeds were affected with Border collies being overrepresented (8/29). The most common presenting complaints included ataxia (53%), vestibular signs (33%), and weakness (21%). Neuroanatomical localisation was variable with multifocal (11/30) and cervical spinal cord segments (8/29) found more frequently. MRI findings could be divided into ventriculomegaly (I) predominantly affecting the lateral ventricles (37%) and (II) affecting the entire ventricular system (63%). Additional features included syringomyelia (20/30) and supracollicular fluid accumulation (23/30). Twelve dogs were treated medically, and 14 dogs underwent surgery (13 by the placement of a ventriculoperitoneal shunt). Short‐term outcome was good in 3/12 dogs treated medically and 7/14 treated surgically. Unlike small breeds, many dogs in this study showed dilation of the entire ventricular system. Hence, obstruction of the lateral apertures should be considered as an important cause of hydrocephalus in medium‐to‐large breed dogs.

## (P 56)

### Pituitary tumors with neurological signs treated with 10x4GY radiotherapy

#### K. Beckmann, Dr. Med Vet, Dipl. ECVN^1^ , M. Körner, Dr. Med Vet^2^, V. Meier, Dr. Med Vet,Dipl. DACVR (Radiation Oncology)^2^

##### ^1^Department for Small Animals, Division of Neurology, Vetsuisse Faculty, University of Zurich, Zurich, Switzerland; ^2^Department for Small Animals, Division of Radiation Oncology, Vetsuisse Faculty, Zurich, Switzerland

Neurological signs of pituitary tumors have been associated with larger tumor sizes. Larger pituitary tumors have a less favorable prognosis if treated with surgery or radiation therapy (RT). Our objective was to evaluate pituitary/brain ratios, neuro‐imaging findings, and outcome in dogs with large pituitary tumors causing neurological signs treated with a standardized RT protocol. Dogs with imaging‐presumed pituitary tumors and neurological signs treated with 10x4Gy were retrospectively included. Neurological signs, pituitary/brain ratios, imaging findings and survival time (ST) were documented. Twenty‐two client‐owned dogs were included. Most common clinical signs were obtundation (91%), behavior changes (62%) and circling/pacing (41%). On initial imaging, 10/22 had suspected pituitary hemorrhage. Amelioration or complete normalization of all neurological signs was seen in 22/22 and 11/22 dogs, respectively. Median pituitary‐height‐to‐brain‐height ratio was 0.43 (range 0.33–0.55), median pituitary‐volume‐to‐brain‐ volume ratio 0.43 (range 0.01–0.11). Imaging follow‐up in 18 dogs showed a decrease in size in all but one dog at the time of their first imaging re‐check (median 6 months, range 0.5–20 months post RT). Pituitary hemorrhage decreased in 5/18, increased in 4/18 and newly occurred in 3/18 during the follow‐up period. Median overall ST was 922 days (95% CI: 71;1772), while median disease‐specific ST was not reached (mean 1556 days [range 66–2375]). Overall ST was not significantly different if pituitary/brain volume ratio (*P* = 0.65) or pituitary/brain height ratio (*P* = 0.79) was above the median. Large pituitary tumors with associated neurological signs responded favorably to 10x4Gy RT protocol. Suspected pituitary hemorrhage at initial diagnosis and during follow‐up was a common imaging finding.

## (P 57)

### Accuracy of a low‐ cost frameless neuronavigation system for brain biopsy in dogs

#### A. Kaczmarska, DVM MRCVS^1^ , R. Gutierrez‐Quintana, DVM MRCVS MVM DipECVN^1^ , M. Czopowicz, DVM PhD^2^ , E. Paran, DVM MRCVS^1^ ^,3^, S. Bouyssou, DVM MRCVS DipECVDI^1^ , G. Leblond, DVM DVSc DACVIM (Neurology) MRCVS^1,4^

##### ^1^Small Animal Hospital, School of Veterinary Medicine, College of Medical Veterinary and Life Sciences, Glasgow, UK; ^2^Division of Veterinary Epidemiology and Economics, Institute of Veterinary Medicine, Warsaw University of Life Sciences—SGGW, Warsaw, Poland; ^3^Langford Vets, Langford House, Langford, Bristol, UK; ^4^North Downs Specialist Referrals, Bletchingley, Surray, UK

Over the last decades, progress in advanced imaging and engineering led to the development of stereotactic neuronavigation techniques, improving diagnosis in patients with brain diseases. However, commercially available systems are cost‐prohibitive for most veterinary patients. The current study aimed to investigate the accuracy of a low‐cost frameless optical neuronavigation system. The study population consisted of 10 cadavers. CT images were uploaded into an open‐source software (3D Slicer) that was used for biopsy planning, neuronavigation, and data analysis. Commercial infrared cameras were used for optical tracking. Straight or angled skull‐mounted biopsy guides were used to establish targeting trajectories and facilitate the positioning of biopsy needles. Eight anatomical supratentorial targets were selected for each cadaver. Accuracy was evaluated by calculating the needle placement error (NPE) which was defined as the distance between the biopsy target and the needle tip coordinates. Overall, 76 observations were used to calculate a mean NPE of 2.1 ± 1.1 mm, with an upper NPE limit of 4.3 mm. NPE was not influenced by target depth or anatomical location. NPE was significantly higher with the angled base guide (2.5 ± 1.2 mm) than with the straight base (1.8 ± 0.8 mm) (*P* = 0.014). These results are comparable to the reported accuracy of various veterinary stereotactic systems. Considering the overall observed accuracy and the upper NPE limit, this low‐cost system could be used to biopsy supratentorial lesions over 10 mm in diameter. However, further studies are needed to validate the use of this system in vivo and in smaller lesions.

## (P 58)

### Surgical management of comminuted C2 fracture in an immature American Staffordshire terrier

#### A. Czerwik, PhD^1^ , A. Olszewska, Dr^1^, D. Farke, Dr, Dipl ECVN^1^ , J. Sobczynski, DVM^2^ , M. J. Schmidt, Prof, Dr, Dipl ECVN^1^

##### ^1^Department of Veterinary Clinical Sciences, Small Animal Clinic, Justus‐Liebig‐University, Giessen, Germany; ^2^Veterinary Clinic “Bemowo”, Warsaw, Poland

In veterinary medicine, cervical fractures are infrequently diagnosed. These injuries are often fatal or leave a poor prognosis prompting euthanasia. The reported treatment options include conservative management or surgical stabilization. This report describes the surgical treatment and follow‐up of an American Staffordshire terrier with complex C2 fracture. A 7‐month‐old female entire American Staffordshire Terrier, diagnosed with C2 fracture using x‐rays, was presented with a history of per acute onset of severe cervical pain and lack of improvement after initial conservative therapy. The neurologic examination showed symmetrical, non‐ ambulatory tetraparesis with increased spinal reflexes in all limbs and severe cervical hyperesthesia. The neurological localization was a C1‐C5 myelopathy. Computed tomography was performed revealing a comminuted fracture of the C2 vertebra with the cranial and dorsal displacement of caudal half part of the C2 vertebral body. Surgical stabilization of C2 was performed using a string of pearls plate with 2 screws on the left combined with “with 5 positive threaded Steiman Pins and PMMA on the contralateral side. Surgery was followed by cage rest with cast bandage and passive physiotherapy during hospitalization for 7 days. Further conservative therapy and weekly clinical controls including regular replacement of the cast bandage were performed. In the 1 month follow‐up the dog showed only mild proprioceptive ataxia and after 4 months follow‐ up the dog was unremarkable. The current case report describes the successful surgical management of the complex C2 fracture followed by return to unrestricted, pain‐free activities in the young dog.

## (P 59)

### Cranial vault bone changes associated to presumptive glioma in dogs

#### M. Pons‐Sorolla, DVM^1^ , E. Dominguez, DVM, MSc, PhD, Dipl. ECVDI, MRCVS^1^ , A. Suñol, DVM, Dipl ECVN, SFHEA, MRCVS^1^ , P. Montoliu, DVM, Dipl ECVN^1^

##### ^1^AniCura Ars Veterinaria Hospital Veterinari, Barcelona, Spain

The objective of this retrospective study is to describe cranial vault bone changes associated to suspected intracranial gliomas in dogs. Medical records of cases presented between September 2017 and December 2021 were reviewed. Dogs were included if a presumptive diagnosis of brain glioma was achieved, based on magnetic resonance imaging (MRI) features, consistent clinical history and progression. Magnetic resonance images were reviewed for cranial vault bone changes adjacent to the suspected glioma. Among 60 dogs with suspected intracranial glioma, 21 showed alterations in the bones adjacent to the lesion. Thinning of the bone was detected in 20 dogs, and 4 of them showed associated bone expansion. In one dog the adjacent bone was thickened. Bone changes were located in the temporal bone in 13/21 cases, frontal bone in 6/21, parietal bone in 1/21 and occipital bone in 1/21. In small animals, hyperostosis is the most common bone alteration secondary to intracranial neoplasia, frequently associated to meningioma. Gliomas producing adjacent bone erosion are occasionally described in humans. One case report described osteolytic bone changes adjacent to glioma in one dog. Despite the limited number of cases and the lack of a histopathological diagnosis, the results of our study suggest that calvarial thinning associated to gliomas is more common than previously recognized. This feature could be helpful in discrimination between glioma and other lesions such as infarcts or post‐ictal changes.

## (P 60)

### Canine degenerative myelopathy (CDM): A pilot comparative serum proteomic analysis of diseased and healthy animals

#### S. Tziola, MSc^1^ ^,2^, K. Psatha, MSc, PhD^3^ ^,4^, G. Stamatakis, MSc, PhD^5^ , R. D. Baka, DVM, MSc, PhD^6^ , M. Samiotaki, MSc, PhD^5^ , G. Orfanoudaki, MSc, PhD^3^ ^,4^, A. Triantafyllidis, MSc, PhD^2^ , Z. Polizopoulou, DVM, PhD, DipECVCP^6^ , M. Aivaliotis, MSc, PhD^3,4^

##### ^1^Laboratory of Biological Chemistry, School of Medicine, Faculty of Health Sciences, Aristotle University of Thessaloniki, Thessaloniki, Greece2 Laboratory of Genetics, Development and Molecular Biology, Department of Biology, Aristotle University of Thessaloniki, Thessaloniki, Greece; ^3^Functional Proteomics and Systems Biology (FunPAth), Center for Interdisciplinary Research and Innovation (CIRI‐AUTH), Thessaloniki, Greece; ^4^Institute of Molecular Biology and Biotechnology, FORTH, Heraklion, Greece; ^5^Institute for Bioinnovation, Biomedical Sciences Research Center “Alexander Fleming”, Vari, Greece; ^6^Diagnostic Laboratory, School of Veterinary Medicine, Faculty of Health Sciences, Aristotle University of Thessaloniki, Thessaloniki, Greece

Introduction: Canine degenerative myelopathy (DM) is a progressive, neurodegenerative disorder of the spinal cord that affects dogs older than 8 years. The aim of the study is to compare changes in proteome (presence/ relative abundance) of serum proteins between healthy and DM‐affected dogs, using a MS‐based approach. Methods: Bottom‐up proteomic analysis of six serum samples (3 from healthy large‐breed dogs and 3 from DM‐affected GSD dogs) was performed. Serum proteins precipitated in trypsin solution, followed by nLC‐ESI‐MS/MS analysis. Identification and relative quantification were performed using MaxQuant and Proteome Discoverer. Gene Ontology terms and pathway analysis of identified proteins were retrieved from UniProt and String databases, respectively. Results: In total, 226 proteins were isolated; 12 were solely detected in DM‐dogs. SAA1 and lysozyme (DM‐ unique proteins), have been associated with other neurodegenerative diseases (Alzheimer's disease and amyloidosis, respectively), and may considered as a potential diagnostic biomarker. Among the 54 proteins with different relative abundance between the two groups, 15 proteins were upregulated in DM‐group.GPX3, ApoE, ApoD, HP and the complement components C1qB and C1qC were more significant to DM. Functional analysis revealed that most proteins isolated from DM‐dogs are extracellular proteins and essential antioxidants, involved in immune system. Conclusion: Canine DM alters proteome profile in serum samples. Further investigation is needed in order to clarify the impact of epidemiology data and disease stage on our results.

## (P 61)

### Results of D‐dimers values in a population of dogs with idiopathic vestibular disease

#### S. Moya, DVM,MSc^1^ , N. Delgado, DVM^1^ , C. Maeso, DVM. Resident ECVN^2^ , T. Heredia, DVM^3^ , N. Martínez, DVM^3^ , A. Escudero, DVM^4^ , M. Lizarraga, DVM^4^ , M. Calvo, DVM^5^ , S. Rodenas, DVM, Specialist ECVN^31^

##### ^1^Animal Bluecare. Neurology Departament, Mijas Costa, Spain; ^2^Anicura Ars. Neurology Departament, Barcelona, Spain; ^3^Anicura Valencia Sur, Valencia, Spain; ^4^Aralar Veterinary Centre, Zaragoza, Spain; ^5^Anicura San Fermin, Pamplona, Spain

Idiopathic peripheral vestibular disease (IPVD) in dogs is a benign condition with acute onset of vestibular signs that in most cases resolves spontaneously within a few weeks and for which the cause remains undetermined. Inflammation and coagulation are closely related. D‐dimers are specific indicators of fibrinolysis and sensitive markers of systemic fibrinolytic activity. The purpose of this study was to investigate results of D‐dimers levels in a population of dogs with IPVD. Selection criteria included: neurological examination consistent with peripheral vestibular location, complete bloodwork (including normal thyroid profile and D‐dimers), abdominal ultrasound and thoracic radiographs, brain MRI with no lesions to explain vestibular signs and a minimum follow‐up period of 3 months. D‐dimers values were classified as normal (<0.25 mg/mL), and mild (0.25‐1) moderate (1‐2) and severe (>2) increase. Dogs that met these criteria but with other diseases that might increase values of D‐dimers were excluded. Fourty dogs fulfilled the inclusion criteria. The mean age was 12.5 years. Facial disfunction was present in 3 cases. Neurological sequelae (head tilt) and relapses were seen in 7 and 3 dogs respectively. The mean for the D‐dimers values was 1.19 (range 0.25‐7.54). D‐dimers were increased in 38/40 dogs. Results of D‐D were normal (2/40), mild (30/40), moderate (4/40) and severe (4/40) increase. Results of this study revealed an increase D‐dimers value in most of the cases. A non‐identified (by means of MRI) vascular or inflammatory disease might be a potential cause for some dogs with IPVD. Further studies are necessary.

## (P 62)

### Estimated prevalence of idiopathic and structural epilepsy in a large general population of cats undergoing mri for epileptic seizures

#### A. Knebel, Dr.^1^, J. Labruyère, Dr., DVM, CertVDI, DipECVDI, MRCVS, MEBVS^2^ , V. Johnson, Dr., BVSc, DVR, DipECVDI, MRCVS, MEBVS^2^ , K. Merhof, Dr., DipECVDI, MEBVS^1^ ,^2^, H. Volk, Dr., Prof., DipECVN, PhD, PGCAP, FHEA, MRCVS, MEBVS^13^

##### ^1^Small Animal Clinic, University of Veterinary Medicine, Hannover, Germany; ^2^VetCT, Cambridge, UK; ^3^Center for Systems Neuroscience Hannover (ZSN), Hannover, Germany

The reported prevalence of idiopathic epilepsy (IE) and structural epilepsy (SE) in cats varies based on factors such as study design, number of patients, and differences between primary care vs. referral centers' study populations. The aim of this study was to retrieve prevalence data of SE versus IE in a large general population of cats undergoing magnetic resonance imaging (MRI) for epileptic seizures. The MRI database, including patient signalment and clinical information, was provided by VetCT. Feline cases were reviewed and categorized as followed: No lesions detected (IE (6 months–7 years) and epilepsy of unknown etiology (>7 years), EuE) and structural lesions detected/SE. Lesions were further subclassified in intra‐axial and extra‐axial neoplasia, inflammatory‐, vascular‐, congenital‐, traumatic‐, metabolic/toxic‐ or degenerative diseases. Thiamin Deficiency and Hippocampal Necrosis were also recorded. Out of 432 MRIs, included in this study, 39% had SE, whereas 61% had no identified lesion (30% IE, 31% EuE). Of cats with SE, 25% had inflammatory diseases, 19% extra‐axial neoplasia, 11% metabolic/toxic etiologies, 10% congenital diseases, 9% lesions with unknown relevance, 7% intra‐axial neoplasia, 5% of each traumatic lesions and suspected Hippocampal Necrosis, 4% vascular etiologies, 3% Thiamin deficiency and 2% degenerative diseases. As this was an imaging‐based study, limitations are lack of histopathological diagnosis and clinical information was limited. The current study revealed that differently than formerly suspected nearly two out of three MRIs of cats presenting with epileptic seizures have no visible structural lesion in the brain.

## (P 63)

### Clinical presentation, diagnosis, treatment and outcome in three dogs with brucella discospondylitis in belgium: A retrospective, descriptive study

#### L. Ledeganck, DVM^1^ , N. Burger, DVM^1^ , K. Stee, DVM, DiplECVN^1^ , I. Cornelis, DVM, PhD, DiplECVN^1^ , E. Stock, DVM, PhD, DiplECVDI^2^ , F. Boyen, DVM, PhD^3^ , S. F. M. Bhatti, DVM, PhD^1^

##### ^1^Small Animal Department, Faculty of Veterinary Medicine, Ghent University, Merelbeke, Belgium; ^2^Department of Veterinary Medical Imaging and Small Animal Orthopedics, Faculty of Veterinary Medicine, Ghent University, Merelbeke, Belgium; ^3^Department of Pathobiology, Pharmacology and Zoological Medicine, Faculty of Veterinary Medicine, Ghent University, Merelbeke, Belgium

*Brucella canis* is a zoonotic disease that has an unknown prevalence in the Belgian canine population. Two mixed breed dogs and a Dutch Shepherd dog, aged 20, 25 and 58 months, respectively, had a history of either import from or travel to Eastern and Southern European countries. All dogs were presented 1–4 months after traveling with spinal hyperaesthesia of 1–6 months duration, followed by a stiff pelvic limb gait and lethargy. Neurological examination was normal, except for severe spinal hyperaesthesia in the cervical (1/3), thoracic (2/3) and lumbar (3/3) region. In one dog, multifocal discospondylitis was diagnosed radiographically by the referring veterinarian. In the two other dogs, computed tomography revealed multifocal discospondylitis in the cervical, thoracic, and lumbar region, as well as osteomyelitis of the left scapula and pelvis in one of these dogs. Complete blood count revealed a mild leukocytosis, monocytosis, neutrophilia and increased CRP (92 mg/L [<10 mg/L]) in one dog. Bacterial blood culture for *Brucella* spp. was positive in 2/3 dogs, while serology for *B. canis* antibodies was positive in all dogs with titers exceeding 1:50, 1:100 and 1:400 (reference <1:50). Two dogs showed clinical improvement following antibiotic treatment (enrofloxacin and doxycycline) and anti‐ inflammatory drugs over a period of 3 months. Despite the low zoonotic risk of *B. canis*, the third dog was euthanized. This case series highlights the importance of considering *B. canis* as a differential diagnosis in dogs with combined spinal hyperaesthesia, discospondylitis and osteomyelitis, particularly when traveling from endemic regions.

## (P 64)

### Phenotypic characterization of idiopathic epilepsy in Irish setters

#### M. Plonek, DVM, PhD^1^ , K. Santifort, DVM^1^ ,^2^, M. M. Diaz‐Espineira, DVM, PhD^3^ , Q. E. M. Stassen, DVM^3^ , P. A. J. Leegwater, MSc, PhD^3^ , P. J. J. Mandigers, DVM, PhD^13^

##### ^1^Evidensia Referral Hospital, Arnhem, Netherlands; ^2^Evidensia Referral Hospital, Waalwijk, Netherlands; ^3^Department of Clinical Sciences of Companion Animals, Faculty of Veterinary Medicine, Utrecht University, Utrecht, Netherlands

Idiopathic epilepsy (IE) with a suspected genetic background has been reported in a number of dog breeds, including the Irish Setter. The aim of this study is characterizing the phenotype of IE in Irish Setters from the Netherlands. The dogs were either seen after referral or owners were contacted via the Dutch Irish Setter Club and requested to fill in a questionnaire and send in video footage of their dog during an attack. The data was assessed retrospectively using descriptive statistics. A pedigree analysis of 10 affected Irish Setters was performed. Thirty‐six Irish Setter dogs with a tier I confidence level for the diagnosis of IE according to the International Veterinary Epilepsy Task Force were included in the study. Twenty‐one (58%) dogs were male. The median age of seizure onset was 36 months (range: 3.5–108 months). Cluster seizures were reported in 19 (53%) dogs. Most owners reported pre‐ (55%) and post‐ictal (97%) signs in their dogs. Generalized seizures were reported in all dogs (100%). Focal seizure activity was noticed by owners in 12 dogs (33%) prior to a generalization. In the majority of the dogs (*n* = 31), the ictus lasted <5 min. The pedigree analysis of the 10 dogs revealed unaffected parents, suggesting an autosomal recessive inheritance pattern. In summary, we report IE in a Dutch population of Irish Setter dogs. A strong genetic component is suspected, and the high percentage of cluster seizures supports the implementation of early and rational anti‐epileptic treatment in this breed.

## (P 65)

### Intraspinal spirocercosis, treatment protocol and outcome in 8 dogs

#### N. Asiag, DVM^1^ ,^2^, S. Yodonver, BSc^1^ , M. Ruggeri, DVM^1^ , G. Baneth, BSc, DVM, Ph.D., Dipl ECVCP^1^ , L. Konstantine, B.A., M.A.^1^ , O. Chai, BSc, DVM, Dipl ECVN^2^ , M. H. Shamir, BSc, DVM, Dipl ECVN^1^

##### ^1^Koret school of veterinary medicine, Veterinary Teaching Hospital, Hebrew University of Jerusalem, Rehovot, Israel; ^2^Veterinary Specialist Referral Tipul Nimratz, Ben Shemen, Israel

Intraspinal migration of *Spirocerca lupi* is an important cause of per‐acute progressive spinal dysfunction in endemic areas. Once entering the cord, the migration causes severe parenchymal damage, microscopic hemorrhages, and blood spinal cord barrier (BSCB) disruption. Ivermectin prevent the transmission of electrical impulse in the muscle and nerves of invertebrates by amplifying the glutamate effects on the specific gated chloride channel. Ivermectin is the drug of choice for treating esophageal *S. lupi* and is also used routinely as a preventative treatment. This study evaluated the efficiency and safety of frequent ivermectin administration (FIA) protocol as a treatment against spinal spirocecosis. Our preliminary study demonstrated BSCB disruption around the nematode and neighboring spinal segments only assuring the safety of the protocol. Methods: A retrospective study on dogs diagnosed with ISSC which received the FIA protocol including 400 μg/kg, SC, daily for 3 times and once a week for another 6 times. Short and long‐term follow‐up outcomes are available. Results: Eight dogs completed the inclusion criteria. 7/8 presented with neurological deficit and one with cervical pain. Seven dogs improved and recovered ambulation and one dog was euthanized. Six of the recovered dogs were still ataxic on the last follow‐up examination 6–18 months from diagnosis and treatment. No adverse effect of the drug was noted. Conclusion: the frequent administration of ivermectin at a dose of 400 μg/kg is safe and was found to be effective in stopping the nematode and preventing further deterioration of the neurological status.

## (P 66)

### Vertebral giant cells tumors in a cat

#### C. Pappalardo, DVM^1^ , S. Gasparini, DVM, ECVP Dipl.^2^, V. Imburgia, DVM, Master of oncology^1^, G. La Cava, Dvm, ECVDI Dipl^3^

##### ^1^Clinica Veterinaria Primavera, Palermo, Italy; ^2^Laboratorio d'analisi San Marco, Veggiano, Italy; ^3^Istituto Veterinario di Novara, Granozzo con Monticello, Italy

A 3‐year‐old female spayed domestic short hair cat was presented with a 3 months history of progressive pelvic limb weakness, back pain, and bladder dysfunction. The neurological examination showed marked ambulatory paraparesis, proprioceptive deficits in both hind limbs, and detrusor arefexia with sphincter hypertonus. Neurological findings were consistent with a T3‐L3 localization of the lesion. No significant abnormalities were identified in the complete blood cell count and serum biochemistry, FIV‐ Felv test was negative. TC study and MRI study were performed. The studies showed an expansive osteolytic lesion, oval in shape, with defined margins originating from the right caudal articular facet of L2 associated with invasion of the vertebral canal and massive spinal cord compression. The lesion was compatible with a primary spinal tumor of probable bone origin. EN BLOC resection and decompression surgery were performed. The histopathologic findings were consistent with spinal giant cell tumors (GCT). The patient presented a resolution of the symptoms in about 15 days. Four months after the surgery the cat was doing well. In human literature, a giant cell tumor is a rare, invasive benign bone tumor, which is found in the long bone rarely in the spine, total resection in the early stage is the best treatment. This is the first reported case of a giant cell tumor of bone localized in the spine in a cat. This tumor, with a potentially good outcome, should be considered as a differential diagnosis for vertebral bone tumors also in cats.

## (P 67)

### L‐2‐hydroxyglutaric aciduria (L‐2‐HGA) in Yorkshire terrier—more than one genetic mutation—A case report

#### M. Cedzo, MVDr.^1^, M. Rosati, DVM, PhD, Dipl. ACVP^2^ , A. Caine, MA, VetMB, Dipl. ECVDI, CertVDI^3^ , K. Matiasek, DVM, DrMedVetHabil^2^ , V. Palus, Dipl. ECVN^14^

##### ^1^Neurovet, Trencin, Slovakia; ^2^Ludwig‐Maximilians‐Universitaet, Muenchen, Germany; ^3^Dick White Referrals, Cambridgeshire, UK; ^4^University of Veterinary Sciences, Brno, Czech Republic

L‐2‐hydroxygluratic aciduria (L‐2‐HGA) is a disease caused by inborn error of intermediary metabolism affecting humans and various breeds of dogs. A genetic origin of this autosomal recessive metabolic disorder is confirmed, with a specific codon mutation described in Yorkshire terriers. Here, we report the case of a 6‐year‐old Yorkshire terrier with a history of epileptic seizures, progressive ataxia, generalized tremors and diffuse hyperesthesia on palpation. Magnetic resonance imaging revealed diffuse symmetric hyperintensity of cerebral cortical and cerebellar gray matter on T2 weighted sequences, and bilaterally symmetrical T2 weighted hyperintense foci located in thalamus and brainstem; imaging findings considered consistent with L‐2‐HGA. Euthanasia was elected several months after presentation due to neurological deterioration and development of aggressive behavior. Brain histology showed a degenerative spongy polioencephalopathy consistent with L‐2‐HGA in Staffordshire bullterriers. However, the commercially available DNA test on brain tissue for L‐2‐HGA specific for Yorkshire terrier was negative. Unfortunately, due to lack of genetic material preserved, further analysis of DNA was not possible. However, this case report supports the existence of multiple L2HGDH gene mutations causing L‐2‐HGA in Yorkshire terriers, as it was reported in humans. Further research is warranted to detect novel genetic mutations of L2HGDH gene in Yorkshire terriers.

## (P 68)

### Intracranial foreign body removal (pellet) by fluoroscope‐assisted craniotomy

#### S. Moya, DVM, MSc^1^ , N. Delgado, DVM^1^ , V. Moya, DVM^2^ , J. J. González, DVM^3^ , S. Rodenas, DVM^1^

##### ^1^Animal Bluecare Hospital, Mijas Costa, Spain; ^2^Dr. Moya Clinic, Torremolinos, Spain; ^3^VET&VET Clinic, Sevilla, Spain

Intracranial foreign body (pellet) as a result of a shot has not been described in the veterinary literature. The identification of their radiological features is mandatory for proper diagnosis. As occurs with other foreign body in other organs, surgical removal and directed therapy for clinical signs (seizures, edema) should be considered the treatment of choice. A clinical case of a mixed breed dog with forebrain signs and x‐ray, magnetic resonance imaging and computed tomography findings compatible with intracranial foreign body (bullet) with edema in the brain is described. An exploratory rostro‐tentorial craniotomy with durotomy was performed, allowing the drainage of purulent content and the extraction of a bullet foreign body from the cerebral parenchyma. Antibiotic and antiepileptics drugs treatment was instituted and the patient was discharged without recurrence of neurological deficits other than quarterly seizures. Six months later the patient remains stable, only with absence menace response in both eyes manifesting isolated seizures that are controlled with medicati. This is the first case in veterinary literature in which a bullet has been surgically extracted from the brain of a dog fluoroscope‐assisted with long‐term outcome described. Observed changes in clinical signs (seizures, depress mental status, and absence menace response) and the history were fundamental to establish the pre‐surgical diagnosis of a foreign body in the brain.

## (P 69)

### Encephalopathy in an european brown bear cub (ursus arctos arctos)—A case report

#### S. Werner, MVDr.^1^, K. Matiasek, DVM, DrMedVetHabil^2^ , A. Caine, MA, VetMB, Dipl. ECVDI, CertVDI^3^ , M. Belak, MVDr.^4^, H. Danielova, MVDr.^5^, B. Tam, Ing.^6^, V. Palus, Dipl. ECVN^17^

##### ^1^Neurovet, Trencin, Slovakia2 Ludwig‐Maximilians‐Universitaet, Muenchen, Germany; ^3^Dick White Referrals, Cambridgeshire, UK; ^4^Veterinary Clinic Zoovet, Prievidza, Slovakia; ^5^Laboklin, Bratislava, Slovakia; ^6^National Zoological Garden, Bojnice, Slovakia; ^7^University of Veterinary Sciences, Brno, Czech Republic

Neurological diseases in bears are sparsely reported in literature. This case report describes encephalopathy in young European brown bear cub with magnetic resonance imaging (MRI) and post‐mortem description of the brain. Approximately 10‐month‐old European brown bear cub was found in a stupor. After the capture it was treated with fluid therapy and glucocorticosteroids at the rescue centre. The bear improved and started presenting signs of vestibular ataxia, falling to right, disorientation, circling to right, possible blindness and anosmia. After 2 weeks it was referred for lack of further improvement. Neurolocalisation of a diffuse intracranial lesion was made on the base of video consultation. MRI revealed marked brain atrophy with symmetrically widened sulci, and lateral ventriculomegaly including marked symmetric distension of the olfactory recesses. Additionally, there was a symmetric band of T2 weighted hyperintensity at the gray matter/white matter junctions in the frontal and occipital lobes. Cerebrospinal fluid (CSF) examination revealed elevated nucleated cell count—10/μL and mononuclear pleocytosis. Multiple infectious agents were ruled out. Treatment included glucocorticosteroids and thiamine supplementation. Despite mild improvement blindness was still present. This was incompatible with bear's welfare, and it was euthanised. Neuropathological examination confirmed gross MRI changes and revealed hippocampal dysplasia, multifocal cerebral cortical necrosis and a predominantly paravermal cerebellar cortical degeneration. Hence, the cub was affected by a malformative brain disease, possibly precipitated by metabolic, toxic or vascular factors. This case report is a valuable addition to sparse knowledge in the field of wildlife veterinary medicine with full diagnostic work‐up and post‐mortem findings.

## (P 70)

### Mycotic discospondylitis in a german shepherd: Clinical presentation and usefulness of antimicogram

#### S. Okonji, DVM^1^ , F. Dini, DVM^1^ , S. Del Magno, DVM, PhD^1^ , V. Cola, DVM^1^ , G. Gandini, DVM, PhD, DECVN^1^

##### ^1^University of Bologna, Department of Veterinary Medical Sciences, Bologna, italy

An 8‐year‐old spayed female German shepherd was presented with a 2‐week history of severe neck pain, lameness of the left forelimb, followed by progressive pelvic limb proprioceptive ataxia and ambulatory paresis. Neurological examination showed left lateralized C6–T2 myelopathy. Blood exams revealed a mild inflammation. MRI showed multiple lytic changes in the intervertebral discs and a left ventrolateral C6‐C7 compressive myelopathy due to a suspected empyema. A mycotic discospondylitis was suspected. Urinalysis showed fungal hyphae and a definitive diagnosis of Aspergillosis was determined by urine culture. Fluconazole 5 mg/kg twice daily and analgesic therapies was started. Clinical signs significantly improved, and follow‐up urinalysis showed no fungal hyphae. Three months later, hematuria was detected. Urinalysis showed fungal hyphae. Antimicogram was performed and revealed a high minimal inhibitory concentration for fluconazole and low for itraconazole. A therapy with itraconazole at 5 mg/kg BID was then started while the therapy with fluconazole was suspended. The condition of the dog significantly improved with almost complete disappearance of pain and ataxia. After 16 months from presentation, the dog presented a severe non‐deambulatory paraparesis. Antifungal therapy had been discontinued 2 weeks earlier by the own decision of the owner without any medical indication. MRI revealed severe right side compressive T11‐T13 myelopathy caused by high contrast enhancement material. Euthanasia was performed due to a rapid worsening of neurological signs. In conclusion, urine culture and antimicogram may represent a useful tool to guide the clinician in the choice of the correct antifungal therapy in dogs diagnosed with fungal discospondylitis.

## (P 71)

### Oligodendroglioma in an 8‐month‐old boxer

#### E. Lichtenauer, DVM^1^ , K. Santifort, DVM^1^ , M. Beukers, DVM, Diplomate ECVDI^1^ ,K. Matiasek, DVM, PhD, Diplomate ECVN^2^ , N. Bergknut, DVM, PhD, Diplomate ECVN^1^

##### ^1^Evidensia Dierenziekenhuis Hart van Brabant, Waalwijk, The Netherlands; ^2^Institute for Animal Pathology, University Munich, Munich, Germany

Structural causes are included in the differential diagnosis list for young dogs presented for epileptic seizures. Neoplastic disease at early age is reported far less commonly than for instance anomalous or inflammatory structural disorders. An 8‐month‐old male Boxer dog was referred for acute‐onset epileptic seizures and abnormal behavior. Hematology, biochemistry, electrolytes, C‐reactive protein and pre‐prandial bile‐acids were within reference range. MRI of the brain showed a large, ill‐defined contrast enhancing intra‐axial mass in the right piriform/ temporal lobe, with an intraventricular component and signs of intralesional hemorrhage. The ependyma was irregularly thickened and strongly contrast‐enhancing. There were findings compatible with obstructive hydrocephalus. An inflammatory/infectious lesion and neoplasia were considered. The dog died despite medical treatment. Histopathology revealed an extremely cellular mass, consisting of mainly disorganized streams, ribbons and nests of tightly packed, markedly atypical pseudocohesive neuroglial type cells. The cells stained negative for GFAP and synaptophysin, while a majority was positive for cytoplasmic marker MAP2 and nuclear marker OLIG2. More than 50% of the cells expressed Ki‐67 antigen. A highly proliferative anaplastic oligodendroglioma was diagnosed. Boxers are predisposed for oligodendrogliomas. In a larger cohort study, the age span reported for dogs diagnosed with oligodendroglioma ranged from 2 to 14 years. However, two cases of dogs of 5 and 15 months old with an oligodendroglioma in the brainstem and cerebrum respectively, were reported. This case underlines that although neoplastic disease is more likely to occur in older dogs, it should also be included in the differential diagnosis list of young dogs with epileptic seizures.

## (P 72)

### Global hypoxic‐ischaemic brain injury: Clinical presentation and outcome in five dogs and two cats

#### A. Crawford, BVM&S BSc PhD DipECVN MRCVS^1^ , C. Danciu, DVM(Hons) MRCVS^1^ , D. Yaffy, BVetMed MRCVS^2^

##### ^1^Clinical Science and Services, Royal Veterinary College, Hawkshead Lane, AL9 7TA, North Mymms, United Kingdom; ^2^Pathobiology and Population Sciences, Royal Veterinary College, Hawkshead Lane, AL9 7TA, North Mymms, United Kingdom

Global hypoxic‐ischaemic brain injury results in varying degrees of neurological dysfunction, dependent on the duration and extent of the inciting hypoxic‐ischaemic insult. Limited data exists in veterinary medicine to guide prognostication. We describe seven clinical cases with global hypoxic‐ischaemic brain injury including their presenting neurological deficits, clinical progression and outcome. Five patients (four dogs and one cat) had a cardiac and/or pulmonary arrest under general anesthesia and one cat had a cardiopulmonary arrest during investigations of hypertrophic cardiomyopathy and fulminant left sided congestive heart failure; each was promptly resuscitated and showed progressive neurological improvements with no to minimal residual neurological deficits (predominantly visual deficits) reported (median follow up 82 days [range 19–1456]). The seventh case presented to the hospital in cardiopulmonary arrest following laryngeal obstruction by a foreign body. Spontaneous circulation was recovered after 22 min of cardiopulmonary resuscitation including external defibrillation. Electroencephalography revealed discontinuous background activity of 10–15 uV, consistent with concurrent medetomidine sedation. Despite prolonged supportive and intensive nursing care the dog remained blind, disorientated, ambulatory tetraparetic with vestibular ataxia and was euthanised 58 days following presentation. Histopathological examination of the brain confirmed diffuse severe cerebral and cerebellar cortical necrosis. Duration of the hypoxic‐ischaemic insult and rate of subsequent neurological recovery may provide indications of the likelihood of functional recovery in dogs and cats with global hypoxic‐ischaemic brain injury. Studies to develop electroencephalographic prognostic indicators and plasma biomarkers are future goals.

## (P 73)

### On a case resembling white matter vanishing syndrome in a young Yorkshire Terrier

#### T. Neuhaus, Med Vet^1^, I. Carrera, DVM, DipECVDI^2^ , M. Hilbe, DVM, DiplECVP, FVH^3^ , H. Marti, DVM, Dr. sc. nat.^3^, L. Golini, DVM, DiplECVN^1^

##### ^1^Section of Neurology and Neurosurgery, Small Animal Clinic, Vetsuisse Faculty, University of Zurich, Zurich, Switzerland; ^2^Vet Oracle Teleradiology, Norfolk, UK; ^3^Section of Pathology, Small Animal Clinic, Vetsuisse Faculty, University of Zurich, Zurich, Switzerland

Background: Leukoencephalopathies are rarely described in dogs. This report describes the results of clinical, MRI, and histopathological examination of a young Yorkshire Terrier with slow progressive, multifocal signs of a leukoencephalopathy, resembling white matter vanishing disease in people. Case presentation: A 6‐months‐old, female, entire Yorkshire Terrier was presented due toprogressive forebrain and cerebellar symptoms. The dog repeatedly extended all four limbs and had a spastically erected tail when the neck was manually extended. When the neck was flexed, he crouched all four limbs. Cerebellar ataxia, behavioral changes and absent menace response were also observed. Initial MRI revealed almost diffuse symmetric lesions affecting the white matter of the forebrain and cerebellum, homogenously hyperintense in T2‐weighted images. The gray matter was mostly spared. Twelve months later, MRI showed marked reduced volume and almost complete disappearance of the previously affected white matter. MR Spectroscopy showed increased lactate concentration in parenchyma of affected area, confirmed by its CSF quantification. Postmortem pathological examination revealed bilateral vacuolizing degeneration of the white matter in the central nervous system, severe and diffuse, with partially extensive necrosis. These findings were suggestive of a white matter vanishing like syndrome with possible mitochondrial encephalopathy. Conclusion: This report presents the case of a young Yorkshire Terrier with a newly recognized neurodegenerative, mitochondrial disease predominantly affecting the white matter. Similar reports are missing in veterinary medicine, but comparable diseases might be found in human medicine, such as Kearns‐Sayer's syndrome.

## (P 74)

### Diffusion tensor imaging assessmentof pyramidal pathways in the course of Syringomyelia in Cavalier King Charles Spaniel

#### A. Banasik, PhD student, DVM^1^ , K. Owsinska‐Schmidt, PhD student, DVM^1^ , A. Zimny, Professor, MD^2^ , M. Wrzosek, Professor, DVM^1^

##### ^1^Department of Internal Diseases with a Clinic for Horses, Dogs and Cats, Faculty of Veterinary Medicine, Wroclaw University of Environmental and Life Sciences, Poland, Wroclaw, Poland; ^2^Department of General and Interventional Radiology and Neuroradiology, Wroclaw Medical University, Poland, Wroclaw, Poland

The aim of this study was to assess pyramidal pathways damage in the course of syringomyelia using diffusion tensor imaging (DTI) sequence. Forty Cavalier King Charles Spaniel dogs were qualified for the research. The animals were divided into three groups based on the radiographic changes visible on MRI: dogs with syringomyelia (SM group, 16 dogs), dogs with central canal dilatation (CCD group, 9 dogs), and dogs without radiological symptoms (nonSM group, 15 dogs). All animals underwent the same study protocol that included a clinical and neurological examination followed by MRI examination. DTI was performed with a 1.5 Tesla magnetic resonance scanner (Philips, Ingenia). Two DTI parameters: fractional anisotropy (FA) and apparent diffusion coefficient (ADC) were measured. Measurements of FA and ADC values were made by drawing regions of interest (ROIs) at the level of capsula interna (ROI‐1), pyramidal tract in ventral part of the pons (ROI‐2), at the junction of medulla oblongata and spinal cord (ROI‐3), and in the middle of C2 (ROI‐4). Image post‐processing was done using the Fiber Track package (Philips Ingenia workstation). Statistical analysis showed significant differences in DTI parameters between the nonSM and SM group in FA values in ROI‐3 (*P* = 0.0057) and ROI‐4 (*P* = 0.0024), as well as CCD and SM group in FA values in ROI‐3 (*P* = 0.034) and ROI‐4 (*P* = 0.0032) also in ADC values in ROI‐4 (*P* = 0.02). Findings suggest that the most severe damage to pyramidal pathways in the course of SM occurs at the level of ROI‐3 and ROI‐4. DTI values measurements may provide more objective spinal cord microstructure.

## (P 75)

### Polyneuropathy associated with primary erythrocytosis (*Polycythaemia vera*) in a cat

#### J. Galer, BVetMed MRCVS^1^ , P. Ibarrola, DSAM DipECVIM‐CA MRCVS^2^ , E. Royaux, DVM DipECVN MRCVS^1^

##### ^1^Davies Veterinary Specialists, Hitchin, United Kingdom; ^2^The Ralph Veterinary Referral Centre, Marlow, United Kingdom

Polyneuropathy associated with polycythaemia vera has only been reported in humans and is considered rare; typically, neuromuscular dysfunction occurs as a late complication of polycythaemia but it has also been reported as the primary presenting complaint. A 22‐month‐old, male neutered domestic longhair cat presented with a 2‐month history of progressive pelvic limb weakness and muscle atrophy, a neurological examination was consistent with neuromuscular dysfunction. A manual PCV was 73%, serial measurements following intravenous fluid therapy remained high. Investigations for secondary causes of erythrocytosis were unremarkable so a diagnosis of primary erythrocytosis was made; a serum erythropoietin measurement returned normal 3 weeks after presentation supporting this diagnosis. Electromyography showed spontaneous activity within all tested muscles of the pelvic limbs, distal muscles exhibited more activity compared to proximal muscles. Motor nerve conduction velocity for the sciatic‐tibial nerve was prolonged and compound muscle action potentials measured at the hip and stifle were reduced; these results indicated demyelination and axonal loss. Phlebotomy achieved a reduction of the PCV to 52% and oral hydroxyurea was started 2 weeks later. An improvement in clinical signs was seen 3 weeks after discharge, this continued until the cat was normal at 4 months. Fluctuations occurred in the PCV necessitating adjustments in the dose of the hydroxyurea, but no relapse of the neuromuscular signs occurred during the 12 month follow up period. Primary erythrocytosis should be considered as a differential in cats presenting with neuromuscular dysfunction. Successful management of the erythrocytosis resulted in a resolution of the clinical signs.

## (P 76)

### Spinal cord compression secondary to extramedullary hematopoiesis in two dogs: Imaging and histopathological findings and surgery

#### T. Heredia, LV^1^ , A. Costas, LV^1^ , V. Cervera, LV, DipACVR, DipECVDI^1^ , S. Ortiz, LV^1^ , S. Ródenas, LV, MRCVS, DipECVN^1^

##### ^1^ AniCura Valencia Sur, Valencia, Spain

Extramedullary hematopoiesis (EMH) is a blood cell formation and growth outside bone marrow that can occur in the liver and spleen and less frequently in other tissues. We present two patients with EMH leading to spinal cord compression (SCC). Extradural EMH and MRI findings has only been described in one dog without underlying pathology. Two patients (9‐year‐old German Shepherd and 11‐year‐old Doberman) were referred with a history of ambulatory paraparesis progressing to non‐ambulatory paraparesis. Neurological examination revealed a T3–L3 myelopathy in both patients. Magnetic resonance imaging (two patients) and CT (second patient) were performed. In the first case, the MRI showed five hemorrhagic extradural lesions producing severe dorsal SCC throughout T12–T13–L1. Owners decided euthanasia and histopathology revealed EMH. In the second case, both MRI and CT revealed a hemorrhagic extradural lesion dorsal to the spinal cord generating severe SCC at T12–T13. Dorsal laminectomy was conducted, with the material being analyzed, resulting in EMH. The patient recovered ambulation after surgery and presented mild ambulatory paraparesis after 12 months. EMH causing SCC has been reported infrequently in humans mostly associated with myeloproliferative disorders. To the author ´s knowledge only one case of EMH has been reported in dogs with MRI, we present two cases with MRI and one case with CT findings of EMH, both confirmed by histopathology. In our case and in the case previously reported, decompressive surgery showed good results with improvement of neurological signs in long term

## (P 77)

### Application of diffusion tensor tractography in suspected intracranial neoplasms in 30 dogs and 3 cats

#### T. Heredia, LV^1^ , A. Costas, LV^1^ , V. Cervera, LV, DipACVR, DipECVDI^1^ , S. Ortiz, LV^1^ , S. Ródenas, LV, MRCVS, DipECVN^1^

##### ^1^AniCura Valencia Sur, Valencia, Spain

Magnetic resonance imaging (MRI) is the technique of choice to evaluate diseases affecting cerebral white matter (CWM) in humans and animals. Conventional MRI does not allow visualization of the CWM fiber tracts and connectivity of the brain. Diffusion Tensor Tractography (DTT) is a noninvasive imaging technique that allows to identify the fiber tracts (FT) of CWM in vivo. To the author's knowledge no studies have been reported of DTT in small animals with suspected brain neoplasms. The aim of these study is to assess DTT role in intracranial neoplasms in dogs and cats. Thirty‐three animals were enrolled in this study (30 dogs and 3 cats). The mean age was 9.7‐years. The following MRI sequences were performed in all cases: pre and postcontrast T1W, T2W, DWI/ADC, FLAIR, T2* and DTT imaging. Extra‐axial masses (EAM) and intra‐axial masses (IAM) accounted 51.5% and 48.5% of cases, respectively. Conventional MRI showed hemorrhage 33.3% of cases. DTT revealed total or partial fiber disruption in 36.3% of EAM and 68.75% of IAM. Displacement without FT disruption occurred in 15.1% of cases (all of them EAM). Total fiber disruption was present in the hemorrhagic areas. In this study, CWM total or partial FT disruption occurred more frequently in IAM suggesting more involvement or infiltration of CWM. Hemorrhage was correlated with fiber disruption. DTT is often applied in human medicine for characterization of intracranial tumors, their extent and surgery planification. Further studies are needed to determine DTT role in intracranial neoplasms in veterinary medicine.

## (P 78)

### “How deep is your love”—The socioeconomic burden of intervertebral disc herniation in dogs

#### S. Grieder, Med Vet^1^, B. Friker, Med Vet^2^, V. Stein, Prof, Dr, Med Vet, PhD, Dipl ECVN^1^

##### ^1^Division of Clinical Neurology, Vetsuisse Faculty, University of Bern, Bern, Switzerland; ^2^Veterinary Public Health Institute, Vetsuisse Faculty, University of Bern, Bern, Switzerland

Intervertebral disc herniation (IVDH) is a trending disease due to raising popularity of chondrodystrophic dog breeds. The care of affected dogs takes a great financial and emotional commitment on the part of the owner while information on long‐term costs is missing. Aim of the study was to assess the socioeconomic burden of IVDH on the owner. We hypothesized that higher severity of neurological signs implies with a higher financial burden. Patient data (2017–2020) of dogs with different severity of neurological signs attributable to IVDH confirmed by MRI were retrospectively analyzed. An online questionnaire was sent to their owners that assessed costs caused by the dog's IVDH generated before presentation, in hospital and after discharge from hospital. A total of 224 dogs medical records (129 males, 95 females) fulfilled the inclusion criteria of which 76 dog owners completed the online survey. The median costs for IVDH generated before presentation added up to CHF 500 (range: CHF 55–5000), in hospital to CHF 3001 (range: CHF 750–5842) and after release from hospital to CHF 350 (range: CHF 36–3500). Higher severity of neurological signs correlated with higher costs during hospitalization. No significant difference was found for the costs generated before presentation or after hospital discharge in different severity of neurological signs due to IVDH. IVDH has a high socioeconomical impact on pet owners. The results of this study provide the basis for a more propriate estimation of the life‐long costs implicated with IVDH.

## (P 79)

### Upregulation of myeloperoxidase in hypertensive cats indicates oxidative stress and neuroinflammation beyond candidate areas of hypertension‐induced vascular accidents

#### L. Reuter, DVM^1^ , S. Bertram, DVM DECVN^2^ , A. Fischer, DrMedVetHabil DACVIM (Neurology) DECVN^1^ , L. Matiasek, DrMedVet DECVN^3^ , M. Rosati, PhD DACVP^1^ , K. Matiasek, DrMedVetHabil AM‐ECVN^1^

##### ^1^Ludwig‐Maximilians‐Universitaet, Munich, Germany; ^2^Cave Vet Referrals, West Buckland, UK; ^3^Frontier Kleintierspezialisten, Vaterstetten, Germany

Myeloperoxidase (MPO) is an inflammatory enzyme that is upregulated in various ischaemia‐reperfusion syndromes. Targeted by specific drugs, its downregulation proved effective to attenuate brain damage in vascular encephalopathies in people and laboratory animals. As arterial hypertension is the most prevalent risk factor for vascular accidents in cats, this study was performed to investigate the MPO pattern in brains of cats with hypertensive encephalopathy (HE). Brains of deceased cats with confirmed HE (11/20 with and 9/20 without infarcts) were immunohistochemically investigated for cellular expression of MPO and compared to 10 control cats and the expression pattern of microglial activation marker Iba1. Previously identified hot spot areas of parenchymal damage were thereby compared to less affected areas as an internal expression control. Already cats with mild HE and no evidence of vascular accident showed a significantly increased MPO expression (*P* < 0.05) throughout all investigated areas. Neither progression of HE nor the occurrence of infarcts lead to further increase of MPO. MPO did further not always correlate to the Iba1 expression that was most extensive in vascular accidents. In contrast to Iba1, MPO expression did not show age‐related effects. MPO indeed appears to be upregulated at early stages of feline HE. Targeting MPO by blocking agents might interfere with the neuroinflammatory consequences of HE‐induced ischaemic‐reperfusive and pervasive events.

## (P 80)

### Null cell lymphoma causing intracranial hypertrophic pachymeningitis in a dog

#### M. Madden, BVM BVS PhD MRCVS^1^ , K. Marioni‐Henry, DMV PhD DipACVIM(neurology) ECVN SFHEA MRCVS^1^ , A. Malbon, BSc (hons) BVSc Dr. Med Vet PhD DipECVP MRCVS^1^ , N. Israeliantz Gunz, LdoVet MRCVS^1^ , T. Schwarz, MA Dr. Med Vet FRSB DipECVDI DACVR DVR FRCVS^1^ , A. Suñol, Lda Vet DipECVN MRCVS^1^

##### ^1^Hospital for Small Animals, Royal (Dick) School of Veterinary Studies, University of Edinburgh, Edinburgh, UK

Hypertrophic pachymeningitis (HP) is a rare magnetic resonance imaging (MRI) finding in dogs. In humans, HP can be idiopathic or secondary to a number of immune‐mediated, infectious or neoplastic diseases. Within the veterinary literature, descriptions of this imaging finding are limited to a single study of six dogs with suspected intracranial idiopathic HP, the majority of which responded favorably to immunosuppressive treatment. In this case report, we describe the neurological presentation, infectious disease testing, cerebrospinal fluid analysis and MRI findings in a 2‐year‐old female neutered pug diagnosed with suspected intracranial idiopathic HP. Immunosuppressive treatment resulted in a short period of clinical improvement, followed by relapse of the neurological signs within 6 weeks of the initial presentation. A repeat MRI study at the time of deterioration revealed progression of the pachymeningeal thickening. Alternative immunomodulatory strategies were unsuccessful and due to progressive neurological deterioration, the patient was euthanised. On post‐mortem examination, diffuse thickening of the dura mater was observed. Histological examination was consistent with a meningeal large cell lymphoma. Immunohistochemistry was negative for both B and T cell markers, whilst PCR for antigen receptor rearrangement (PARR) identified a clonal B‐cell receptor rearrangement. This case report represents the first description of meningeal lymphoma as a cause of HP in a dog. Hypertrophic pachymeningitis is a rare imaging finding in dogs and neoplastic aetiologies should be considered. Surgical biopsy may be indicated in patients with an unusual signalment, or those which demonstrate an inadequate response to immunosuppressive treatment.

## (P 81)

### Sacro‐caudal dysgenesis in two boxer dogs: Clinical presentation, diagnostic investigations and outcome

#### D. Dell'Apa, Resident ECVN^1^ , M. Fumeo, PhD^1^ , A. Volta, Associate Professor^1^, M. Bernandini, Dipl. ECVN, Associate Professor^2,3^, F. Fidanzio, PhD^1^ , V. Buffagni, Neurology Intern^1^, E. Bianchi, Dipl. ECVN, Associate Professor^1^

##### ^1^Dept.of Veterinary Science, University of Parma, Parma, Italy; ^2^Neurology Unit, Anicura Portoni Rossi Veterinary Hosipital, Bologna, Italy; ^3^Department of Animal Medicine, Production and Health, Clinical Section, University of Padua, Legnaro, Padua, Italy

Two female Boxer dogs from the same litter were presented at 2‐months of age for urinary and fecal incontinence. Both dogs had an abnormal tail consisting of a small stump, an atonic anal sphincter and absent perineal reflex and sensation. Electrodiagnostic evaluation showed suspect neurotmesis of pudendal nerves. Radiology and CT scan of the spine displayed similar findings in both dogs: 6 lumbar vertebrae followed by a lumbosacral transitional vertebra, lacking a complete spinous process, and a hypoplastic vertebra carrying two hypoplastic sacral transverse processes as the only remnant of the sacral bone; caudal vertebrae were absent. At MRI, one dog had a dural sac occupying the entire spinal canal and ending in a subfascial fat structure. In the other dog, the dural sac finished in an extracanalar, subfascial, well‐defined cystic structure, caudal to the spine and likely communicating with the subarachnoid space, consistent with meningocele. The owners declined surgical treatment. During the 18‐months follow‐up, the dogs did not show changes in clinical signs except for recurrent cystitis. Sacro‐caudal dysgenesis is a malformation of the sacrum, caudal vertebrae and the corresponding spinal cord segments. Similar findings are described in humans in the Currarino syndrome, characterized by a triad of congenital anomalies that are not consistently present together: anorectal malformations, sacrococcygeal osseous defects, and various types of presacral masses (meningocele, enteric cysts or teratomas). To the Authors' knowledge, this is the first report describing sacro‐caudal dysgenesis in dogs. Investigations are underway to identify the genetic basis of this condition.

## (P 82)

### Multiple coccygeal disk herniation in a dog

#### S. Papageorgiou, DECVN^1^ , H. Gaillot, DECVDI^1^ , K. Gnirs, DECVN^1^ , L. Arti, DVM, resident^1^

##### ^1^Advetia, velizy villacoublay, France

Multiple coccygeal disk herniation in a dogA 5‐year‐old neutered male Beagle dog was evaluated for a 3‐week history of low tail carriage. On the basis of pain elicited during palpation of the sacral area and tail manipulation without any other abnormalities in postural reactions and spinal reflexes including flexion of the hind limbs, anal tone and perineal reflex, the lesion was most likely localized to the sacral or cranial coccygeal vertebrae (S1‐S3 or Cd1‐Cd2 radiculopathy). In our case, epidemiologic factors (middle‐aged chondrodystrophic breed dog) and lack of reported trauma were in favor of an intervertebral disk herniation. Spinal computed tomography (CT) examination findings included two separate well‐delineated mineral density structures extending from the ventral region of the vertebral canal through the right Cd1‐Cd2 and right Cd2‐Cd3 intervertebral foramen with an almost complete obliteration of both foramen, a not displaced mineralized Cd3‐ Cd4 disc, mild ventral spondylosis deformans of the Cd2‐Cd. Coccygeal intervertebral disk herniation appears to be a rare pathology in dogs, with only nine cases reported in the literature. Various treatment modalities have been described. In the current case a right‐sided hemilaminectomy was performed at the level of Cd1–Cd2 and Cd2–Cd3 intervertebral spaces. Therefore, this is the first case reporting multiple coccygeal intervertebral disk extrusions in a dog with involvement of the Cd2–Cd3 intervertebral disk space along with the previously described Cd1–Cd2 intervertebral disk space. In conclusion, coccygeal intervertebral disk herniation should be considered in dogs that are presented with caudal vertebral pain, pain upon tail manipulation or during defecation. Whatever the technique, decompressive surgery should be considered when clinical signs are unresponsive to medical treatment, with excellent prognosis.

## (P 83)

### Phosphorylated neurofilament heavy levels in serum and cerebrospinal fluid of dogs: Preliminary data using two ELISA assays of different sensitivity

#### V. Buffagni, Neurology Intern^1^, D. Dell'Apa, Resident ECVN^1^ , V. Cavalli, DVM, PhD^1^ , M. Crotti, DVM^1^ , F. Tummolo, PhD^2^ , E. Bianchi, Dipl. ECVN, Associate Professor^1^, R. Saleri, PhD, Associate Professor^1^

##### ^1^Dept. of Veterinary Science, University of Parma, Parma, Italy; ^2^Euroimmune Italia, Padua, Italy

Neurofilament proteins are promising biomarkers of neurodegeneration and neuronal injury. In particular, elevated phosphorylated neurofilament heavy (pNF‐H) levels have been reported in dogs with degenerative myelopathy (DM) and spinal cord injury. Despite the reduced concentrations of pNF‐H compared to CSF, that may affect the reliability of the results, serum represents the ideal target biological fluid to track the progression of neuroaxonal damage. The aim of this study was to evaluate pNF‐H concentrations in CSF and serum of dogs with DM and normal dogs using two ELISA assays with different sensitivity. Five dogs with suspect DM (DM group) and 5 normal dogs (C group) were included in the study. DM dogs had clinical history, neurological examination and advanced imaging consistent with DM, and were homozygous for 118G>A SOD1 mutation. A “standard” and a high‐sensitive pNf‐H ELISA Kit (Euroimmun, Germany) were applied to CSF and serum, respectively. Median CSF concentration of pNF‐H was 48.7 ± 13.7 ng/mL in the DM group and 2.2 ± 0.7 ng/mL in C group. Median serum concentration of pNF‐H was 0.443 ± 0.147 ng/mL in the DM group and 0.187 ± 0.128 in C group. Based on these preliminary results, the highly sensitive ELISA kit shows promise to differentiate normal from increased levels of pNF‐H in serum. Indeed, pNF‐H concentrations showed the same trend in serum and CSF. Once validated in the dog, high‐sensitive pNF‐H determination in serum may become an useful, minimally invasive, laboratory test for clinical and research activity on neurodegenerative disorders and spinal injury.

## (P 84)

### Clinical course and MRI lesion progression over four years in a dog with final diagnosis of high‐grade oligodendroglioma in the forebrain

#### F. Graciolli Tomazi, Med Vet^1^, V. M. Stein, Prof, Dr, Med Vet, PhD, Dipl. ECVN^1^ , A. Oevermann, Prof, Dr, Med Vet, Dipl. ECVP^2^ , F. Meneses, Med Vet, Dipl. ECVDI^3^ , A. Maiolini, Med Vet, PhD, Dipl. ECVN^1^

##### ^1^Division of Clinical Neurology, Dept. of Clinical Veterinary Medicine, Vetsuisse Faculty, University of Bern, Bern, Switzerland; ^2^Division of Neurological Sciences, Dept. of Clinical Research and Veterinary Public Health, Vetsuisse Faculty, University of Bern, Bern, Switzerland; ^3^Division of Clinical Radiology, Dept. of Clinical Veterinary Medicine, Vetsuisse Faculty, University of Bern, Bern, Switzerland

A 9‐month‐old female intact Cavalier King Charles Spaniel was presented with a 3‐month history of paroxysmal episodes of pain and cervical scratching. MRI revealed a large left frontal lobe intra‐axial complex cystic lesion with faint incomplete rim enhancement, transtentorial and transforaminal brain herniation, and cervical syringomyelia. A primary brain tumor (glioblastoma or anaplastic astrocytoma), or less likely a parasitic cyst where suspected. Palliative treatment (dexamethasone, omeprazole, gabapentine) reduced the frequency of the paroxysmal episodes. On MRI recheck 30 months later, the lesion showed comparable size, evidence of hemorrhage or mineralization, and regressive signs of increased intracranial pressure and perilesional edema. Considering the clinical evolution and rather benign biological behavior, porencephaly, congenital cystic malformations or less likely a parasitic cyst where then considered as differentials for the lesion. The patient remained clinically stable for another 19 months until generalized, tonic‐clonic, epileptic seizures acutely occurred. The neurological status subsequently deteriorated over 48 hours to stupor, cardiovascular arrest and eventually death. Post‐mortem MRI showed lesion progression characterized by increased size, increased soft tissue component, invasion of the left lateral ventricle, transtentorial and transforaminal brain herniation. Neuropathology revealed an anaplastic oligodendroglioma (WHO grade III) with subarachnoidal and intraventricular drop metastasis. Although oligodendroglioma is a common primary intracranial tumor in dogs, survival data are scarce because of lack of systematic and prospective follow‐up studies. This is the first report describing the long‐term clinical course and MRI lesion progression under palliative treatment of a dog eventually diagnosed with high‐grade oligodendroglioma.

## (P 85)

### Facial twitches beyond epilepsy in rhodesian ridgebacks: Axonal neuropathy and myofiber hyperfunctionality

#### L. Lemke, Dr^1^, H. A. Volk, Prof, Dr, PhD^1^ , K. Matiasek, Prof, Dr^2^, N. Meyerhoff, Dr^1^

##### ^1^Department of Small Animal Medicine and Surgery, University of Veterinary Medicine Hannover, Hannover, Germany; ^2^Section of Clinical & Comparative Neuropathology, Ludwig‐Maximilians‐Universitaet, Munich, Germany

Juvenile Myoclonus Epilepsy (JME), an inherited epilepsy, is caused by a deletion in the DIRAS1‐gene in young Rhodesian Ridgebacks and characterized by frequent myoclonic twitches. Levetiracetam often is effective in these patients. Two 2.5 years‐old male Rhodesian Ridgebacks, tested negative for DIRAS1‐deletion, were examined: One dog was presented with a 5‐month history of focal, bilateral high frequency twitching of the eyelids and intermittently of both thoracic limbs. Magnetic Resonance Imaging (MRI) of the brain and cerebrospinal fluid (CSF) examination were unremarkable. Neospora caninum antibody titer was 1:100, but PCR in CSF was negative. Treatment with Levetiracetam, phenobarbitone, prednisolone and doxycycline showed no improvement. In electromyography pathological spontaneous activity and prolonged insertion potentials were found. Neuropathological investigation of a muscle‐nerve‐biopsy revealed a progressive, axonal neuropathy of yet unclear cause. The second dog also presented with constant bilateral, high frequent blinking and twitching of the eyelids and flews for 1 year nearly constantly with worsening in stressful situations. Levetiracetam caused no improvement. Brain MRI, electromyography including eyelids and motor nerve conduction velocity were unremarkable. CSF examination detected insignificant elevation of the protein content. The antibody titer against Neospora caninum was 1:800, but PCR in CSF was negative. Pathologically, a hyperfunctional myofibre hypertrophy was suspected upon investigation of muscle‐nerve biopsy.

## (P 86)

### Diagnosis and treatment of spinal T‐cell‐rich large B cell lymphoma in a cat

#### L. Saunders, BVSc^1^ , O. Davies, MAVetMb, MVetMed, DipACVIM (Oncology)^1^, G. Nye, BVetMed, MVSc, DipECVN^1^

##### ^1^Highcroft Veterinary Referrals, Bristol, UK

Introduction/Purpose: A 3‐year‐old, male neutered domestic shorthair cat presented for investigation of acute, progressive ambulatory paraparesis. This case report, as far as the authors are aware, is the first reported case of feline spinal T‐cell‐rich large B cell (TCRLBC) lymphoma. Methods: Retrospective case report. Magnetic resonance imaging (MRI) of the thoracolumbar spine, cerebrospinal fluid analysis (CSF) and cytology were initially performed, later followed by surgical decompression by L3–L5 hemilaminectomy and biopsy, with histology and immunohistochemistry performed on the resected tissue. Following surgical debulking, chemotherapy commenced with five cycles of lomustine, cytarabine and vincristine with oral prednisolone therapy and three doses of l‐asparaginase. Results: MRI identified a well‐defined extra‐dural contrast‐enhancing left‐lateralised mass at the level of the L4 vertebral body. CSF identified a non‐specific mild mixed cell pleocytosis. TCRLBC lymphoma was diagnosed with immumohistochemistry. Clinical remission within 36 weeks and remaining without clinically detectable lymphoma at the time of submission (81 weeks). Discussion/Conclusion: This case report supports definitive diagnosis and combination therapy for specifically TCRLBC lymphoma in cats. Following surgical decompression, the cat improved neurologically within 3 days. This case is comparable to reported median survival times (MST) of TCRLBC lymphoma in cats of other locations treated with a combination of chemotherapy agents (1.2 years.) The veterinary literature is limited in terms of treatment for spinal lymphoma in cats. Traditionally the assumed prognosis of CNS lymphoma in cats is poor, but this case offers a fairer prognosis in a specific type of spinal lymphoma. In conclusion, not all myoclonic jerks in Rhodesian Ridgebacks are caused by JME. If DIRAS1‐testing is negative and the dogs do not respond to Levetiracetam, further diagnostics including muscle‐nerve‐sampling is indicated and therapy must be adapted to the underlying cause.

## (P 87)

### Epidural idiopathic sterile pyogranulomatous steatitis in a cat

#### A. Martinez, DVM^1^ , J. Mortier, DVM, Dip. ECVDI^2^ ,^3^, R. Gonçalves, DVM, Dip. ECVN^3^ , E. Ricci, DVM, PhD, Dip. ACVP^4^ , E. Alcoverro, DVM, Dip. ECVN^35^

##### ^1^Department of Clinical Veterinary Medicine, Queens Veterinary School Hospital, University of Cambridge, Madingley Road, Cambridge, United Kingdom; ^2^Centre Hospitalier Universitaire Vétérinaire d'Alfort, École Nationale Vétérinaire d'Alfort, Maisons‐Alfort, France; ^3^Small Animal Teaching Hospital, School of Veterinary Science, Institute of Infection, Veterinary and Ecological Sciences, University of Liverpool, Neston, United Kingdom; ^4^Department of Veterinary Anatomy, Physiology and Pathology, School of Veterinary Science, Institute of Infection, Veterinary and Ecological Sciences, University of Liverpool, Neston, United Kingdom; ^5^ChesterGates Veterinary Specialists, Units E & F, Telford Court, Gates Lane, Chester, United Kingdom

Epidural idiopathic sterile pyogranulomatous steatitis (EISPS) is an inflammation of the epidural fat; it has been occasionally reported in dogs, but it is extremely rare in cats. In this case, we describe the clinical presentation and the imaging, clinicopathological, surgical and histopathological findings and follow‐up of a cat with spinal EISPS. A four‐month‐old male domestic shorthair cat was presented with acute onset progressive and severe signs of T3–L3 myelopathy. MRI revealed bilateral, strong T2W and T1W hyperintensity and marked contrast‐enhancement of the articular processes of T12 and T13 vertebrae, the surrounding epaxial musculature and the epidural fat. The latter was dorsally compressing the spinal cord. There also was focal T2W hyperintense intramedullary signal with patchy contrast enhancement. CT of the thorax, abdomen and vertebral column revealed bilateral cortical and subchondral osteolysis of the zygapophyseal facet joints of T12–T13. CSF analysis revealed neutrophilic pleocytosis. Surgical decompression was achieved with T12–T13 dorsal laminectomy. Prominent dorsal epidural fat causing spinal cord compression was removed and revealed a visibly swollen and discolored spinal cord. Histopathological examination of the epaxial musculature, T12–T13 dorsal laminae, and epidural fat revealed subacute suppurative diffuse and severe steatites and chondritis. Culture did not yield growth of infectious agents. Upon follow up 8 months after the surgery, the cat showed only mild residual ataxia of the pelvic limbs. Spinal EISP should be considered as differential diagnosis of thoracolumbar myelopathy in young cats. In this case, the long‐term outcome was favorable after surgical decompression.

## (P 88)

### Clinical and imaging findings in a suspected inmunomediated L6‐L7 radiculoneuritis and extraocular myositis associated to leishmania infection in a dog

#### J. M. Segura, Lv^2^, T. Heredia, Lv^1^, A. Costas, Lv^1^, Á. Ortillés, Lv, dip. ECVO^1^ , S. Ródenas, Lv, dip. ECVN^1^

##### ^1^Anicura Valencia Sur Hospital, Valencia, Spain; ^2^Menescal Hospital, Novelda, Spain

Leishmaniasis is a parasitic disease that causes tissue damage through different mechanisms: granulomatous inflammation, deposition of immunocomplexes and/or production of autoantibodies. We present clinical signs, diagnostic test, and treatment response of a patient with suspected immune‐mediated radiculoneuritis and extraocular myositis, associated to Leishmania infantum. A 6‐year‐old male non castrated mix breed dog was presented for further investigations of 48 hours evolution of non‐ambulatory paraparesis. Neurological examination revealed delayed postural reactions and decreased spinal reflexes in both pelvic limbs. These clinical signs were consistent with a L4–S2 neurolocalisation. He also had right eye exophthalmia with increased resistance to retropulsion, lateral deviation of the visual axis and mydriasis. Comprehensive blood test revealed hyperproteinemia/hyperglobulinemia as the only important finding. Leishmania serology showed a high positive title 1/320. MRI showed a bilateral thickening of the ventral internal venous plexus, along the vertebral body of L6, which showed marked and homogeneous contrast enhancement in post‐contrast T1W, with associated compression of the cauda equina. This contrast enhancement extended into both intervertebral foramina at L6–L7 following the course of the thickened spinal nerves out of the vertebral canal along the hypaxial paravertebral musculature. Right extra‐ocular muscles were thickened with moderate and homogeneus contrast enhancement. Based on diagnostic test the main diagnostic suspicion was inmune‐mediated radiculoneuritis and extraocular myositis associate to Leishmania infection. Treatment with leishmanicidal drugs (Meglumine antimoniate) was instituted together with inmunomodulatory drugs (Prednisone and Cyclosporine), observing a complete recovery of the patient with resolution of MRI and ocular findings.

## (P 89)

### A comparative treatment trial of two add‐on treatment stragegies in dogs with difficult‐ to‐treat idiopathic epilepsy

#### S. R. P. Kriechbaumer, DVM^1^ ,^2^, K. Jurina, DECVN, Dr. Med Vet^2^, F. Wielaender, DECVN, Dr. Med Vet^1^, H. C. Schenk, DECVN, Dr. Med Vet, Ph.D.^3^, T. A. Steinberg, DECVN, Dr. Med Vet^2^, S. Reese, Dr. Med Vet, Dr. habil^4^, G. Buhmann, Dr. Med Vet^1^, S. Doerfelt, Dr. Med Vet^1^,^2^, H. Potschka, Prof.^5^, F. Andrea, Prof., DECVN, DACVIM^1^

##### ^1^Clinic of Small Animal Medicine, LMU Munich, Munich, Germany; ^2^AniCura Small Animal Clinic Haar, Haar, Germany; ^3^Small Animal Clinic Lüneburg, Lüneburg, Germany; ^4^Institute for Anatomy, Histology and Embryology, LMU Munich, Munich, Germany; ^5^Institute for Pharmacology, Toxicology and Pharmacy, LMU Munich, Munich, Germany

Idiopathic epilepsy is a common neurological disorder. Only few studies are available to guide treatment choices beyond licensed veterinary drugs. The aim of the study was to compare efficacy of two add‐on treatment strategies in dogs with difficult‐to‐treat idiopathic epilepsy. Twenty‐six dogs with idiopathic epilepsy (tier 1 or 2) and monthly seizures despite treatment with two or more antiseizure medications were allocated to pregabalin 4 mg/kg BID (14 dogs) or modification of levetiracetam (increase in dose, 12 dogs) add‐on treatment by stratified randomization. Dog owners were offered to participate for 4 months or longer and minimum to the third day with tonic‐clonic seizures after titration phase. Extension of the interseizure interval compared to the longest interseizure interval during baseline and time to the third seizure day were compared between groups. Dogs with a 3‐fold extension of the interseizure interval or seizure freedom >3 months were considered responders. Treatment groups did not differ in the overall effect on the interseizure interval (*P* = 0.681) and time to the third tonic‐ clonic seizure day (*P* = 0.334). Two dogs from the pregabalin group (14.3%; 2/14) and one dog from the levetiracetam group (8.3%; 1/12, *P* = 1.000) were responders with long seizure‐free intervals (122–219 days) but then relapsed to their initial seizure frequency after 10 months. The study provides information on response rates for two add‐on treatment strategies in dogs with chronic, difficult‐ to‐treat idiopathic epilepsy. We observed excellent compliance with the novel study protocol and the use of individual study endpoints.

## (P 90)

### Retrospective evaluation of clinical presentation, imaging findings and outcome of french bulldogs diagnosed with thoracolumbar intervertebral disc extrusion

#### S. Butterfield, BSc(Hons), BVSc, PGDip(VCP), MRCVS^1^ , A. Hall^1^, Y. López Barroso, LV, MRCVS^2^ , D. Whittaker, BSc(Hons), BVetMed(Hons), MVetMed, PhD, DipECVN, MRCVS^2^ , A. Crawford, BVM&S, BSc, PhD, DipECVN, MRCVS^1^ , J. Fenn, BVetMed, MVetMed, DipECVN, FHEA, MRCVS^1^

##### ^1^Queen Mother Hospital for Animals, Clinical Science and Services, Royal Veterinary College, Hatfield, United Kingdom; ^2^Fitzpatrick Referrals Orthopedics and Neurology, Surrey, United Kingdom

Thoracolumbar intervertebral disc extrusion (T‐IVDE) is a common cause of pain and paresis in dogs, with French Bulldogs (FBs) frequently affected. The aim of the study was to describe the clinical presentation, advanced imaging findings, surgical approach and outcome of a large cohort of French Bulldogs diagnosed with T‐IVDE. This retrospective study used electronic medical records from two referral hospitals searched between January 2010 and January 2021 for FBs diagnosed with T‐IVDE, with computed tomography or magnetic resonance imaging studies available. Records were searched for patient descriptive variables, clinical presentation, examination findings, diagnostic imaging studies, treatment (medical or surgical) and outcome where available. 301 FBs (110 female, 191 male) were included with median age of onset of 43.3 months (12‐86 months). Signs were typically acute (173/301, 57%) or sub‐acute (87/301, 29%) in onset. 123/301 (41%) FBs were paraplegic on presentation, 50 of which (40%) were deep pain perception negative. Respiratory distress requiring medical intervention was a frequent complication. The most common disc affected was L2‐L3 (68/301, 23%) with 209 extrusions (69%) occurring between L1 and L5. 259 FBs underwent decompressive hemilaminectomy, of which 138 (53%) were multiple‐space. Of the remaining 42 FBs, 11 were euthanased without treatment and 31 were managed conservatively. 21/46 dogs (46%) that were deep pain negative were euthanased: 8 (38%) at the time of diagnosis and 6 (29%) due to a suspicion of progressive myelomalacia. These results suggest an acute and severe phenotype of IVDE within the FB breed, however further investigation is required to confirm this.

## (P 91)

### Alternaria infectiora encephalitis in a dog

#### C. Escriou, DMV,PhD, Ass. Prof^1^, E. Ramery, DMV, ECVCP^1^ , M. K. Kacem, DMV^1^ , F. Dimeglio, MRI Phys^2^, S. Belluco, DMV, ECVP^1^ , J. Guillot, DMV, PhD, EVPC, Prof^3^

##### ^1^Univ Lyon VetAgro Sup, Marcy l'étoile, France; ^2^Hawkcell, Marcy l'étoile, France; ^3^Ecole Natione Veterinaire d'Alfort, Maisons‐Alfort, France

Alternaria species are dematiaceous hyphomycetes that are distributed worldwide and are commonly found on the coat of both dogs and cats. They belong to a group of opportunistic fungi that causes mainly skin infection in immunosuppressed patients. Although only few cases have been described in humans and only one in the dog, authors agree that *Alternaria infectoria* infection could be an emerging phaeohyphomycosis of occidental countries. A 5 years old German Shepherd with no medical history was presented for a 15 days duration of wobbling gait. Clinical examination was unremarkable and neurological examination was consistent with cerebellar involvement. Brain MRI revealed a voluminous diffuse ill‐defined intra‐axial cerebellar lesion, hyperintense on T2‐weighted and Flair imaging and partially enhanced after gadolinium injection, suitable with tumoral or inflammatory lesion. CSF was normal. As CNS lymphoma was evoked, thoracic radiography and abdominal echography with liver and spleen fine needle aspiration were realized. Spleen cytology revealed sprouting forms in macrophages evocating systemic fungal infection. Cultures of spleen biopsy grew *Alternaria* sp., later identified by polymerase chain reaction as *A. infectoria*. Fluconazole and terbinafine with low doses of prednisolone were given for 4 weeks and allowed only partial recovery. Due to uncertain prognosis the owners asked for euthanasia. Brain histology confirmed the diagnosis of fungal infection with septated fungal hypha observed in the center of multiple inflammatory and granulomatosis lesions focused on the cerebellum and the brain stem. To our knowledge *A. infectoria* encephalitis was neither described in dog. This case of systemic Alternaria infectoria infection in a good shape dog seems to confirm its pathogenic power and raises questions about this emerging phaeohyphomycosis.

